# Short-Half-Life Chemicals: Maternal Exposure and Offspring Health Consequences—The Case of Synthetic Phenols, Parabens, and Phthalates

**DOI:** 10.3390/toxics12100710

**Published:** 2024-09-29

**Authors:** Delphine Rousseau-Ralliard, Jeanne Bozec, Marion Ouidir, Nicolas Jovanovic, Véronique Gayrard, Namya Mellouk, Marie-Noëlle Dieudonné, Nicole Picard-Hagen, Maria-José Flores-Sanabria, Hélène Jammes, Claire Philippat, Anne Couturier-Tarrade

**Affiliations:** 1 Université Paris-Saclay, UVSQ, INRAE, BREED, 78350 Jouy-en-Josas, France; 2 Ecole Nationale Vétérinaire d’Alfort, BREED, 94700 Maisons-Alfort, France; 3 University Grenoble Alpes, Inserm U1209, CNRS UMR 5309, Team of Environmental Epidemiology Applied to Development and Respiratory Health, Institute for Advanced Biosciences, 38000 Grenoble, France; 4 ToxAlim (Research Center in Food Toxicology), Université de Toulouse, INRAE, ENVT, INP-Purpan, UPS, 31062 Toulouse, France

**Keywords:** endocrine disruptors, gestation, placenta, maternal exposure, offspring phenotype, epigenetics, cardiometabolic outcomes, fertility, adulthood

## Abstract

Phenols, parabens, and phthalates (PPPs) are suspected or known endocrine disruptors. They are used in consumer products that pregnant women and their progeny are exposed to daily through the placenta, which could affect offspring health. This review aims to compile data from cohort studies and in vitro and in vivo models to provide a summary regarding placental transfer, fetoplacental development, and the predisposition to adult diseases resulting from maternal exposure to PPPs during the gestational period. In humans, using the concentration of pollutants in maternal urine, and taking the offspring sex into account, positive or negative associations have been observed concerning placental or newborn weight, children’s BMI, blood pressure, gonadal function, or age at puberty. In animal models, without taking sex into account, alterations of placental structure and gene expression linked to hormones or DNA methylation were related to phenol exposure. At the postnatal stage, pollutants affect the bodyweight, the carbohydrate metabolism, the cardiovascular system, gonadal development, the age of puberty, sex/thyroid hormones, and gamete quality, but these effects depend on the age and sex. Future challenges will be to explore the effects of pollutants in mixtures using models and to identify the early signatures of in utero exposure capable of predicting the health trajectory of the offspring.

## 1. Synthetic Phenols, Parabens, and Phthalates

The human population is continually exposed to a wide range of chemical compounds present in the environment, including phenols, parabens, and phthalates (PPPs), which can affect human health.

The most studied phenol is bisphenol A (BPA, 2,2-bis [4-hydroxyphenyl]propane), a synthetic chemical used in consumer products such as the lining of canned food and drinks, the packaging of baby formula and baby bottles, and dental implants [1]. BPA can migrate from cans into food, from plastic bottles into water, or from dental fillings into saliva, leading to an oral exposure. In addition, BPA can penetrate the skin through thermal paper in sales receipts and by inhalation via industry. Despite its ban from plastic baby bottles in 2011 in Europe (https://eur-lex.europa.eu/legal-content/EN/TXT/PDF/?uri=CELEX:32011R0321&from=EN, accessed on 12 September 2024) and from all food-grade materials in France from 1 January 2015 (https://eur-lex.europa.eu/legal-content/EN/TXT/PDF/?uri=CELEX:32018R0213&from=EN, accessed on 12 September 2024), the exposure to BPA is still widespread in the global population [2]. In the body, BPA is quickly metabolized into BPA-glucuronide in the liver and then mainly eliminated by the kidneys. Noteworthy is that the unconjugated BPA is the bioactive form. BPA has been described as mimicking estrogen by binding to its receptors, leading to its classification as a “reproductive toxicant” in 2016 (https://www.foodpackagingforum.org/news/bpa-classified-as-reproductive-toxicant, accessed on 12 September 2024), and then as an endocrine disruptor in 2018 (https://www.foodpackagingforum.org/news/bpa-identified-as-environmental-endocrine-disruptor, accessed on 12 September 2024), by the European Chemicals Agency (ECHA). BPA is known to induce metabolic disorders in human and animal models, reproductive outcomes, the development of cardiovascular and neurodegenerative diseases, cancer, and genotoxic effects [3,4], but its effect during key periods such as preconception or gestation on the phenotype of first- and second-generation offspring using epidemiological data or animal models is still needed.

Since the banning of BPA in many consumer products [1], substitutes have been proposed by manufacturers over the last decade, including bisphenol S (BPS, 4,4′-sulfonyldiphenol) and bisphenol F (BPF, 4,4′-dihydroxydiphenylmethane). BPS and BPF structures are similar to BPA, suggesting that they can act as endocrine disruptors as well. They have already been shown to cause obesity effects/metabolic effects in animal models [4], but further experiments are needed to assess their impact on organ function and also during pregnancy on the phenotype of the offspring, as these compounds have only been used for a short time.

The phenol family also includes triclosan (TCS, 5-chloro-2-(2,4-dichlorophenoxy) phenol), a synthetic antimicrobial agent [5]. TCS is used in several personal care products, including toothpaste, antibacterial soaps, dishwashing liquids, deodorant soaps, cosmetic and antiseptic products, and deodorants. TCS is also used in kitchen utensils, toys, bedding, clothes, fabrics, and trash bags. The main route of human exposure is dermal exposure [6], but exposure can also occur through the consumption of water and/or food products [7]. TCS is metabolized to glucuronide and sulfate conjugates, and is mainly excreted in the urine in humans [8]. Several investigators have reported that TCS is an EDC that affects the immune system, and cardiovascular and reproductive functions [7,9,10,11,12]. Thus, the percentage of TCS in certain products has been regulated by the EU, and it is also banned in shaving products [13]. Since TCS is used in many products, it is important to know the effects of direct exposure to this molecule on the phenotype as well as the potential intergenerational effects.

Parabens are considered as endocrine disruptors [14] and used to prevent the growth of microorganisms in personal care products such as lotions, deodorants, hair care products, shaving products, pharmaceuticals, textiles, and clothes, but also in food additives such as methylparaben (MeP, food additive E218), ethylparaben (EtP, E214), and, to a lesser extent, propylparaben (PrP, E216), heptylparaben (HeP), butylparaben (n-BuP), and isobutylparaben (i-BuP) [14]. The estimated total intake of parabens from foods achieves 307–940 ng/kg bodyweight [15]. The most common parabens investigated and detected in biological fluids are MeP, EtP, n-PrP, and n-BuP, whereas i-BuP, isopropylparaben (i-PrP), and benzylparaben (BzP) are less investigated. Moreover, the urine concentrations of parabens are higher in women compared to men. Therefore, it is essential to test the effects of exposure to all of these molecules on the phenotypes at key stages of development and during pregnancy so to evaluate the effects on the offspring.

Phthalates are the main plasticizers used in the industry to improve the extensibility, elasticity, and workability of polymers. Phthalates include low-molecular-weight di-n-butyl phthalate (DBP), benzyl butylphthalate (BBP), di-(2-ethylhexyl) phthalate (DEHP), di-methyl phthalate (DMP), and diethyl phthalate (DEP), or high-molecular-weight di-isononyl phthalate (DiNP) and di-isodecyl phthalate (DiDP). More than 25 phthalates are used in many consumer products, such as toys, clothing, inks, rubbers, adhesives, paints, household materials, pesticides, cosmetics, personal care products, pharmaceutical products, etc. [16,17,18]. They are present in our environment and are able to reach the food and beverage chain by migration during processing, storing, transportation, and preparation. Even if ingestion is considered the main source of human exposure, dermal or nasal routes are not negligible. Several phthalates have been classified by the European Commission as having endocrine-disrupting or reprotoxic properties (category 1B). In the EU, several phthalates are now banned from use in cosmetics, including BBP, DEHP, DBP, and bis(2-methoxyethyl) phthalate (DMEP) (EC/1223/2009). The use of several compounds is also regulated in material intended to come into contact with food (DEHP, DBP, and BBP, EC/2007/19) as well as in toys and childcare articles (DEHP, DBP, BBP, DiNP, DiDP, and di-n-octyl phthalate (DnOP), French law 2006-1361) [16,18,19]. In Europe, the REACH list limits the level of exposure to DEHP metabolites, including (mono(2-ethyl-5-oxohexyl) phthalate (MEOHP), MEHP, and mono(2-ethyl-5-hydroxyhexyl) phthalate (MEHHP), within 0.02 mg/kg bw/d. Noteworthy, the EFSA has set the tolerable daily intakes (in mg/kg of bw) to 0.05 for DEHP, 0.01 for DBP, 0.15 for DiNP and DiDP, and 0.5 for BBP [20,21]. The half-life of phthalate diesters in blood plasma or urine is less than 24 h, and these compounds are mainly excreted in the urine as conjugated monoesters [22], but some of them, such as DEHP, undergo a secondary metabolism before urinary excretion. Consequently, urine is the more relevant matrix for the biomonitoring of human phthalate exposure [18], even if all the metabolites of a parent molecule cannot be assayed in this fluid for technical reasons. Exposure to phthalates is raising a great concern regarding their impact on human health. For several years, accumulating evidence has suggested that phthalates and their metabolites are suspected to act as EDCs through their interaction with different signaling pathways. Phthalate exposure has been correlated to several health outcomes, among them adverse reproductive disorders in women and men, neurological diseases, cancer, metabolic disorders, asthma, allergies, and toxicity in both the liver and kidney [16], but the intergenerational effects should be investigated further with doses mimicking the human exposure.

In this review, we will focus on PPPs, i.e., their metabolism, the toxicokinetic models for adapting human exposure doses to animal models, and the transplacental transfer of PPPs. The effects of PPP exposure throughout critical periods such as preconception, gestation, and lactation on offspring health, including the placental function, neonatal phenotypes, metabolic outcomes, cardiometabolic disorders, and gonadal functions, will be presented in the context of the developmental origins of health and disease (DOHaD) concept [23]. These data will be based on the literature from epidemiological data, associating human maternal urinary concentrations of these molecules and their metabolites with the health effects on the offspring, and with regard to the long-term postnatal cardiometabolic effects, they will be mainly presented through studies on animal models, since human cohorts currently rarely explore the effects beyond childhood.

## 2. Absorption, Distribution, Metabolism, and Excretion Processes of PPPs

PPPs are considered non-persistent chemicals because absorbed PPPs are rapidly metabolized and primarily excreted in the urine. In humans, ingested BPA and its analogs are rapidly and efficiently absorbed, and almost entirely excreted in the urine as a conjugate [24,25,26,27].

Following oral administration to human volunteers, TCS is rapidly absorbed, metabolized to conjugates, and eliminated in the urine within 4 days of exposure, with a terminal plasma half-life of 21 h [28]. Toxicokinetic studies performed in rats have shown that benzophenone-3 (BP-3) is rapidly absorbed after oral exposure and mainly excreted in the urine in conjugated forms [29]. BP-3 can also be transformed into three major metabolites, namely, benzophenone-1 (BP-1), benzophenone-8 (BP-8), and 2,3,4-trihydroxybenzophenone (THB), with BP-1 being the most frequently detected in rats [30,31]. Parabens are well absorbed following oral or dermal exposure and extensively metabolized through hydrolysis into para-hydroxybenzoic acid (PHBA), which is excreted in the urine mainly as glucuronide and sulfate conjugates. A fraction of absorbed parabens is also excreted in the urine in the form of glucuronide and sulfate conjugates [32]. Since PHBA is a non-specific metabolite of parabens, several biomonitoring studies have assessed the human exposure by measuring the urinary concentrations of conjugated parabens.

For phthalates, mono-n-butyl phthalate (MBP), mono-isobutyl phthalate (MiBP), and mono-benzyl phthalate (MBzP) are the main urinary metabolites of their respective parent short-chain phthalates, including di-n-butyl phthalate (DnBP), diisobutyl phthalate (DiBP), and BBP, respectively. The monoester metabolites of long-chain phthalates, such as DEHP and DiNP, MEHP, and MiNP, respectively, give rise to secondary oxidative metabolites (mono-2-ethyl-5-hydroxy-hexyl phthalate (MEHHP), mono-2-etyl-5oxohexyl phthalate (MEOHP), mono-2-ethyl-5-carboxypentyl phthalate (MECPP), and mono [2-(carboxymethyl)hexyl] phthalate (MCMHP)) for DEHP, and several complex isoforms of monoisononyl phthalate (MiNP) obtained by the formal condensation of one of the carboxy groups of phthalic acid with the hydroxy group of isononanol, which are the main xenobiotic metabolites excreted in human urine either as free or conjugated compounds [33].

Because PPPs are rapidly metabolized and excreted in the urine, several biomonitoring studies have assessed the human exposure by measuring the urinary concentrations of total (unconjugated plus conjugated) phenols and phthalate monoesters. Indeed, urine represents a relevant biological matrix for the biomonitoring of non-persistent chemical substances such as PPPs because their concentrations in urine are higher than in plasma concentrations, and because its collection is non-invasive. Generalized human exposure to PPPs is therefore reflected by the high frequency of detection of phenols and phthalate monoesters in the urine [2,34,35,36,37,38].

## 3. Toxicokinetic Models for Adapting Human Exposure Doses to Animal Models

Toxicokinetics (TKs) provides critical information for integrating hazard toxicity and exposure assessments to determine the potential risk of exposure to PPPs in humans. TKs is needed for determining the dose/concentration range that should be used in animal or in vitro testing to reproduce the internal exposure that can be expected from realistic human external exposure scenarios.

### 3.1. Estimation of PPP Daily Intake

PPP urine biomonitoring data have been widely used as biomarkers of exposure for epidemiological purposes [36,39]. Since urine biomonitoring data provide an integrative measure of exposure regardless of the source and route, the measurement of urinary concentrations of total PPP metabolites allows for the direct assessment of the daily PPP intake (*DI*). The calculation of the *DI* from urinary biomonitoring data relies on the urinary excretion factors (Fue) of the measured PPPs or metabolites related to the parent chemical ingested (Equation (1), [40]), as follows:

(1) $$DI=\frac{UE\times UVnorm}{F_{UE}}\times MW$$

where *UE* is the molar urinary excretion of the measured PPPs or metabolites (µmol/L); *UV_norm_* is the daily excreted urinary volume (L/kg bw/day), estimated at 0.028 L per kg bw per day for a 70-kg bodyweight [41], and which should be adapted to the targeted population (i.e., pregnant women and infants); *F_UE_* is the molar ratio between the amount of PPPs or metabolites excreted in the urine and the amount of parent PPPs taken up; *MW* is the molecular weight of the parent PPPs.

For phenols, most biomonitoring studies rely solely on the determination of total phenol concentrations in the urine measured after enzymatic hydrolysis. While bisphenols and TCS are primarily eliminated in the urine as conjugates [25,28], the fraction of absorbed parabens excreted in the urine as glucuronides and sulfate conjugates ranges from about 6% for n-BuP to 17% for propylparaben (PrP) [42,43]. The selection of the appropriate biomarkers of exposure to long-chain phthalates such as DEHP and DiNP is more complex. Indeed, while approximately 70–85% of an oral dose of short-chain diester phthalates (DnBP, DiBP, BBP, and DEP) is excreted in the urine as hydrolytic monoester phthalates (MBP, MiBP, MBzP, and MEP) [44,45], only about 2 and 7% of an oral dose of DiNP and DEHP are excreted in the urine as the hydrolytic monoesters [40,46,47]. The secondary (oxidized monoester) phthalate metabolites of DiNP, such as OH-MiNP, oxo-MiNP, and cx-MiNP, and of DEHP, such as MECPP, MEOHP, MEHHP, and MCMHP, are the major metabolites excreted in human urine as free or conjugated compounds. Therefore, urinary concentrations of hydrolytic monoesters of long-chain relative to short-chain phthalates do not reflect the relative exposure to phthalates [48].

Although urine biomonitoring data provide essential information on the amount of compound that enters the body on a daily intake, regardless of the route of exposure, it is the concentration of the toxicologically active form of PPPs in the blood that determines the amount of chemicals that can reach the target tissues and receptors and exert effects.

For phenols, only the native (unconjugated) form is considered toxicologically active because it is capable of interacting with target tissues and receptors. Since phthalate diesters, such as DEHP, are cleaved to their hydrolytic monoesters in the mouth, stomach, intestines, or blood, plasma concentrations of phthalate diesters are expected to be very low [33]. It is the plasma concentrations of monoester phthalate, namely, MEHP, for DEHP that are considered relevant in terms of risk assessment [49].

### 3.2. Interspecies Extrapolation of PPP Dose

The steady-state plasma concentration (*C_SS_*) of the toxicologically active form of PPPs therefore represents the most relevant physiological variable for translating the results from animal studies to humans. *C_SS_* (ng/mL) is related to the dosing rate (mass per unit of time) by a key toxicokinetic parameter, namely, blood clearance (*CL*, volume per unit of time), in addition to the bioavailability (*F*, ranging from zero to one). This toxicokinetic parameter corresponds to the fraction of compound reaching the systemic circulation without change according to Equation (2), as follows:

(2) $$Dosing\;rate\;=CL\times C_{SS}/F$$

Using Equation (2), the PPP dosage to be administered orally to reproduce the targeted *C_SS_* of the toxicologically active form of PPPs in an animal species can therefore be calculated from the species’ *CL* and *F* values.

A loading dose (*LD*) of PPPs can be applied to enable serum concentrations to rapidly reach the targeted *C_SS_*. This dose is determined from the steady-state volume of the distribution (*Vss*) and *F* using Equation (3), as follows:

(3) $$LD=C_{ss}\times V_{ss}/F$$

By permitting the serial sampling of maternal and fetal blood after maternal or fetal administration, pregnant ewes provided a unique model for evaluating the disposition of several drugs during the prenatal period [50]. Among animal models, the rabbit represents an appropriate model for TK purposes. Like in humans, urinary elimination predominates in rabbits. This is unlike other species, such as rats and dogs, that have rather biliary excretion [51]. This interspecies difference is mainly observed for substances with a molecular weight between 300 and 600 Da, such as certain phthalate monoesters.

The main TK parameters of PPPs, i.e., *CL*, *Vss*, and *F*, can be determined from the plasma concentration–time profiles obtained following the intravenous and oral administrations of a mixture of EDCs. To achieve this, BPS and DEHP were administered by the oral route in a mixture of eight compounds in female rabbits to compute the loading and daily maintenance doses. The aim was to reproduce in rabbits the steady-state serum concentrations (*C_SS_*) of the two compounds found in pregnant women. Thus, the mean *C_SS_* of female rabbits measured after 17 weeks of exposure to a mixture of the eight compounds administered orally at doses deduced from the estimated TK parameters ranged from 0.77 to 1.21 ng/mL of the targeted *C_SS_* [52]. Toxicodynamic modeling in rabbits validated the interspecies extrapolation of human exposure rates to environmental contaminants.

## 4. Evaluation of Transplacental Transfer of PPPs Using Models

Understanding the materno-fetal transfer of PPPs across the placenta is crucial for assessing the prenatal exposure risks. To respond to this issue, the placental transfer of PPPs was investigated using the following two main models, which offer both advantages and disadvantages:

- The most clinically relevant model to evaluate the placental transfer of compounds is the ex vivo human placental perfusion model at term [53]. This enables placental transfer to be assessed by reproducing maternal and fetal circulation in a few hours and taking samples from each compartment. This model offers several advantages, as the placental barrier maintains its structural integrity and the separate perfusion of the maternal and fetal sides reproduces their respective blood flows. However, this physiological approach does not take into account the nonplacental toxicokinetic factors that could contribute to the level of fetal exposure. These factors include the maternal and fetal metabolism, the dynamic structural conditions of the pregnancy, and the physiological changes throughout the pregnancy (the thickness of the trophoblast, uterine flow, and expression of transporters) [54].
- An integrated pregnant sheep model that enables a direct administration and monitoring of xenobiotics over time in both fetal and maternal blood [55]. Because of important physiological similarities between the sheep and human placental functions, this model has contributed to significant advances in prenatal human medicine [56], despite the interspecies differences in the placental structure (the synepitheliochorial versus hemochorial placental structure) and transporters [56,57].

The ex vivo placental perfusion model had already been used to quantify the materno-fetal placental transfer of fifteen bisphenols [58]. Despite their chemical–structural similarities, these bisphenols differed greatly in the efficiency of placental transport. The placental transfer rates of bisphenol analogs such as 4,4′-Dihydroxybiphenyl, bisphenol AP (BPAP), bisphenol E (BPE), bisphenol F (BPF), 3-3BPA, bisphenol B (BPB), and BPA were similar to those of antipyrine, a molecule that passes the placental barrier by passive diffusion. This suggests that the exchange of these bisphenols across the placenta primarily involves passive diffusion and is limited only by the rate of the placental blood flow. In contrast, the placental transfer rates of bisphenol FL (BPFL) and BPS were very limited and intermediate for bisphenol BP (BPBP), bisphenol Z (BPZ), bisphenol C (BPC), bisphenol M (BPM), bisphenol P (BPP), and bisphenol AF (BPAF). This suggests a low diffusional permeability and/or that their passage might involve efflux transport. Moreover, although the glucuronide form of bisphenols could be detected in vivo in the fetal compartment, the placental transfer of glucuronide, evaluated for BPA and BPS, was almost non-existent. This indicates that, in the fetal compartment, these glucuronides come mainly from the fetoplacental metabolism, at least at the end of gestation [59,60]. A classical QSAR model based on molecular descriptors of BPs was developed to predict their materno-fetal transfer. But even if the physicochemical, topological, thermodynamic, and electronic parameters were able to influence the placental passage of this family of emerging BPs, the placental transfer efficiency of these 15 structurally related BPs could not be predicted solely from their physicochemical properties determined in silico [61]. This reinforces the indisputability of the physiological models.

Using the chronically catheterized fetal sheep model, toxicokinetic studies have been performed to determine the relative contributions of the placental transfer and the fetal and maternal metabolism pathways that control fetal exposure to BPA and BPS [55,60,62]. At the end of pregnancy, about 6% of the maternal dose of BPA reaches the fetal circulation, i.e., a dose related to the bodyweight equivalent to the maternal dose. This value was relative to the estimated 3.1% in humans using an ex vivo placental perfusion model [59]. Most of the BPA entering the fetal circulation was rapidly eliminated, mainly through the direct transfer of the BPA from the fetus to the mother (74%), and, to a lesser extent, through the fetal metabolism of BPA to BPA-glucuronide. In the fetus, entrapped BPA-glucuronide is eliminated by hydrolysis into the active form of BPA [62]. Therefore, the fetus has a much higher and sustained exposure to BPA metabolites.

It was striking that the fraction of the maternal BPS dose transferred from the mother to the fetus (0.40%) was about ten times lower than that of BPA [62]. This result is in agreement with the 10 times higher transfer efficiency of BPA from the mother to the fetus compared to that of BPS, as demonstrated in the ex vivo placental perfusion model [60]. Once in the fetal compartment, the percentage of the fetal dose of BPS entering the fetal blood eliminated by placental transfer was 26% compared to 74% for BPA. About half of the remaining BPS was metabolized to BPS-glucuronide by the fetus. The removal of BPS-glucuronide from the fetal compartment required its retro-conversion to bioactive BPS, like that of most bisphenol-glucuronides, due to the limited placental transfer. This toxicokinetic model predicted that, despite a lower materno-fetal passage of BPS compared to BPA, the higher persistence of BPS in the fetal compartment leads to expected BPS concentrations in fetal plasma of the same order of magnitude as that of BPA [63]. In agreement with these findings, the comparison of the fetal disposition of BPA, BPS, and BPF in the same pregnant ewe showed that the ratio of total feto-maternal concentrations of BPS was lower than those of BPF and BPA after a single subcutaneous administration of BPS or a mixture of BPA, BPS, and BPF [64].

To our knowledge, the placental transfer rate of TCS has not been studied using an ex vivo placental perfusion model or a pregnant sheep model. Concerning parabens, using a dual-recirculation placental perfusion model on term human placentae, Andersen et al. showed that the four parabens, MeP, EtP, PrP, and n-BuP, and their metabolite PHBA, just like BPA, are rapidly transported across the placental barrier. This implies potential fetal exposure [65].

Phthalate diesters are rapidly hydrolyzed to phthalate monoesters after maternal ingestion, inhalation, or dermal exposure in humans. In utero exposure to phthalates has been demonstrated in laboratory animals by measuring the concentrations of phthalate diesters or their metabolites in amniotic fluid [66], fetal plasma [66], and embryonic tissues [67]. In pregnant rats, the materno-fetal transfer of ^14^C-di(2-ethylhexyl)phthalate (DEHP) and ^14^C-diethyl phthalate (DEP) has been evaluated. This study showed that both phthalates and/or their metabolites were present in the placenta, amniotic fluid, and fetus [68]. Additionally, the pharmacokinetics of DBP in this animal model indicated that MBP was the major metabolite in the maternal and fetal plasma. This suggests that the active metabolite of DBP may cross the placenta in the late pregnancy. In amniotic fluid, the major metabolite was initially MBP, but by 24 h after administration, it was MBP-glucuronide [66]. Other studies confirmed that MBP was rapidly transferred to the embryonic tissues and that fetal plasma MBP-glucuronide concentrations were higher than those of the mother [67,69]. After the oral administration of DEHP to pregnant rats, unconjugated MEHP was the predominant metabolite measured in the amniotic fluid [70]. The transplacental transfer of MMP and MEHP, corresponding to the metabolites of DMP and DEHP, respectively, was evaluated in a dual-recirculation placental perfusion model of human placenta. This evaluation showed that MMP can cross the placenta by slow transfer, whereas no placental transfer was evidenced for MEHP. Moreover, MEHP, MBP, and MEP were found in fetal perfusate, corresponding to umbilical cord blood, whereas MMP, MEHHP, MBzP, and MiNP were undetectable. MEP, MBP, and MEHP were detected in placental tissue [71,72].

Altogether, these data demonstrated that the PPPs and their metabolites can cross the placenta, reach the fetal bloodstream, and contribute to the fetal exposome. Due to the short half-life of phthalate metabolites and the variation in the sensibility of the assay, the spot blood materno-fetal concentrations in term are difficult to interpret and cannot allow for the establishment of a relationship between the maternal and fetal concentrations, as shown for BPA [73,74]. Moreover, in the case of phthalates, it is not recommended to measure phthalates in the blood, since there are enzymes that can transform the parent into metabolites after blood collection, leading to false estimation.

## 5. Effects of PPPs on Human Placenta from Epidemiological Data and In Vitro Models and on Fetoplacental Growth from Animal Models

Only a few studies have looked at the association between PPPs and the markers of placental development. These are presented in the following paragraphs.

### 5.1. Epidemiological Data (Table 1)

Of the two studies investigating BPA exposure, one reported sex-specific associations (decreased female placental weight and increased placental weight in males, N = 232/220 male/female) [75], while the other, looking only at male births, showed no association with the placental weight nor with the placental-to-birthweight ratio, a marker of placental efficiency (N = 473) [36]. The single study looking at BPS did not report an association. This could be the result of the small sample size (N = 91) and the low frequency of detection of BPS in the urine (27%), thus limiting the power of the study [76]. Both studies on TCS reported negative associations with the placental weight; however, only one study reported it in male births [36], while the other study only reported it in female births [76]. Of the three studies investigating parabens (individual compounds or the molar sum), two studies reported a positive association with the placental weight in males at birth, either with n-BuP alone [76] or with the molar sum of the four parabens (including n-BuP) [36]. A third study evaluating paraben concentrations in placental tissues instead of maternal urine found opposite results (decreased placental weight with increased paraben placental concentrations) [77]. Of the two studies evaluating BP-3, only one study reported a positive association in male births [36], but the other study, perhaps because of its relatively small sample size (N = 91), did not report a positive association [76].

Regarding phthalates, a large cohort study (N = 2725 mother–child pairs from China) reported that, out of seven metabolites (MMP, MEP, MBP, MBzP, MEHP, MEOHP, and MEHHP), five (MMP, MBP, MEOHP, MEHHP, and MEHP) were associated with placental growth measurements, including the placental thickness, breadth, and length [78]. Most of these associations were positive, suggesting that prenatal exposure to phthalates can lead to a thicker and more circular placenta [78]. This cohort study reported stronger associations among male fetuses than in females. Moreover, the same cohort reported associations between all metabolites (N = 6: MMP, MEP, MBP, MEHP, MEOHP, and MEHHP), either individually or studied as the molar sum, and the placental weight [79]. The associations were also generally positive, particularly when exposure occurred late in pregnancy (the second and third trimesters). This cohort study also explored the effects of mixing, and although most phthalates were associated with placental outcomes when taken individually, no mixing effect was detected.

A few other studies with smaller sample sizes (ranging from 132 to 488) have also reported associations between certain phthalate metabolites and placental weight or the placental-to-birthweight ratio (PFR) [36,75,80]; however, the incriminated metabolites often differed from one study to another. Casas et al. reported a sex-specific association for MBzP that was positively associated with placental weight in boys and negatively associated in girls [75]. No association was reported for this metabolite in the other studies. Philippat et al. reported a negative association between cx-MiNP and MCOP and the placental weight or PFR in male fetuses [36], whereas this association was not reported in another study with a smaller sample size (N = 132, no stratification for child sex), which instead reported a decreased placental weight with prenatal exposure to MEP [80]. Interestingly, this study also reported a negative association between the preconception paternal exposure to DEHP and placental weight, and between the preconception maternal exposure to MEP and the birthweight-to-placental-weight ratio, providing preliminary results indicating the effect of the preconception exposure on placental development. Finally, a study evaluating phthalate metabolites in cord blood reported no association with the placental weight and volume [81].

Using the French mother–child cohort SEPAGES, 4 parabens, 2 bisphenols, 13 phthalate metabolites, and 2 non-phthalate plasticizer metabolites of DiNCH were measured in pools of repeated urine samples collected during the second and third trimesters of pregnancy. The results suggested negative associations between individual phthalate metabolites and the placental weight (MBzP and ΣDiNP), placental efficiency (MBzP, MBP, and ΣDiNP), and placental vascular resistance (MBzP, MBP, and ΣDiNP), indicating the adverse impacts of phthalate exposure on placental health [82]. Furthermore, using the same cohort, monoisobutyl phthalate (MiBP) and mono-n-butyl phthalate (MBP) were positively associated with most fetal growth parameters measured in the second trimester. Then, in the third trimester, MBP was further positively associated with the biparietal diameter and femur length [83].

**Table 1:** Effects of phenols, parabens, and phthalates on the placenta using epidemiological data.

Population	Placental Outcomes	Studied Compounds	Sample Type, Number, and Timing	Main Results				References
N = 2723 women–child pairs Recruitment: May 2013–September 2014 Country: China	Placental weight (calculated), PFR, chorionic plate area, disk eccentricity	MMP, MEP, MBP, ∑LMWP, MEHP, MEOHP, MEHHP, ∑DEHP	One spot urine sample per trimester	GM of three trimesters/T1/T2/T3 Placental weight: MMP ↑*/↓*/↑*/↑* MEP ↓*/↑*/↓*/↓* MBP ↑*/↑*/↑*/↑ ∑LMWP ↑*/↑*/↑*/↑* MEHP ↑*/↓*/↑*/↑* MEOHP ↑*/↑*/↑*/↑* MEHHP ↑*/↓*/↑*/↑* ∑DEHP ↑*/↓*/↑*/↑* Boys/girls stratification (on GM of three trimesters) Boys/girls Placental weight: MMP ↑*/↑* MEP ↑*/↓* MBP ↑*/↑* LMWP ↑*/↑* MEHP ↑*/↑* MEOHP ↓*/↑* MEHHP ↑*/↑* DEHP ↑*/↑*		PFR: MMP ↑*/↓*/↑*/↑ MEP ↓/↑/↓/↑ MBP ↑*/↑/↑*/↑* ∑LMWP ↑*/↑/↑*/↑* MEHP ↑*/↓*/↓/↑* MEOHP ↑/↑*/↑*/↑* MEHHP ↑*/↓*/↑*/↑* ∑DEHP ↑*/↓*/↑*/↑* PFR: MMP ↑*/↑* MEP ↓*/↑* MBP ↑*/↑* LMWP ↑*/↑* MEHP ↑*/↑* MEOHP ↑*/↑* MEHHP ↑*/↑* DEHP ↑*/↑*		[79]
N = 2725 (1399 boys and 1326 girls) mother–child pairs Recruitment: May 2013 and September 2014 Country: China	Placental length, thickness, breadth and surface area	MMP, MEP, MBzP, MEHP, MEHHP, MEOHP	One maternal spot urine sample per trimester	T1/T2/T3 Only chemicals with at least one significant results are shown Overall Placental breadth: MBP ↑*/↓/↑* LMWP ↑*/↓/↓ Placental thickness: MMP ↓/↑*/↓ MBP ↓/↑*/↑* MEOHP ↑/↑*/↑ MEHHP ↓/↑*/↑ LMWP ↓/↑*/↑ HMWP ↓/↑*/↑	Boys Placental breadth: MBP ↑*/↓/↑* LMWP ↑*/↓/↓ Placental thickness: MMP ↓/↑*/↑ MBP ↑*/↑/↑* MEOHP ↓/↑*/↑ MEHHP ↓/↑*/↑* LMWP ↓/↑*/↑* HWMP ↓/↑*/↑ MEHP ↓/↑*/↑*		GirlsPlacental breadth:MMP ↑*/↑/↑MBP ↑*/↑/↓LMWP ↑*/↑/↑Placental thickness:MBP ↑*/↑/↑*MEOHP ↑*/↑/↓	[84]
N = 473 mother–infant (only boys) pairs Recruitement: April 2003–March 2006 Country: France	Placental weight, PFR	MCPP, MBP, MiBP, MBzP, MEP, cx-MiNP, MCOP, MEHP, MEHHP, MEOHP, MECPP, 2,4-DCP, 2,5-DCP, n-BuP, BP-3, BPA, EtP, MeP, PrP, TCS	One maternal spot urine sample collected between 23 and 29 gestational weeks	Penalized effects: BP3 ↑ Placental weight TCS ↓ Placental weight ∑PB ↑ Placental weight MCOP ↓ PFR cx-MiNP ↓ Placental weight/↓ PFR		Unpenalized effects: BP3 ↑ Birthweight/↑ Placental weight TCS ↓ Placental weight ∑PB ↑ Placental weight cx-MiNP ↓ Placental weight/↓ PFR MCOP ↓ PFR		[36]
132 mother–child pairs Subfertile population Recruitement: 2005–2006 Country: USA	Placental weight assessed at birth	MEP, MBP, MiBP, MBzP, MEHP, MEHHP, MEOHP, MECPP, MCPP, MCOP, cx-MiNP	Paternal and maternal spot urine samples collected: (1) at recruitment (mother and father); (2) at each fertility treatment cycle (two spots for the mother and one for the father); (3) one spot per trimester of the pregnancy (mother)	[*p*-value] MEP ↑ Placental weight [0.80]/↓ FPR [0.02]				[80]
N = 207 women–child pairs, recruited between October 2011 and September 2012 Chongqing (Southwest China) Recruitement: October 2011–September 2012 Country: China	Placental weight at birth	DMP, DEP, DMEP, DBP, DEEP, DiBP, DPP, BMPP, DBEP, DCHP, DnHP, BBP, DEHP, DnOP, DNP	Cord blood sample	No effect on placental weight or volume				[81]
N = 142 mother–newborn pairs Recruitement: December 2014–December 2016 Country: Belgium	Placental weight at birth	MeP, EtP, PrP, n-BuP	Placenta sample	[*p*-value] In both sexes: EtP ↓ Placental weight [0.11] ∑Parabens ↓ Placental weight [0.08]	In boys: EtP ↓ Placental weight [0.24] ∑Parabens ↓ Placental weight [0.23]		In girls:EtP ↓ Placental weight [0.02] ∑ Parabens ↓ Placental weight [0.03]	[77]
N = 657 mother–child pairs Recruitment: 2004–2006 Country: Spain	Placental weight at birth	BPA, DEHP (∑ of MEHP, MEHHP, MEOHP, MECPP), MBzP, LMWP (∑ of MEP, MiBP, MBP	Two spot urine samples collected at 12 ± 1.7 and 32 ± 1.4 weeks of gestation	In boys: MBZP ↑ Placental weight		In girls: MBZP ↓ Placental weight		[75]
N = 130 mother–child pairs Recruitment: 2006–2008 Country: USA	Placental weight, fetal weight, head circumference, abdominal circumference, and femur length	2,4-DCP, 2,5-DCP, BP-3, n-BuP, EtP, MeP, PrP, TCS, BPS, TRCB	One spot urine sample	Inverse associations were observed between average 2,4- and 2,5-DCP concentrations and birth weight z-scores in males Inverse associations between average TCS exposure over pregnancy and estimated fetal weight combined with birth weight in repeated measures models in males				[76]
SEPAGES cohort N = 484 pregnant women Recruitment: 2014–2017 Country: France	Placental weight, PFR, placental thickness, and placental vascular resistance	4 parabens, 2 bisphenols, triclosan, benzophenone-3, 13 phthalate metabolites, and 2 non-phthalate plasticizer metabolites	Repeated urine samples collected during the second and third trimesters of pregnancy	Several phthalate metabolites were negatively associated with placental outcomes MBzP: ↓ placental weight and PFR (T2 and T3 trimesters) Negative associations with placental weight and PFR for males only (T3 trimester) MBP: ↓ placental vascular resistance (T2 and T3 trimesters) Σ DiNP: ↓ placental vascular resistance (T3 trimester), ↓ placental weight and PFR in males only No associations between phenols and placental outcomes				[82]
SEPAGES cohort N = 484 pregnant women Recruitment: 2014–2017 Country: France	Fetal biparietal diameter, femur length, head and abdominal circumferences measured by ultrasound, newborn weight, length, and head circumference measured at birth	13 phthalates, and 1,2-cyclohexane dicarboxylic acid and diisononyl ester (DiNCH) metabolite	Repeated urine samples collected during the second and third trimesters of pregnancy	MiBP: ↑ biparietal diameter, and head and abdominal circumferences at T2 trimester MBP: ↑ estimate fetal weight, and head and abdominal circumferences in males Mixture of phthalate/DiNCH metabolites: ↑estimate fetal weight (T3 trimester)				[83]

BBP: benzyl butyl phthalate; BMPP: bis (4-methyl-2-pentyl) phthalate; n-BuP: n-butylparaben; BPA: bisphenol A; BP-3: benzophenone-3; DBP: di-n-butyl phthalate; DBEP: bis (2-nbutoxyethyl) phthalate; DCHP: dicyclohexyl phthalate; 2,4-DCP: 2,4-dichlorophenol; 2,5-DCP: 5-dichlorophenol; DEEP: bis (2-ethoxyethyl) phthalate; DEHP: di-(2-ethylhexyl) phthalate; DEP: diethyl phthalate; DnHP: di-n-hexyl phthalate; DiBP: diisobutyl phthalate; DiNCH: di-iso-nonyl-cyclohexane-1,2-dicarboxylate; DMP: di-methyl phthalate; DMEP: bis (2-methoxyethyl) phthalate; DnOP: di-n-octyl phthalate; DNP: dinonyl phthalate; DPP: di-amyl phthalate; EtP: ethylparaben; HMW: high-molecular-weight phthalates; LMW: low-molecular-weight phthalates; MBP: mono-n-butyl phthalate; MBzP: monobenzyl phthalate; cx-MiNP: mono-carboxy-iso-nonyl phthalate; MCOP: mono-carboxy-iso-octyl phthalate; MCPP: mono-3-carboxypropyl phthalate; MECPP: mono-2-ethyl-5-carboxypentyl terephthalate; MEHHP: mono-2-ethyl-5-hydroxyhexyl) phthalate; MEHP: mono-2-ethyl-hexyl) phthalate; MEOHP: mono(2-ethyl-5-oxohexyl) phthalate; MEP: mono-ethyl phthalate; MiBP: mono-iso-butyl phthalate; MMP: mono-methyl phthalate; MeP: methylparaben; PFR: placental-to-birthweight ratio; PrP: propylparaben; TCS: triclosan; TRCB: triclocarban. * indicates statistically significant results, ↓ indicates a decrease, ↑ indicates an increase.

### 5.2. Effects of PPPs on Human Placental Methylation

DNA methylation is the best characterized and most stable epigenetic modification, influencing the chromatin structure and the gene expression. This epigenetic mark typically involves the methylation of the fifth carbon position at a cytosine residue within a CpG dinucleotide (CpG), resulting in 5-methylcytosine (5mC). Recent studies have provided concrete evidence of a link between DNA methylation alterations and certain environmental exposures. Given the evidence that PPPs can cross the placenta, studies have explored the associations between PPP maternal exposure and the evolution of DNA methylation profiles in the placenta. The first studies focused on the locus of imprinted genes, *IGF2* and *H19* [85]. This locus displays an allele-specific expression (the expression of *H19* from the maternal allele and of *IGF2* from the paternal allele). The *IGF2* gene and lncRNA *H19* are together important for embryogenesis and fetoplacental development. The allele-specific methylation is involved in the allele-specific control of expression. Using placental samples collected at delivery from 196 healthy newborns, genomic DNA was used to analyze three differentially methylated regions, IGF2DMR0, IGFDMR2, and H19DMR, considered as imprinting the center region (*H19 ICR*). An increase in the level of methylation of the imprinting center region (*H19 ICR*) was associated with the sum of phthalate metabolites and low-molecular-weight metabolites. An increase in high-molecular-weight phthalate metabolites and DHEP exposure was associated with a deviation of the allele-specific expression of *H19* (10%) only in male placenta. This suggests a potentially sexually dimorphic response. Conversely, the study showed that the sum of phthalate metabolites and high-molecular-weight metabolites was associated with a decrease in *IGF2DMR0*, without significant modification of the methylation level of *IGF2DMR.* In this study, the effects of the exposure to phenols were also investigated, but no significant association was found [85]. Quite different results were obtained by Zhao et al. [86] from the placental samples of 101 healthy newborns and 80 neonates with fetal growth retardation. The placental methylation of *IGF2DMR* was significantly inversely associated with MEHHP and MEOHP concentrations. Associations were much more evident in neonates with fetal growth restriction than in healthy neonates. Using the same mother–newborn cohort, measuring the phthalate exposure during the third trimester, the same authors demonstrated that the placental methylation of LINE-1 was negatively correlated with phthalate exposure levels. This outcome was significantly altered in cases of fetal growth restriction compared to healthy births. Because the LINE-1 repetitive element exhibits a wide distribution in the human genome, it has been frequently used as a surrogate marker of global methylation analysis. The modest hypomethylation of the LINE-1 repetitive element observed after exposure to phthalates could contribute to genome instability and increase the risk of chronic disease later in life [87].

A genome-wide DNA methylation analysis using the Illumina Infinium Human Methylation 450K or 850K Bead Chip was also performed. Although these differently methylated CpG sites constitute a small proportion of the total number of CpG sites in the genome (28 million), they represent a wide distribution of sites [88]. Grindler et al. used this tool to analyze genome-wide DNA methylation marks in placental villi in the first trimester from 16 women with high or low phthalate exposures [89]. They reported 2214 differentially methylated cytosines (DMCs) targeting 1460 unique genes. Taking into account the proximity of the DMCs, 282 differentially methylated regions (DMRs) were identified and found to be associated with 245 unique genes. Interestingly, using the same placental samples for the transcriptomic analysis, the authors identified 163 differentially expressed genes (DEGs) among the targeted genes by DMCs: 124 were downregulated and 39 were upregulated between women with a high-level exposure of phthalates compared to those with a low-level exposure. The authors focused on one particular pathway, erbB signaling. EGFR exhibited placental hypermethylation and decreased expression in women, with a high total phthalate exposure, suggesting that this gene might be the specific target of endocrine disruption by phthalates. Alterations in the erbB signaling methylome have previously been observed in adverse obstetric outcomes such as pre-eclampsia and IUGR.

In two recently published studies [90,91] based on a larger cohort of mother–infant pairs (the French EDEN cohort, N = 202), placental DNA methylation at birth was measured using the Illumina Infinium Human Methylation 450K Bead Chip in combination with 12 urinary phthalate metabolites [91] and 9 phenols [90]. In these studies, only placental samples from boys were assessed for DNA methylation. Since biological functions are in general more strongly associated with genomic regions than single CpGs [92], these two studies aimed to identify DMRs associated with phenol and phthalate concentrations. Most of the associations observed were positive (i.e., increased methylation with increased exposure during pregnancy). TCS exposure was positively associated with 37 DMRs compared to less than 6 for the other phenols and phthalate metabolites studied. Out of the 37 DMRs associated with TCS, 6 encompassed imprinted genes, which represented a significant enrichment. Using the SEPAGES cohort (N = 387 mother–child couples), an exploratory analysis on individual CpGs and DMRs, as well as 20 previously identified CpGs, was performed. In the sex-stratified analysis, 114 individual CpGs (68 in males and 46 in females) were differentially methylated, encompassing 74 genes (36 for males and 38 for females). There was a total of 82 significant DMRs (40 for females and 42 for males). For most DMRs, DNA methylation levels increased with a higher exposure, except for some parabens and DiNP metabolites in males and females, BPA in males, and BPS and DiNCH metabolites in females, which were negatively associated with DNA methylation in most DMRs. Some differentially methylated CpGs and DMRs encompassed imprinted genes, whereas other CpGs were linked to adiposity, the lipid and glucose metabolism, and cardiovascular function [93]. Recently, maternal concentrations of monocarboxyisononyl phthalate (cx-MiNP), mono-3-carboxypropyl phthalate (MCPP), and BPA were associated with altered methylation in the placenta (the maternal or fetal side). Among them, MCPP was associated with differential CpG methylation [94].

Further studies are needed to better control the analysis conditions (the PPP exposure time, placental sampling, pregnancy stages, genotyping, etc.) and to acquire more causal information between the methylation landscape, gene expression, and placental dysfunction induced by EDCs.

### 5.3. Effects of PPPs on Human Placental Function Using In Vitro Models

In humans, trophoblasts differentiate into extravillous cytotrophoblasts that proliferate, invade the uterine wall, and are important for remodeling the endometrial vasculature and syncytiotrophoblasts. The latter cell type resulting from the fusion of cytotrophoblasts is responsible for the endocrine function of the placenta that produces steroid (progesterone) and polypeptide hormones (human chorionic gonadotropin, namely, hCG, and leptin). To study the effects of PPPs on the human placentation, in vitro studies could be performed using (i) primary cell cultures of trophoblasts from first- or third-trimester placentae [95,96,97,98,99], (ii) placental explants [100,101], and (iii) immortalized trophoblast cell lines such as BeWo, JEG-3, and HTR-8/SVneo [102,103,104,105]. The BeWo cell line can fuse and express differentiation markers [106,107,108,109], whereas JEG-3 cells can produce placenta-specific hormones, but without cell fusion [110,111]. The HTR-8/SVneo cell line is used as a model of extravillous trophoblasts [112,113,114].

Using human placenta, the exposure of first-trimester placental explants to BPA (0.5–1 nM) stimulated hCG secretion [115,116] and increased cell apoptosis [116]. The same effects were observed when term primary trophoblasts were exposed to BPA (8 nM–8 mM) [117,118]. Unlike the first-trimester explants, Zou et al. did not observe any change in the level of hCG secretion at any BPA concentration tested in term villous explants. Interestingly, they showed that BPA significantly increased ESRRG expression (estrogen-related receptor gamma) in the female placentae following exposure to 1 µM BPA for 24 h. This suggests that exposure to a low dose of BPA could alter gene expression in human placentae in a sex-specific manner [100]. Moreover, in primary trophoblast cells, data have shown that BPA is also able to (i) induce the expression of corticotrophin-releasing hormone (CRH) and the expression of two enzymes specifically involved in hormone production (aromatase and 11-β-hydroxysteroid dehydrogenase 2, known as 11β-HSD2), and, conversely, (ii) to reduce the expression of leptin obtained in human term primary trophoblasts [117]. Concerning BPS, this phenol has been shown to block epidermal growth factor (EGF)-mediated trophoblast fusion in term primary trophoblasts [118].

In the first-trimester trophoblast progenitor cells and second-trimester primary trophoblasts, a mixture of four phthalates (MBP, 0.2 µM; MBzP, 3 µM; MEHP, 0.7 µM; MEP, 1.5 µM) decreased hCG expression regardless of the fetal sex. However, the same mixture of phthalates decreased the placental peroxisome proliferator-activated receptors-γ (PPARγ) expression in male cells and increased female cells. This latter finding provides evidence for sex-specific responses to phthalates in human trophoblasts [98]. In primary-term trophoblasts, a recent study demonstrated that, at low concentrations (0.1–1 µM), MEHP decreases the lipid content, hCG secretion, and cell fusion. These effects appear to be associated with a lower activity of (PPARγ). This receptor is a key transcription factor involved in the control of trophoblast differentiation and the lipid metabolism. In contrast, a high concentration (10 µM) of MEHP increases the lipid content, cell fusion, and PPARγ activity, but decreases the hCG secretion. These results highlighted the notion of a non-monotonic dose–response, particularly at environmentally relevant levels of MEHP exposure [97]. Moreover, at this stage, high MEHP doses (100–150 µM) increase the expressions of CRH and cyclooxygenase 2 (COX2) via the nuclear factor κ-light-chain-enhancer of activated B cells (NF-κB) signaling pathway. These results suggest that MEHP could prematurely induce the expression of labor-promoting genes and lead to preterm birth [119].

Using the BeWo cell line, exposure to low doses of BPA (3 nM and 1 µM) has been shown to significantly increase cell proliferation [120,121], whereas exposure to a high dose of BPA (1000 µM) decreased the proliferation rate of cells [121,122]. However, in other studies, the exposure of BeWo cells to BPA did not affect the proliferation or metabolic activity [123]. BPA exposure has also been shown to induce cell cycle inhibition, increase DNA damage [122], and reduce the viability of BeWo cells at concentrations ranging from 100 µM to 1 mM [116]. Regarding β-hCG secretion, a significant decrease was observed at a 30 µM BPA exposure, while the secretion dropped dramatically from 60 to 125 µM BPA [116]. BPA is able to promote the syncytialization in BeWo cells at doses of 1 and 50 mM, known in humans to increase the expression of several syncytin proteins [123], as well as to reduce the invasive capacity of the same cell line at doses as low as 100 µM [124] due to the increased expression of E-cadherin. BPA was also able to reduce the activation of the antioxidant response element [121] and increase the levels of two anti-apoptotic proteins, i.e., B cell lymphoma 2 (Bcl-2) and heat shock protein 70 (Hsp70), and decrease the levels of hypoxia-inducible factor 1-α (HIF-1α) under stress conditions, demonstrating that BPA inhibits trophoblast cell death under cellular stress conditions [121]. Moreover, BPA might exert its various effects mainly by regulating cell–extracellular matrix interactions via transforming growth factor-β (TGF-β) signaling [125]. Cytotoxic effects via cell cycle arrest and apoptotic pathways were also observed in BeWo cells exposed to EtP [126].

As JEG-3 cannot fuse, there are few studies on this cell line. It has only been shown that, at doses of 5–20 µg/mL, BPF induces chromatin condensation [102,103,104]. Concerning MEHP exposure, the incubation of JEG-3 cells (10 mM for 72 h) exhibited a marked change in the lipid profile, especially in triacylglycerols and glycerophospholipids, with a marked accumulation of triacylglycerols. This once again underlines the detrimental effects of MEHP on the lipidome of human placental cells [127].

Using the HTR-8/SVneo cell line, a high dose of TCS exposure suppressed the viability and migration of HTR-8/SVneo cells but increased the H19 imprinted gene expression and insulin-like growth factor 2 (IGF2) protein secretion in a dose-specific manner. This suggests an altered trophoblast function, gene expression, and DNA methylation status [112]. Regarding the exposure to the paraben family, n-BuP has also been shown to inhibit cell proliferation and induce apoptosis, which could disrupt early placental development [128]. Regarding phthalate exposure, high concentrations of MEHP for 24 h significantly inhibited cell proliferation and viability, promoted apoptosis, and inhibited the cell cycle [129]. In addition, DEHP acts on placental trophoblast cells and inhibits its internalization of transthyretin (thyroxine transport protein), downregulates transthyretin expression, and affects the expression of deiodinase 2, deiodinase 3, and thyroid hormone receptor in the HTR-8/SVneo cell line, as well as in JEG-3 cells [130]. These data suggest that DEHP disrupts the placental transport of thyroid hormones, which could be very detrimental to fetal development, especially at early stages.

In conclusion, depending on the phenol concentrations, BPA may or may not affect placental cell proliferation and hormone production. Parabens, on the other hand, induce cytotoxic effects and apoptosis. Depending on their concentration, phthalates affect the lipid content, hCG secretion, and cell fusion by PPARγ. All of these cell models are useful for testing several concentrations of PPPs and deciphering their mechanism of action. However, these approaches do not allow us to take into account the sub-chronic effects, nor the complexity of the different cell types of the placenta. It is, therefore, essential to supplement the in vitro data with data from in vivo models.

### 5.4. Effects of Phenols and Parabens on Fetoplacental and Neonatal Outcomes Using Animal Models (Table 2)

As a very detailed review of phthalate exposures and placental health has been published by Seymore et al. in 2022 [131], an update has been made over the last two years. Exposure to 20 µg/kg BW/day of DiNP on days 1–7 of gestation in mice led to fetal loss towards the end of gestation. This miscarriage was due to the impaired differentiation of stromal cells associated with a defect in the angiogenic network in the decidua, but also to a disorganization of the placental layers. Interestingly, the labyrinthine area involved in nutrient exchanges was reduced [132].

In rats, oral exposure to BPA (0.0025, 0.025, and 0.250 mg/kg/day) 30 days before pregnancy and during the first 20 days of pregnancy reduced the placental weight at low and high BPA concentrations, while the fetal weight was increased only at low concentrations [133]. In contrast, gestational exposure to BPA in ewes reduced the placental efficiency and fetal weight at mid-gestation [134] and at 110 days [135]. Similarly, the exposure of pregnant mice to BPA at 0.050 mg/kg/day by oral gavage from day 1 to 7 of gestation induced fetal growth restriction at 14 days of gestation, which was preceded by the insufficient remodeling of uterine spiral arteries [136]. In addition, a significant decrease in the number of embryos and the weight of the uterus on days 10 and 12 was reported after the subcutaneous administration of BPA (10 mg/kg/day) to pregnant mice from day 1 of gestation to day 7 [137].

The study of the placental structure was analyzed after exposure to BPA. Tait et al. exposed pregnant mice to BPA at 0.5 mg/kg bw (corresponding to the lowest observed adverse effect level, LOAEL) or 50 mg/kg bw (corresponding to a high range of human BPA exposure) from GD1 to GD11. They demonstrated that a high concentration of BPA induces a significant degeneration and necrosis of giant cells, associated with an increase in vacuolation in the junctional zone and a reduction in the spongiotrophoblast layer at GD12 [138]. Degenerative changes were also found in the trophoblastic giant cells and spongiotrophoblast layer in the pregnant mice exposed to BPA (10 mg/kg/day, from day 1 to day 7) by subcutaneous administration [137]. A low concentration of BPA (0.5 mg/kg bw) induced the development and branching of blood vessels, while a high concentration of BPA inhibited them. Maternal vessels were narrower in placentae exposed to a low concentration of BPA, whereas embryonic and maternal vessels were irregularly dilated in the labyrinthine area of placentae exposed to a high concentration [138]. Doses of 10 and 40 mg/kg BPA (mice exposed from GD0.5 to GD5.5) also decreased the proportion of the labyrinthine and spongiotrophoblast layers, which are associated with large vacuoles [139]. An increase in the retention of smooth muscle cells and a decrease in vascular surfaces at the level of the junctional zone have been reported in mice exposed to BPA at a concentration of 0 to 400 µM in drinking water from GD7 to GDl7 [140]. Altogether, these data indicate that BPA has an effect on the placental structure, which explains the disturbance to the fetal weight.

BPA-related gene expression changes have also been studied. Since BPA is thought to bind to estrogen receptors and regulate the expression of estrogen-responsive genes, BPA may alter the expression of other placental nuclear receptors such as the retinoid Z receptor (RORγ), progesterone receptor (PR), ERβ, LXRα, germ cell nuclear factor (GCNF), steroidogenic factor-1 (SF-1), and so on. The oral administration of BPA at a dose of 0.002 mg/kg/day from GD6.5 to GD17.5 induced the sex-specific placental differences in these genes at GD18.5 [141].

Post-translational modifications of histones, DNA methylation, and the expression of imprinted genes have been studied in mouse placenta concerning BPA exposure. Daily exposure of pregnant mice to BPA between GD8.5 and GD12.5 affected the expression of imprinted genes. At GD13.5, *Rtl1* displayed a slight disturbance of allele-specific expression [142]. A longer maternal exposure time to BPA (two weeks before mating and during gestation) significantly disrupted the expression of imprinted genes in the placenta at GD12.5, such as *Snrpn* and *Kcnq1*, and altered the methylation levels of differentially methylated regions (DMRs), including the *Snrpn* imprinting control region (ICR) and *Igf2* DMR1 [143] Moreover, exposure significantly reduced the genome-wide methylation levels in the placenta, but not in the embryo. The expression of several small RNAs was also found to be disturbed [144]. These epigenetic defects were associated with abnormal placental development [143]. The exposure of pregnant mice from GD7 to GDl7 to BPA at a concentration of 0 to 400 µM in drinking water disrupted the expression of 10 genes coding the proteins of epigenetic machinery. These genes include enzymes involved in histone methylation and acetylation, protein phosphorylation, and DNA methylation. Among these enzymes, the DNMT1 mRNA/protein was increased in the placenta, while levels of DNMT3a and -3b, hydroxymethyl transferases (TET1, -2, and -3), and 5-hmc were unaffected. Moreover, high levels of 5-mc indicated an elevated level of methylation in the placental tissues of mice exposed to BPA [140].

As previously described, BPA altered the placental structure, leading to impaired placentation. This could result from increased protein levels of matrix metalloproteinase-9 and 2 (MMP-9 and MMP-2) and decreased levels of the tissue inhibitor of metalloproteases-3 (TIMP-3), as well as integrin-β1 and integrin-α5, in mouse placenta. These effects were observed in particular in the labyrinthine layer in the event of a low exposure to BPA (5, 10, and 40 mg/kg) from GD0.5 to GD5.5 [139]. Ye et al. confirmed that placental abnormalities were associated with altered invasion-related genes, such as increased tissue inhibitors of metalloproteinases, decreased metalloproteinases, and the Wnt family member WJVT2/β-catenin (mice exposed from GD7 to GDl7 to BPA added at a concentration of 0–400 µM in drinking water) [140]. BPA affected several placental transporters, such as cation transport channels. For example, in mice placenta at GD17.5, PMCA1 (ATPase, Ca^++^ transporting, plasma membrane 1), hephestin (HEPH), CTR1 (solute carrier family 31, member 1 (copper transporter)), and ATP7A (ATPase, Cu^2+^ transporting, alpha polypeptide) were disturbed after exposure to BPA (50 mg/kg/day) from GD11.5 to GD16.5. These disturbances were associated with a decrease in serum calcium/copper/iron levels, which could have an impact on fetal development [145]. Exposure to high doses of BPA (400 and 600 mg/kg) from GD17 to GD19 increased CaBP-9k mRNA/protein in the maternal uterus and placenta in late gestation. CaBP-9k is a vitamin D-dependent calcium-binding protein whose gene carries an estrogen response element (ERE) [146,147]. In rats fed a diet containing BPA (0.0025, 0.025, or 0.250 mg/Kg/day) for a month, plus 20 days during pregnancy, the glucose type 1 transporter was upregulated [148]. BPA modulation of cation transport channels, CaBP, and placental nutrient-glucose transfer could explain the changes in the fetal weight.

As exposure to BPA in pregnant mice (2, 20, and 200 mg/kg bodyweight/day from GD13 to GD16, and euthanized at GD17) increased the plasma estrogen, testosterone, and CRH in dams, the regulation of CRH involved in fetal organ development, the glucose metabolism, and immune response has been investigated in the placenta. The activation of phosphorylated forms of PKC ζ/λ and δ might promote cAMP-responsive element-binding protein (CREB) phosphorylation. This leads to its interaction with a CBP-responsive element (CRE) located in the CRH gene promoter and to an increase in CRH mRNA, which might be the pathway for the signaling of preterm birth [149].

Other mediators differ between mid- and early gestation in terms of protein expression in the BPA-exposed placenta. Exposure to BPA in ewes at the start of gestation increased interleukin 8, the marker of lipid peroxidation, antioxidants, aromatase, 17 alpha-hydroxylase, estrogen receptor 2, IGF-2 receptor and IGF-binding proteins (IGFBPs), and histone deacetylase 1 and 2, and caused a reduction in tumor necrosis factor-alpha and the IGF1 receptor. Whereas, at mid-gestation, BPA exposure reduced angiogenic factor hypoxia-inducible factor 1 alpha, but increased IL1beta, oxidative stress markers, triglyceride, 17-alpha hydroxylase, IGFBP 1, DNA methyltransferase 3 A, and histone deacetylase 1, which could explain the low birth weight [134]. Exposure to BPA from day 40 through day 110 of gestation in ewes (5 mg/kg/d) was responsible for the placental cytotoxicity, including autophagy, apoptosis, endoplasmic reticulum stress, excessive ROS generation, oxidative damage, and mitochondrial dysfunction [135].

BPA is the most studied phenol so far, but other molecules of the same family are also studied, including BPS, TBBPA, and TCS. BPS is an analog of BPA that is reputedly more inert. Daily exposure to BPS from GD30 to 100 in pregnant ewes reduced maternal circulating pregnancy-associated glycoproteins (PAG1 and PSPB) but did not change the placental weight or placental stereology [150]. However, exposure to BPS in mice (0.200 mg/kg bodyweight BPS or BPA 2 weeks before mating and until day 12.5 of gestation) was shown to reduce the ratio of the spongiotrophoblast zone to trophoblast giant cells within the junctional zone, as well as the exposure to BPA. In addition, BPA and BPS altered placental neurotransmitters such as serotonin and dopamine. BPA and BPS reduced placental serotonin (5-HT) concentrations and 5-HT giant cell immunoreactivity, whereas the concentrations of dopamine and 5-hydroxy indole acetic acid, the main metabolite of serotonin, increased, as well as the dopamine immunoreactivity of the giant cells. Due to BPA and BPS exposure, this imbalance associated with a decrease in docosahexaenoic acid and estradiol could affect the placental–brain axis of the mouse fetus. BPS exposure causes placental effects almost identical to those of BPA, which would justify considering BPS as being as dangerous as BPA [151]. Finally, placentae exposed to BPS in pregnant ewes (from day 30–100) showed a low expression of the protein E-cadherin, few binucleate cells, and a high expression of missing-1 protein in glial cells, suggesting that BPS can affect the trophoblast fusogenic signaling pathway and the placental endocrine function [150].

The administration of TCS at doses of 523 and 785 mg/kg/day on GD1 to GD3 has been observed to impair blastocyst implantation in mice [152]. In animal models, TCS was able to bioaccumulate in the placenta, liver, kidney, ovary, adrenal, spleen, and fat, but with high concentrations for the first four tissues [153,154]. Daily oral exposure of pregnant mice with 8 mg/kg of TCS, but not 1 or 4 mg/kg, from GD6 to GD18 resulted in a decreased fetal bodyweight and an increased rate of fetal loss (spontaneous abortions and fetal growth restriction) [155]. Similarly, a short exposure to TCS from GD5.5 to mid-gestation caused a dose-dependent increase in the rate of fetal loss through abortion [156]. Spontaneous abortion has also been reported in pregnant rats exposed to TCS by gavage with 600 mg/kg/d from GD6 to GD20 [154]. A high TCS concentration decreased the placental weight (exposure by gavage at doses of 1, 10, and 100 mg/kg/day from GD5.5 to GD15.5), and the placental structure was characterized by a thrombus, hemorrhage with tissue necrosis, and junctional zone atrophy [156]. Exposure to 8 mg/kg/day from GD6 to GD18 confirmed the reduction in placental weight and labyrinth volume [155]. Taken together, these data demonstrated that TCS is capable of inducing miscarriage and affecting the placental structure. In addition, TCS negatively modulated the activities and expression of placental System A amino acids or glucose transporters in pregnant mice exposed to 8 mg/kg/day. These negatively modulated activities were associated with a decrease in the sodium-coupled neutral amino acid transporters (SNAT1/SNAT4) and glucose transporter 1 (GLUT-1) mRNA, respectively [155]. These indicate an effect on nutrient exchanges that may contribute to fetal growth restriction.

Since TCS is an endocrine disruptor, the levels of reproductive hormones and thyroid hormones, and their enzymes, have also been studied. In pregnant ewes, as well as in mice, a high concentration of TCS decreased estrogen sulfonation [153]. Indeed, estrogen sulfotransferase activities, implicated in both estradiol and estrone sulfonation, were significantly reduced when pregnant mice were exposed to TCS. Although the serum estrogen concentration was normal, the ratio of sulfo-conjugated E2 and unconjugated E2 was reduced in mice exposed to TCS. Interestingly, the estrogen receptor antagonist, an estrogen sulfotransferase activity inhibitor, was able to rescue the platelet aggregation and placental thrombosis, and limit spontaneous abortion [156].

Feng et al. studied the impact of TCS exposure on placental steroid metabolism enzymes, including UDP-glucuronosyltransferase 1A1 (UGT1A1), estrogen sulfotransferase 1E1 (SULT1E1), and steroid 5α-reductase 2 (SRD5A2). This study indicated a significant impairment with high concentrations of TCS as well as progesterone and estrogen receptors. This could explain why placental hormones such as progesterone, estradiol, testosterone, and prolactin secreted in maternal blood were reduced in groups exposed to high doses of TCS [154]. T3 and T4 are known to affect fetal growth and development. In the event of exposure to a high concentration of TCS (8 mg/kg/day orally from GD6 to GD18), TCS induced hypothyroxinemia in pregnant mice. As thyroid hormones stimulate the Akt-mTOR-p70S6K and ERK signaling pathways, which can regulate the activation of placental amino acid transporters, these pathways have also been investigated. TCS decreased placental Akt, mTOR, and P70S6K phosphorylation, but this was corrected by L-thyroxinein (T4). In fact, T4 was able to rescue the activity and expression of amino acid and glucose transporters and decrease fetal bodyweight [155].

In pregnant rats, following the administration to dams of 100, 200, and 400 mg/kg bodyweight/day from GD7 to 21, parabens (EtP and n-BuP) were distributed in rat maternal plasma, pools of amniotic fluids, placenta, whole-body fetuses, and in the fetal liver. Additionally, high levels of EtP were found in all fluids and tissues compared to n-BuP [157].

All of these experiments relating to maternal oral exposure to PPPs and placental effects were always performed using one pollutant, variable doses, and different timings of exposure, which rarely covered the preconceptional and gestational periods.

**Table 2:** Effects of phenols, phthalates, and parabens on fetoplacental development according to the animal model, the chemicals, the dose administrated, the exposure route and duration.

Animal Model	Chemicals	Dose Administered	Exposure Route	Exposure Duration	Observation Stage	Function Studied	Fetoplacental Outcomes	Additional Outcomes	Reference
Mouse	BPA	0.002 mg/kg bw/d	Oral gavage	GD6.5-17.5	GD18.5	Placental function	Peroxisome proliferator-activated receptor alpha and gamma (*PPARα*, *PPARγ*) and Aryl hydrocarbon receptor (*AhR*) mRNA expression: no effect RAR-related orphan receptor gamma (*RORγ*) mRNA expression: BPA ↓ (♀) Estrogen receptor beta (*Erβ*) and *LXRα* mRNA expression: BPA ↑ (♂) Progesterone receptor (*PR*) mRNA expression: BPA ↑ (♂)/↓ (♀) Placental expression of 6 non-nuclear receptor protein mRNAs: BPA ↓ (♂)/↑ (♀)	*COUP-TFα*, *GCNF*, *SF-1*, and *PNR* mRNA expression: BPA ↓ (♂)/↑ (♀)	[141]
Mouse	BPA	0.05 mg/kg bw/d	Oral gavage	GD1-7	GD5, 8, 10, 12, 14	Fetoplacental development/placenta and uterine spiral artery structure	Placental areas, thickness, and diameter: no effect Placental weight: BPA ↓ (GD14) Fetal bodyweight: BPA ↓ (♀ GD14) Percentage of intrauterine growth restriction (IUGR): BPA ↑ (40.5% IUGR) Uterine spiral artery wall thickness: BPA ↑	Implantation number and abortion rates: no effect Implantation sizes: BPA ↓ (GD12) Uterine artery blood velocity values and uterine natural killer cell numbers: no effect Uterine spiral artery smooth muscle actin staining and wall-to-lumen ratio: BPA ↑	[136]
Mouse	BPA	0.5 or 50 mg/kg bw/d	Oral gavage	GD1-11	GD12	Fetoplacental development/placental structure	Placental diameter and embryo length: BPA ↑ (50) Right forelimbs length: BPA ↓ (0.5)/↑ (50) Yolk sac length, first somite longitudinal–transversal diameter, and first branchil arch longitudinal–transversal diameter ratios: BPA ↑ (0.5)/↓ (50) Trophoblast giant cell degeneration and necrosis: BPA ↑ Spongiotrophoblast layer: BPA ↓ (50) Maternal blood space area: BPA ↑ (50)/↓ (0.5) Displacement of β-catenin from membrane to nucleus in the spongiotrophoblast and the labyrinthine cells: BPA ↑ (0.5) Numbers of differentially expressed genes (DEGs): 582 (0.5)/701 (50) Numbers of significant gene clusters: BPA (0.5 (11)/50: (13)) Gene cluster involvement: vascular/blood vessel development, nucleotide binding, embryonic morphogenesis and proteolysis, peptidase activity, progesterone-mediated oocyte maturation, gap junction pathways, chromosome organization, and chordate embryonic development		[138]
Mouse	BPA	0, 2, 20, or 200 mg/kg bw/d	Oral gavage	GD13-16	GD17	Placental function	Placental *CRH* mRNA expression: BPA ↑ (200) Placental *CYP19* mRNA expression: no effect Placental cAMP-response element-binding protein (CREB) level: BPA ↑ Preterm delivery: BPA ↑	Plasma CRH, estradiol, and testosterone concentrations: BPA ↑ (20, 200) Protein kinase C ζ/λ phosphorylated forms: BPA ↑ Protein kinase C delta (PKCδ) phosphorylated forms: BPA ↑ (200)	[149]
Mouse	BPA	0, 0.4, 4, 40, or 400 µM BPA in drinking water	Orally (drinking water)	GD7-17	GD13, 16 and 17	Placental structure and function	Mean fetal and placental weight, litter size, and placental-to-bodyweight ratio: no effect Placental vessel area: BPA ↓ Trophoblast invasion-related gene expression: BPA ↑ (*TIMP1*, *TIMP2*)/↓ (*MMP2*, *MMP9*, *WNT2*) Number of differentially expressed genes related to placental epigenetic modifications: BPA ↑ (8)/↓ (2)	Dams systolic blood pressure: BPA ↑ (4, 40, 400) Dams glomerular atrophy: BPA ↑ Maternal bodyweight: no effect	[140]
Mouse	BPA	200 mg/kg bw/d	Orally (food)	GD-15 to GD12.5	GD12.5	Placental function	Placental number of differentially expressed miRNAs: BPA ↑ (22)/↓ (21) Prediction of the number of mRNAs affected by the differentially expressed miRNAs: 142 Tissue-specific gene enrichment based on predicted mRNAs affected by the differential expressed miRNAs: thymus, cerebellum, olfactory bulb, brain cortex, E14.5 brain, and heart Pathways affected by the differential expressed miRNAs: neural pathways (neurogenesis, neuron differentiation, and development), cell projection organization, cation transmembrane transport, metal ion transport, and inorganic cation transmembrane transport		[144]
Mouse	BPA	10 mg/kg bw/d BPA	Subcutaneous injections	GD0-GD7	GD10, GD12, PND0-56	Placental structure	Number of embryos and survival rate: BPA ↓ Placental size and proportion of placental decidua basalis: BPA ↓ (GD12) Proportion of placental metrial gland: BPA ↑ Proportion of placental labyrinth area, intervillous spaces, and alignment of trophoblast giant cells: BPA ↓	Uterine weight, uterine-to-bodyweight ratio, and glycogen-containing cells: BPA ↓	[137]
Mouse	BPA	5, 10, or 40 mg/kg bw/d	Subcutaneous injections	GD0.5-5.5	GD14.5	Placenta structure and function	Labyrinthine area and decidua ITGβ1 and ITGα5 protein levels: BPA ↑ Placental MMP9 and MMP2 protein level: BPA ↑ Placental TIMP3 protein level: BPA ↓ Phosphorylated Akt and ERK level: BPA ↑ (5, 40) Labyrinthine area proportion and intervillous spaces: BPA ↓ Spongiotrophoblast layer in the placenta: BPA ↓ (10, 40) Presence of large vacuoles in the placenta: BPA ↑	-	[139]
Rat	BPA	0.0025, 0.025, or 0.25 mg/kg bw/d	Orally (drinking water)	GD-30 to GD20-22	GD20-22	Fetoplacental development and uterine artery	Fetal weight: BPA ↑ (2.5) Placental weight and placenta-to-bodyweight ratio: BPA ↓ (2.5, 250)	Uterine artery diameter and uterine artery relaxation: BPA ↓ Uterine artery gene expression: BPA ↓ (*PPARγ*/*ERα*)/↑ (*ERβ*/*VEGF*/*COX-2*)	[133]
Rat	BPA	0.0025, 0.025, or 0.25 mg/kg bw/d	Orally (drinking water)	GD-30 to GD20	GD20	Fetoplacental development/placental metabolism	Fetal weight at birth: BPA ↑ (2.5) Placental weight: BPA ↓ (2.5, 250) Placental efficiency: BPA ↑ Glucose transporter 1 (GLUT1) placental expression: BPA ↑ (2.5, 250)	Non-pregnant rats bodyweight: BPA ↑ (25, 250) 3-week pregnancy bodyweight: BPA ↓ (2.5)	[148]
Rat	BPA	200, 400, or 600 mg/kg bw/d	Subcutaneous injections	GD17-19	PND5	Maternal and fetal uteri gene expression	Postnatal uterine calbindin-D9K (*CaBP-9k*) and Estrogen receptor alpha (*Erα*) mRNA expression: ↑ (600) Placental passage of BPA Rapid BPA absorption and distribution in maternal uteri	Maternal uterine *CaBP-9k* mRNA expression: BPA ↑ (600) Maternal uterine CaBP-9k protein expression: BPA ↑ Maternal uterine *Erα* mRNA expression: no effect Induction of CaBP-9k protein in the endometrium of the maternal uterus: BPA ↑	[147]
Sheep	BPA	0.5 mg/kg bw/d	Subcutaneous injections	GD30-90	GD65, 90	Placental function	Fetal weight and placental efficiency: BPA ↓ (GD65) Placental expression of inflammatory genes: BPA ↓ (GD65: *TNF*)/↑ (GD65: *IL8*/GD90: *IL1β*) Placental expression of vascularization genes: BPA ↓ (GD90) Placental expression of steroidogenic metabolism and signaling genes: BPA ↑ (GD65: *CYP17*, *CYP1*9 and *ESR2)/*↓ (GD90: *CYP17*) Placental expression of IGF family: BPA ↓ (GD65: *IGF1R*)/↑ (GD65: *IGF2R*, *IGFBP1*, *IGFBP3*/GD90: *IGFBP2*, *IGFBP3* and *IGFBP4*) Placental oxidative stress markers and placentome triglycerides and collagen accumulation: BPA ↑ (GD90) Placental antioxidant genes expression: BPA ↑ (GD65) DNA methyltransferase: BPA ↑ (GD65: DNMT3A) Histone deacetylase: BPA ↑ (GD65: HDAC2 and HDAC1)		[134]
Sheep	BPA	5 mg/kg bw/d	Subcutaneous injections	GD40-110	GD110	Placental function/fetal development	Placentome total weight, fetal weight, and placental efficiency: BPA ↓ Relative and protein expression level of Bcl-2: BPA ↓ Apoptosis rate and relative mRNA and protein expression of caspase-3, 8, and 9: BPA ↑ Mitochondrial reactive oxygen species (ROS) production: BPA ↑ ATP content and mitochondrial complex activities: BPA ↓ mRNA and protein relative expression of antioxidant-related genes and protein: BPA ↓ mRNA and protein relative expression of endoplasmic reticulum stress: BPA ↑		[135]
Mouse	BPA, BPS	200 mg/kg bw/d	Orally (food)	GD-15 to GD12.5	GD12.5	Placental structure, function, and metabolism	Number of fetuses and placental oestrone, corticosterone, testosterone and progesterone (P4) concentration: no effect Percentage of male conceptus: BPA ↑ Numbers of differentially expressed genes (DEGs): BPS (11)/BPA (3) Pathways enrichment: Wnt signaling pathway, chemokine signaling pathway, and amino acid metabolism Placental dopamine concentration and percentage of dopamine-positive trophoblast giant cells: BPA ↑/BPS ↑ Placental serotonin concentration and percentage of serotonin-positive trophoblast giant cells: BPA ↓/BPS ↓ Placental estradiol (E2) concentration: BPA ↓ Spongiotrophoblast zone to trophoblast giant cell area ratio: BPA ↓/BPS ↓	Maternal gestational weight, success of pregnancy, and number of implantation sites: no effect D-fructose, sophorose and glycolic acid concentration: BPA ↓ Docosahexaenoic acid (DHA) concentration: BPA ↓/BPS ↓ Stearic acid and palmitic acid concentration: BPS ↓ D-ribose concentration: BPS ↑	[151]
Sheep	BPA, BPS	0.5 mg/kg bw/d	Subcutaneous injections	GD30 to GD100	GD120	Placental function	Endocrine function (pregnancy-associated glycoprotein 1 (PAG1), pregnancy-specific protein B (PSPB), P4): BPS ↓ Placental morphology: no effect Syncytialization: BPS ↓ (E-cadherin)/BPS ↑ (GCM1) Expression of genes involved in trophoblast fusion: BPS ↓		[150]
Rat	BPA, DEHP	0.005 mg/kg bw/d BPA, 5 or 7.5 mg/kg bw/d DEHP, or both (mix)	Oral gavage	GD6-21	GD6 (abortion and weight), PND1 to 24 weeks	Fetal development	Chest circumference: DEHP ↑ (5)/DEHP + BPA ↑ (5)/DEHP ↓ (7.5)/DEHP + BPA ↓ (7.5) Crown-to-rump length: BPA ↑/BPA + DEHP ↑ (5) Heart weight: BPA + DEHP ↑ (♂: 7.5)	Abortion rate: BPA + DEHP ↑ Thymus weight: BPA ↓ (♂)/DEHP ↓ (♂: 7.5) Thymus apoptosis: BPA + DEHP ↑ (7.5)	[158]
Mouse	BPA, OP	50 mg/kg bw/d	Subcutaneous injections	GD11.5-16.5	GD17.5	Placental function	Placental calcium transporter channels: OP ↓ (Trpv6 protein level)/OP ↑ (*Pmca1* mRNA and protein levels), BPA ↑ (*Pmca1* mRNA and protein levels) Placental copper transporter channel expression: OP ↓ (*Crt1* mRNA and protein levels/*ATP7A* mRNA and protein levels)/BPA ↓ (*Crt1* mRNA and protein levels/*ATP7A* mRNA and protein levels) Placental iron transporter channel expression: OP ↓ (*Heph* mRNA)/BPA ↓ (*Heph* mRNA and protein levels) Fetal serum cation levels: BPA ↓/OP ↓ (calcium and copper)		[145]
Rat	BPA, OP, NP	200, 400, or 600 mg/kg bw/d	Subcutaneous injections		GD20	Placental function	Placental *CaBP-9k* mRNA expression: BPA ↑ (200) Extraembryonic membrane *CaBP-9k* mRNA expression: OP ↓ (400/600)/NP ↓ (600) Placental CaBP-9k protein expression: OP ↑ (200)/NP ↑ (200)/BPA ↑ (400, 600) Extraembryonic membrane CaBP-9k protein expression: OP ↓ (400, 600) Fetal uterus *CaBP-9k* mRNA expression: OP ↑ (400, 600)/NP ↑ (400, 600)/BPA ↑ (600)	Maternal uterus *CaBP-9k* mRNA expression: OP/NP ↑ (600) Maternal uterus CaBP-9k protein level: BPA ↑/OP ↑ (600)	[146]
Mouse	BPA, TBBPA	10 mg/kg bw/d BPA, 0.5 mg/kg bw/d TBBPA	Orally (food)	GD-15-GD16.5	E6.5-E10.5, E16.5	Fetoplacental development/placental function	Rate of the hemorrhaging conceptus and fetal loss: BPA ↑/TBBPA ↑ Trophoblast giant cells *Ido1* mRNA expression: BPA ↓	Resorption rate: BPA ↑/TBBPA ↑ Mean Tregs cell number in the maternal spleen: BPA ↓ Mean CD4+ T cell number in the maternal spleen: BPA ↓/TBBPA ↓ Percentage of total Ido1 DNA methylation at CpG sites: BPA ↑ (♂)	[159]
Mouse	BPA, TCS	0, 87, 262, 523, or 7858 mg/kg bw/d TCS in a single dose; 0, 523, or 7858 mg/kg bw/d TCS in repeated doses; 61 or 122 mg/kg BW/d BPA; 262 mg/kg BW/d TCS + 61 mg/kg BW/d BPA; 262 mg/kg BW/d TCS + 122 mg/kg BW/d BPA	Subcutaneous injections	GD1-3	GD6, 17, PND 0, 4, 7, 14, and 21	Implantation	Number of implantation sites: TCS ↓ (repeated-dose GD6: 523, 7858)/TCS ↓ (single-dose GD2: 7858/GD3: 523, 7858)/TCS + BPA ↓ (GD6: 262 + 122) Percentage of dams with normally developing implantation sites: TCS + BPA ↓ (GD6) Gestational length: BPA + TCS ↑ (262 + 122) Postnatal survival and pup bodyweight: no effect		[152]
Mouse	TCS	1, 4, or 8 mg/kg bw/d	Oral gavage	GD6-18	GD11-19	Fetoplacental development/placental function	Number of live fetuses, fetal bodyweight, and placental weight: TCS ↓ (8) Placental size and volume, labyrinthine area volume, and labyrinth cell proliferation activity: TCS ↓ (8) *PCNA* and *CD3* placental expression: TCS ↓ (8) MeAIB placental expression: TCS ↑ (8) MeAIB transporteur activity (SNAT): TCS ↓ (8) Glucose transporter activity: TCS ↓ (8: *SNAT1*, *SNAT4*, *GLUT1* mRNA) Phosphorylation of Akt-mTOR-p70S6K signaling: TCS ↓ (8)	Triiodothyronine (T3), thyroxine (T4) and P4 levels: TCS ↓ (8) Thyroid-stimulating hormone (TSH) level: TCS ↑ (8)	[155]
Mouse	TCS	4 or 8 mg/kg bw/d	Oral gavage	GD6-14	PND1, 30, 60	Placenta/metabolism	Numbers of pups, offspring bodyweight, and body mass index: TCS ↓ (8) Body length: no effect		[160]
Mouse	TCS	0, 10, 50, or 100 mg/kg bw/d	Oral gavage	GD7.5-17.5	GD17.5	Placental function	Fetal and placental weight: TCS ↓ (50, 100) Placental expression of *PPARγ* and *PPARγ*-regulated genes, and ANGPTL4 and MMP9 proteins: TCS ↓ Placental expression of inflammatory genes and IL-1β proteins: TCS ↑	Uterus size: TCS ↓ (50, 100)	[161]
Rat	TCS	75, 150, or 300 mg/kg bw/d	Oral gavage	GD8-PND21	PND3, PND90	Fetal development/uterine structure/estrous cycle	Mean of litter weight: TCS ↓ (PND3) Number of estrus cycles and frequency of proestrus phase: TCS ↓ Frequency of metestrus phase: TCS ↑	Maternal bodyweight: TCS ↓ The papilliferous appearance of the simple columnar luminal epithelium with vacuolization in the uterus: TCS ↑ Uterine cell height and uterine thickness: TCS ↑ Inflammatory infiltrate amount (leukocytes): TCS ↑ (150, 300) T4, T3, and TSH levels: TCS ↑	[162]
Sheep	TCS	0.1 mg/kg bw/d	Direct infusion into the fetal circulation or through administration to the ewe	2 days between GD120 and GD130	3 days after 1st injection	Placental/liver sulfotransferase activity	TCS concentration: Placental concentration > Fetal liver concentration Estrogen sulfotransferase activity in placenta and fetal liver cytosol: TCS ↓		[153]
Mouse	DEHP	0, 50, or 200 mg/kg bw/d	Oral gavage	GD0-6, GD7-12, GD13-17	GD18	Fetoplacental development	Fetal weight: DEHP ↓ Crown–rump length: DEHP ↓ (GD7-12/GD13-17) Placental weight, blood sinusoid area in the labyrinth, and placental cell proliferation: DEHP ↓ (GD7-12) Placental diameter: DEHP ↓ (♂: GD7-12)		[163]
Mouse	DEHP	125, 250, or 500 mg/kg bw/d	Oral gavage	GD1-13	GD9 and GD13	Placental development and function	Embryo implantation: DEHP ↓ (500) Fetoplacental weight, placental-to-body-weight ratio, and ectoplacental cone proportion: DEHP ↓ Condensed packed cells of ectoplacental cone: DEHP ↓ (250, 500) Spongiotrophoblast area in the placenta: DEHP ↓ Labyrinth area in placenta: DEHP ↓ (500) Gene expression involved in placental development: DEHP ↓ (GD9-13: *Ascl2*, *Esx1*, and *Fosl1*/500 GD9: *Eomes*)/↑ (GD13: *Eomes* and *Hand1*) Formation of the branched fetal vessel in the labyrinthine area: DEHP ↓ MAPK signaling pathway and phospho-Erk1/2 levels in placenta: DEHP ↑ (GD9/GD13: 500)	Protein levels involved in apoptosis process: DEHP ↑ (GD13: Bax casp-3 and Bax caps-8)/↓ (GD13: Bcl2) Number of Ki-67-positive cells: DEHP ↓	[164]
Rat	DEHP	500 or 1000 mg/kg bw/d	Oral gavage	GD7-12	GD20	Fetal development/placental function	Number of living fetuses: DEHP ↓ Fetus malformation: DEHP ↑ Number of differentially expressed genes (DEGs): DEHP ↑ (500 (1951), 1000 (951))/↓ (500 (1836), 1000 (527)) DEG pathways: steroid biosynthesis, PPAR signaling pathway, amino acid metabolism, lipid and drug metabolism, circadian entrainment, and neuroactive ligand–receptor interaction	Pregnant weight changes at GD18: DEHP ↓ Protein-protein interaction: 2682 interaction pairs and 476 nodes Protein–protein interaction enrichment: olfactory transduction, cell cycle pathways, and neuroactive ligand–receptor interactions	[165]
Rat	DEHP	750 or 1500 mg/kg bw/d	Oral gavage	GD0-19	GD20	Placental metabolism	*PPARα* and *PPARγ* mRNA expression in the labyrinth and the junctional zone: DEHP ↑ PPARα and PPARγ protein expression: DEHP ↑ Essential fatty acid (EFA) transporters in the labyrinth: DEHP ↑ (FAT/CD36, FATP1, HFABP) EFA transporters in the junctional zone: DEHP ↑ (FATP1, HFABP) EFA metabolic enzyme expression: DEHP ↑ (CYP4A1)/↓ (COX-2) Total prostaglandins in the placenta: DEHP ↓	Arachidonic acid (AA) in fetal and maternal plasma: DEHP ↓ DHA in fetal plasma: DEHP ↓ DHA in maternal plasma: DEHP ↑ AA and DHA maternal–fetal ratio: DEHP ↑ Fetal organ distribution of AA and DHA: DEHP ↓	[166]
Rat	DnHP, DCHP	0, 20, 100, or 500 mg/kg bw/d	Oral gavage	GD6-19	GD20	Fetoplacental development	Fetal bodyweight: DnHP ↓ (20, 100)/DnHP ↑ (♂: 500)/DCHP ↓ (♀: 500)/DCHP ↑ (20, 100) Anogenital distance (AGD): DnHP ↓ (♀)/DCHP ↓ (♀) AGD/bodyweight ratio: DnHP ↑ (100)/DnHP ↓ (500)/DCHP ↓ (20, 100) Placental weight: DCHP ↑/DnHP ↑ (20, 100) Placental diameter: DnHP↓/DCHP ↓ Placental index: DnHP ↑/DCHP ↑ Trans-umbilical cord length: DnHP ↓ (20, 100)/DCHP ↑ (500) Cytoplasmic degeneration of trophoblast giant cells: DnHP ↑/DCHP ↑ Number and volume of trophoblast giant cells: DnHP ↑ (100, 500)/DCHP ↑ (500) Degeneration of spongiotrophoblast cells: DCHP ↑ (100, 500) Hemorrhage in the labyrinth and basal zone: DnHP ↑ (100, 500)/DCHP ↑ Vessel formation in the labyrinth: DnHP ↑ (500)/DCHP ↓ Immunostaining of PCNA, PPARγ, ERα, ERβ, and AR: DCHP↓/DnHP↓	F0 relative kidney and liver weights: DCHP ↑ (20)/DnHP↓ (500) Dams relative organs weights: DCHP ↑ (500) Number of implantation sites: DCHP ↓ (500) Number of live fetuses: DCHP ↓/DnHP ↓ (500) Absolute and relative lengths of bone ossification centers: DCHP ↓/DnHP ↓ Leukocyte, lymphocyte, and monocyte levels: DnHP ↑/DCHP ↑ N-granulocyte levels: DnHP ↓/DCHP ↓ Red blood cell parameters: DnHP ↑ (♂: 500)/DCHP ↑ (♂: 20)	[167]
Rat	n-BuP	0, 10, 100, or 1000 mg/kg bw/d	Oral gavage	GD6-19	GD20	Fetal development	Litter size, survival rate, number of resorptions, sex ratio, and fetal bodyweight: no effect	Dams bodyweight and bodyweight changes: no effect Dams bodyweight gains: n-BuP ↓ (GD18-20: 1000) Absolute and relative dams feed consumption: n-BuP ↓ (GD6-20: 1000)	[168]
Mouse	n-BuP, PrP	0, 0.05, 0.5, 5,10, 20, 30, and 35 mg/dams n-BuP; 35 or 40 mg/dams PrP	Subcutaneous injections	GD1-4	PND0, 3, 5	Fetal development	Skin irritation after injection: n-BuP ↑ (0.5, 5, 10, 20, 30, 35) Litter size, survival rate, litter mass, gestational length, and number of implantation sites: no effect		[169]
Rat	DiBP, BP	600 mg/kg bw/d DiBP or 100 mg/kg bw/d n-BuP	Oral gavage	GD7-19 or GD7-21	GD19-21	Fetal metabolism	Plasma leptin level: DiBP ↓/n-BuP ↓ Plasma insulin level: DiBP ↓ Fetal bodyweight: DiBP ↓ (GD19) Liver *PPARα* mRNA expression: DiBP ↓ (GD19) Liver and testes *PPARγ* mRNA expression: no effect Fetal anogenital distance: DiBP ↓ (♂)/↑ (♀)	Dams bodyweight gain: no effect Testicular mRNA expression of genes involved in steroid synthesis: DiBP ↓ (GD19: *SF-1*)/↑ (GD21: aromatase) Testes *PPARα* mRNA expression: DiBP ↓ (GD19) Ovaries mRNA expression of genes involved in steroid synthesis: no effect 17α-hydrolase (P450c17) and PPARγ protein level in Leydig cells, and testicular testosterone production and level: DiBP ↓ (GD19/GD21)	[170]
Rat	EtP, n-BuP	100, 200, or 400 mg/kg bw/d	Subcutaneous injections	GD7-21	GD21	Placental function	Number of fetuses, fetus weight, and fetus organ weight: no effect	Maternal weight: no effect	[157]
Mouse	EtP, PrP	0, 400, 800, or 1600 mg/kg bw/d EtP, and 0, 625, 1250, or 2500 mg/kg bw/d PrP	Oral gavage	GD1 to GD5-7 or to GD8-9	GD5, GD7-8, GD10	Implantation/endocrine function	Marker expression of decidualization: EtP ↓ (1600)/PrP ↓ (2500) *ER* and *PR* expression: EtP ↓ (1600)/PrP ↓ (2500) Rate of F1 litter size < 7: EtP ↑ (1600)/PrP ↑ (2500)	Implantation rate: EtP ↓/PrP↓ Uterus weight: EtP ↓ (800, 1600)/PrP↓ (2500) E2 and P4 serum levels: EtP ↑ (1600)/PrP ↑ (2500)	[171]

Results in brackets correspond to the sex in which the effect was observable (♂, male; ♀, female), followed by stage at observation separated by a column from the doses administered at which the effect was observed, and, if so, followed by the number of DEGs in brackets. If one of the above-mentioned characteristics is not specified, the effect was observed for both sexes at the different stages of observation and the different doses used. Arrows indicate the direction of the effect: a downward arrow (↓) indicates a decrease and an upward arrow (↑) indicates an increase. ANGPTL4: angiopoietin-like protein 4; Ascl2: achaete-scute family bHLH transcription factor 2; ATP: adenosine triphosphate; ATP7A: ATPase Cu++ transporting alpha-polypeptide; BPA: bisphenol A; BPS: bisphenol S; n-BuP: butylparaben; bw: bodyweight; cAMP: cyclic adenosine monophosphate; CD3: cyclin D3; Crt1: solute carrier family 31: member 1; COUP-TFα: chicken ovalbumin upstream promoter transcription factor alpha; COX-2: cyclooxygenase-2; CRH: corticotropin-releasing hormone; CYP19: placental aromatase; CYP17: 7 alpha hydroxylase; CYP4A1: cytochrome P450 subfamily 4A1; d: day; DCHP: dicyclohexyl phthalate; DEHP: di(2-ethylhexyl) phthalate; DnHP: di-n-hexyl phthalate; DiBP: diisobutyl phthalate; DNMT3A: DNA methyltrasnferases; Eomes: eomesodermin; EtP: ethylparaben; ERK: extracellular signal-regulated kinase; ESR2: estrogen receptor 2; Esx1: extraembryonic, spermatogenesis, homeobox 1; F1: first generation; FAT/CD36: fatty acid translocase; FATP1: fatty acid transport protein 1; Fosl1: Fos-like antigen 1; GCM1: glial cell missing factor 1; GCNF: germ cell nuclear factor; GD: gestational day; Hand1: heart and neural crest derivatives-expressed 1; HDAC: histone deacetylases; Heph: hephestin; HFABP: heart cytoplasmic fatty acid-binding protein; Ido1: indoleamine2,3 deoxygenase1; IGF1R: insulin-like growth factor 1 receptor; IGF2R: insulin-like growth factor 2 receptor; IGFBP: insulin-like growth factor-binding protein; IL-1β: interleukin 1 beta; IL-8: interleukin 8; LXRα: liver X receptor alpha; MAPK: mitogen-activated protein kinase; MeAIB: methylaminoisobutyric acid; miRNAs: microribonucleic acids; MMP: metalloproteinase; mRNA: messenger ribonucleic acid; NP: 4-nonylphenol; OP: 4-tert octylphenol; PCNA: proliferating cell nuclear antigen; PMCA1: plasma membrane Ca^2+^ ATPase; PND: postnatal day; PNR: photoreceptor-specific nuclear receptor; PrP: propylparaben; SF-1: steroidogenic factor-1; SNAT: sodium-coupled neutral amino acid transporter; TBBPA: tetrabromobisphenolA; TCS: triclosan; TIMP: tissue inhibitor of metalloproteinase; TNF: tumor necrosis factor alpha; Tregs: regulatory T cells; Trpv6: transient receptor potential cation channels in subfamily V member 6; VEGF: vascular endothelial growth factor; WNT: Wnt family member.

## 6. Effects of PPPs on Offspring Health after Birth from Epidemiological Data and Using Animal Models

Guilbert et al. and Radke et al. suggested that PPPs impact child neurodevelopment (including cognition and behavior) [39,54]. In this review, only the effects of PPPs in terms of obesity, metabolic alterations, cardiovascular disorders, and fertility defects will be explored.

### 6.1. Effects of PPPs on Obesity

#### 6.1.1. Epidemiological Data (Table 3)

Few studies have examined the effects of prenatal PPP exposure on postnatal adiposity and growth [113,172,173,174,175]. Regarding phenols, we identified 16 studies from nine countries (Canada, China, Denmark, France, Greece, Mexico, Republic of Korea, Spain, and the USA) that examined the association between maternal phenol concentrations measured in urine and postnatal weight and adiposity in children. Sample sizes ranged from 218 to 1301 [173,175,176]. The outcomes measured varied across studies, with weight, BMI, waist circumference, and fat being measured from 6 months to 14 years of age [177]. Of the twelve studies evaluating BPA, eight found no statistically significant associations [113,172,173,174,176,177,178,179], one of 402 mother–child pairs in the USA reported a negative association with BMI, fat mass, and obesity risk at 2 to 9 years in girls [180]. Three other studies found positive associations with obesity markers. These three studies were conducted in Canada (N = 719) [181], China (N = 430) [182], and Republic of Korea (N = 788) [183]. Despite using a limited number of urine samples to assess the exposure (one or two), these studies reported positive associations between prenatal BPA and the waist-to-hip ratio at an average of 3.5 years (range 2–6 years), the waist circumference and an increased risk of obesity at 7 years, and the z-score weight from 2 to 6 years in girls. One study, from the SEPAGES cohort (N = 484), reported a positive association between second-trimester BPS and all infant growth parameters at 3 and 36 months of age [175]. No studies found an association between prenatal exposure to 2,4-dichlorophenol and 2,5-dichlorophenol. However, the number of studies on these compounds was relatively small (two to three). Among the small number of studies examining the association between BP-3 and childhood adiposity, a study of preadolescents (mean age = 11 years) in Spain (N = 1015) reported a positive association between prenatal exposure to BP-3 and a higher BMI z-score at 11 years [174].

Among the five studies of TCS, only one conducted in China reported a positive association with weight at 2 years using three spot urine samples (one from each trimester of pregnancy, N = 850 women–child pairs) [184]. This association appears to be stronger in girls. It is noteworthy that this study was one of the largest in sample size and number of urine samples collected during pregnancy.

We identified six studies regarding parabens. Four of them reported positive associations with postnatal weight, BMI, or the fat percentage at different ages, while the significantly associated paraben compounds differed between studies [172,178,185,186]. Three of these studies reported such effects in boys [178,185,186].

Regarding phthalates, 24 studies were identified. These studies assessed the association between prenatal phthalate concentrations and postnatal adiposity and growth. They were conducted in Australia, the USA, China, France, Germany, Greece, Mexico, Republic of Korea, and Spain. Sample sizes ranged from 180 [187] to 1301 [173]. Most of the studies measured phthalate metabolites in maternal urine collected at 1–3 time points during pregnancy. Serum was used in one study in Australia, resulting in lower detection frequencies and a higher risk of external contamination. In addition to these individual studies, a recent meta-analysis found a negative association between prenatal exposure to DEHP and child BMI [188]. A limitation of this meta-analysis was that only studies assessing associations with the molar sum of all the metabolites of DEHP were considered. This led to the exclusion of studies examining each metabolite separately, while several actually reported positive associations [79,189]. For other high-molecular-weight phthalates, isolated negative associations have been reported in boys between ∑DiNP and lean mass at 20 years [190], between ∑HMWP (high-molecular-weight phthalates) and weight gain during the first 6 months of life, and with BMI from 4 to 7 years [191]. A few studies also reported associations between exposure to MCPP, cx-MiNP, and MCOP and an increased risk of overweight [172,187], higher BMI [192], waist circumference [193], and lean mass [79]. For MBzP, the results were inconsistent, with four studies reporting positive associations [192,193,194,195] and two studies reporting negative associations [177,196] with obesity markers. Among the studies of low-molecular-weight phthalates, two studies reported positive associations between ∑LMWP (low-molecular-weight phthalates) and child BMI. However, this association was observed in boys in one study [190] and in girls in the other [79]. Regarding individual metabolites, of the 19 studies evaluating MEP, 6 studies reported positive associations with BMI [79,193,197], and only 1 study reported an inverse association with fat mass [187]. For MBP, a metabolite of DBP, most of the significant associations reported were positive, suggesting increased adiposity [193,198] and BMI [175,192,195]. Two studies have also reported positive associations between MiBP and BMI [79,190,192].

Overall, most studies examining the associations between prenatal phthalate exposure and child growth or BMI reported associations. However, the sign of the associations and the metabolites involved often differed. A meta-analysis, such as the one carried out for DEHP, could help to understand the links between prenatal exposure to phthalates and child growth. Although phthalates may act through common mechanisms, to date, very few studies have explored them as a mixture [79,113,172,173,199].

**Table 3:** Effects of phenols, parabens, and phthalates on adiposity and growth of offspring using epidemiological data.

Population	Class	Chemicals	Sampling Type, Number, and Timing	Timing of Outcome and Outcome of Interest	Model	Sex-Specific Analyses	Main Findings	Reference
INMA Spain N = 470	Phenols and Phthalates	MEP, MBP, MiBP, MBzP, OH-MiNP, MECPP, MEHHP, MEOHP, MEHP, MCMHP, BPA	Urine N = 2 first and third trimesters	7 years old Body mass index (BMI) z-scores	Uni-pollutant and multi-pollutant using PCA	Yes; *p*-values for interaction >0.2;	Compared to the first tertile, 7OHMMeOP was inversely associated with the BMI z-score in the third tertile and MECPP was inversely associated with overweight in both the second and the third tertiles of exposure. In the PCA analysis, nonsignificant negative associations were observed with the zBMI for exposure to the phthalate factor (factor 2) in tertile 3 and tertile 2 compared with tertile 1.	[113]
CHAMACOS California, USA N = 309	Phenols, Phthalates, and Parabens	MEP, DEP, MBP, DBP, MiBP, DiBP, MBzP, BBP, DEHP, MEHP, MEHHP, MEOHP, MECPP, DiNP, MCOP, cx MiNP, DiDP, MCPP, MeP, PrP, TCS, 2,4-DCP, 2,5-DCP, BP-3, BPA	Urine N = 2 2 prenatal visits	5 years old BMI z-score and overweight/obesity status	Uni-pollutant and multi-pollutant using BKMR	-	Urinary concentrations of MEP, cx-MiNP, and propylparaben were consistently associated with an increased BMI z-score and overweight/obesity status.	[172]
Raine Study Australia N = 410	Phthalates	MEP, MiBP, MBP, MBzP, MEHP, MECPP, MCMHP, MCPP, MiNP, MCOP, MiDP, ΣMBP, ∑DEHP, ∑DiNP, ∑LMW, ∑HMW, ∑all.phth.metab	Serum N = 2 18 and 34 gestational weeks (GWs)	From birth and up to 20 years of age, longitudinal BMI z-scores, and DEXA at 20 years	Uni-pollutant, and linear mixed models with an interaction term between the phthalate level and age group (0–2, 2–11, and 11–20 years)	-	Compared to the lowest tertile: Childhood BMI was positively associated with the middle tertile of MiBP and adolescent BMI is positively associated with the middle tertile of MiBP and ∑LMW. For fat mass, the highest tertile of MECPP had a lower total fat percentage at 20 years of age. Participants whose mothers had detectable MiDP levels had a higher total fat percent at 20 years of age than those with undetectable MiDP levels. For lean mass, there was a positive association with detectable MCOP levels and an inverse association with the middle or highest tertiles of MEHP and the middle tertile of MiNP or ∑DiNP.	[190]
EDEN France N = 520	Phthalates	MEP, MBP, MiBP, ΣLMW, MECPP, MEHHP, MEOHP, MEHP, ΣDEHP, MBzP, MCOP, MCPP, cx-MiNP, ΣHMW	Urine N = 1 between 22 and 29 GWs	Measures at birth and 5 years, prediction at 1, 3, and 5 years Birth weight, BMI, and Jenss–Bayley modeling approach of weight	Uni-pollutant	Only boys	No association between phthalate metabolite concentration and postnatal longitudinal weight. Positive association between MEP and weight and BMI at 5 years old, and weight velocity. Positive association between MBzP and weight at 2 years old and weight velocity at early ages.	[194]
ELEMENT Mexico N = 223	Phthalates	MEP, MBP, MiBP, MCPP, MBzP, MECPP, MEHHP, MEHP, MEOHP, ΣDEHP	Urine N = 3 one sample per trimester	Around 10 and 13 years old Skinfold thickness, BMI-for-age z–score; waist circumference (WC)	Uni-pollutant, generalized estimating equation models with repeated-measures outcome	Yes, all analyses were sex-stratified	Among females, positive association between MBP T1, and BMI and MiBP T1, with the 3 outcomes. Negative association between MBzP T2 and skinfold thickness. Among males, only MBzP T2 was positively associated with BMI and waist circumference.	[195]
MIREC study Canada N = 719 mother–child pairs	Phenols	BPA	Urine N = 1 first trimester (mean 12.1 GWs [6.3 to 15])	Average 3.5 years old (range: 1.9–6.2) weight, height, waist/hip circumference, and subscapular/triceps skinfold thickness	Uni-pollutant, linear regression	Yes	BPA was positively associated with the waist-to-hip ratio among all children. Among girls, BPA was positively associated with the waist circumference (while null in boys) and the subscapular skinfold thickness (while almost inversely in boys).	[181]
Mount Sinai USA N = 180	Phthalates	MEP, MBP, MiBP, MCPP, MBzP, ∑DEHP	Urine N = 1 T3 trimester (25–40 GWs)	Each follow-up visit scheduled at approximately ages 4–5.5 (mean, 4.9), 6 (mean, 6.2), and 7–9 (mean, 7.8) years old Fat mass	Linear mixed-models with random intercepts to account for multiple observations per child; Bayesian modeling framework	Yes	No significant association between using continuous chemicals. In tertiles, compared with the lowest ∑DEHP tertile, the fat mass was 3.06% (95% CIs: –5.99, –0.09%) lower in the highest tertile.	[200]
The Mount Sinai Children’s Environmental Health Study (MSSM), CCCEH, HOME USA N = 180	Phthalates	MEP, MBP, MiBP, MCPP, MBzP, ∑DEHP	Urine N = 1 third trimester (20–40 GWs)	At follow-up visits scheduled for approximately ages 4– 5.5, 6 and 7–9 years old (MSSM), 5 and 7 years old (CCCEH), and 4, 5, and 7–9 years old (HOME) BMI z-score and overweight/obese status	Bayesian modeling framework	Yes	MCPP was associated with increased odds of overweight/obese status overall. MEP was associated with lower BMI z-scores among girls.	[187]
Odense Child cohort Denmark N = 312	Parabens	MeP, EtP, PrP, b-BuP, BzP	Urine N = 1 around 28 GWs (median 28.7 GWs)	7 years old BMI z-score, total fat, android fat, and gynoid fat	Uni-pollutant, linear regression	Yes, all analyses stratified by gender	Only n-BuP was positively associated with the total and android (visceral and subcutaneous) fat percentage among boys.	[185]
TIDES USA N = 780	Phthalates	MEP, MBP, MiBP, MCPP, MCOP, cx-MiNP, MBzP, ∑DEHP	Urine N = 2 around 11 and 32 GWs	Birth, 1, 3, 4, and 6 years old Weight and BMI	1—Linear mixed-model with repeated growth measures; 2—Group-based trajectory (patterns of weight or BMI change over time)	Yes, differences by sex for weight models but not BMI models	MEP, MBzP, MiBP, and MBP were all inversely associated with weight and BMI at birth. MCPP and MBP were positively associated with BMI at 3 years old. MEP, MBzP, MCPP, and MCOP were positively associated with BMI at 4 years old.	[192]
Ma’anshan Birth Cohort (MABC) China N = 990	Phthalates	MMP, MEP, MBP, ΣLMW, MBzP, MEHP, MEOHP, MEHHP, ΣDEHP, ΣHMW	Urine N = 3 each trimester	Birth, 3, 6, and 9 months, and 1, 1.5, 2, 2.5, 3, 4, 5, and 6 years old BMI for age z-scores	Uni-pollutant (linear/logistic regression and GEE with repeated measurement) and multi-pollutant (quantile g-computation and BKMR)	Only girls	Pregnancy means ΣLMW and MBzP were negatively associated with BMI z-score at birth. Pregnancy means ΣDEHP was positively associated with BMI z-score at 3 months. Trajectories of BMI z-score was associated with first-trimester exposure to MEP (negative association) and MEOHP (positive association), and positively associated with third-trimester exposures to MEP, MBP, ΣLMW, and MEHP. Mixtures analyses did not find any significant associations.	[201]
INMA Spain N = 1015	Phenols, Parabens, and Phtalates	MEP, MBP, MBzP, DEHP, MeP, EtP, PrP, n-BuP, BP-3, BPA	Urine N= 2 first (mean 13 GWs) and third (mean 33 GWs) trimesters	11 years old BMI z-scores	Uni-pollutants (generalized additive mixed models); multi-pollutant (BKMR)	Yes	BP-3 was associated with a higher BMI. In girls, the overall mixture trended with a higher BMI.	[174]
HELIX project European cohort including France, Greece, Lithuania, Norway, Spain, and the UK N = 1301	45 ED compounds	Among them, 7 high-molecular-weight phthalate metabolites (HMWPs), including 4 (DEHP) metabolites, 2 DiNP metabolites, and 1 metabolite of BBP; 3 low-molecular-weight phthalate metabolites (LMWPs); 4 parabens; 3 phenols	Urine and blood samples during pregnancy N = 1134	6 to 11 years old	Associations were assessed using Bayesian weighted quantile sum regressions applied to mixtures for each chemical	Yes	ΣLMW and ΣHMW of phthalates: ↓ MetS risk score. Association of prenatal MnBP levels with ↓ child MetS risk score. Phenols and parabens: no association with the MetS risk score.	[202]
China N = 436	Parabens	MeP, EtP, PrP, n-BuP, BzP	Urine N = 1 3 years old	3 years old Weight and BMI	Uni-pollutant generalized linear models	Yes	Among all and among boys: urinary EtP concentrations were positively associated with weight z-scores. Nothing significant among girls.	[186]
Sheyang Mini Birth Cohort Study China N = 430	Phenols	BPA	Urine N = 1 delivery day	7 years old Weight, WC, skinfold thickness, and risk of general and central obesity	Uni-pollutant, generalized linear regression models, and multivariable logistic regression models	Yes, no significant interaction	Positive associations with the waist circumference. Higher risk of central obesity in the second and third tertiles of BPA compared to the first tertile.	[182]
CHAMACOS California, USA N = 345	Phthalates	MEP, MBP, MiBP, MCPP, MCOP, cx-MiNP, MBzP, ∑DEHP	Urine N = 2 around 14 and 26.9 GWs, averaged for analyses	5, 7, 9, 10.5, and 12 years old Overweight/obesity, BMI z-score, WC z-score, and percent body fat	Uni-pollutant, GEE with repeated outcomes	Yes	MEP, MBzP, and ΣDEHP were associated with a 20–30% increase in the odds of overweight/obesity at each age point in both boys and girls. MBP was associated with a 30–40% increase in odds in boys only. Consistent positive associations of MEP with BMI, waist circumference, and percent body fat at each time point. ΣDEHP associated with increased waist circumference at 5 years old. MBP, MiBP, MBzP, MCOP, and cx-MiNP were associated with increased waist circumference at 7 and 9 years old.	[193]
CHAMACOS California, USA N = 402	Phenols	BPA	Urine N = 2 first (mean 13.8 GWs) and second (mean 26.4 GWs) trimesters	2, 3.5, 5, 7, and 9 years BMI, WC, percent body fat, and obesity	Uni-pollutant, association with outcomes at 9 years: linear/logistic regressions. Association with longitudinal outcomes: GEE	Yes	Association was not significant among all, while there was a negative association among girls (significant negative association with BMI, body fat, and risk of overweight/obesity), and null associations among boys. Longitudinal analysis showed a negative association with the BMI z-score among girls.	[180]
CHAMACOS California, USA N = 335	Phthalates	MBP, MEP, MiBP, MBzP, cx-MiNP, MCOP, MCPP, MEHP, MEHHP, MECPP, MEOHP	Urine N = 2 around 14 and 26.9 GWs, averaged for analyses	11 follow-up visits between 2 and 14 years old BMI trajectories	PCA	Yes	MEP-positive association with BMI through 12 years.	[197]
HOME USA N = 220	Phenols	TCS	Urine N = 2 around 16 and 26 GWs	8 years old Weight, WC, and body fat percentage	Uni-pollutant, linear regression	yes, all *p*-values = 0.37	No significant association.	[203]
PROGRESS Mexico N = 514	Phthalates	ΣDEHP, ΣDiBP, ΣDiNP, ΣDBP, MBzP, MECPTP, cx-MiNP, MCPP, MEP	Urine N = 2 s and third trimesters	4, 6 and 8 years old Child adiposity was categorized into 3 trajectories	Uni-pollutant (linear and multinomial logistic regressions) and mixture (quantile G-computation)	Yes	In comparison to the “low-stable” group, ΣDEHP was associated with greater odds of being in the “high–high” trajectory, ΣDiNP was associated with greater odds of being in the “low–high” trajectory, and cx-MiNP was associated with lower odds of being in the “low–high” trajectory. No mixture effects.	[199]
EDC Cohort Republic of Korea N = 481	Phthalates	MEHHP, MEOHP, ΣDEHP, MBP	Urine N = 1 second trimester (mean 20.3 GWs)	6 years old BMI z-score, percentage of fat mass, and FMI	Uni-pollutant, linear regressions	Yes	MEHHP and MnBP were inversely associated with the BMI z-score among girls. All chemicals measured were inversely associated with skeletal muscle index among all children and among girls (not among boys).	[204]
MOCEH Study Republic of Korea N = 788	Phenols	BPA	Urine N = 1 third trimester	Birth, and 6, 12, 24, 36, 60, and 72 months Weight z-score and z-score of weight for length	Uni-pollutant, linear regression for each outcome, and LMM for growth from 6 to 72 months	Yes	BPA was positively associated with the weight z-score at birth among all children and among boys, while positively associated among girls at 24, 36, 60, and 72 months. BPA was positively associated with the z-score of weight for length among all children and among girls at birth, 6, 36, 60, and 72 months. In the longitudinal analysis, BPA was positively associated with the z-score of weight for length among all children and among girls.	[183]
Human cohort conducted at Wuhan, China N = 814	Phthalates	MECPP, MEHHP, MEOHP, MEHP, ∑DEHP	Urine N = 3 each trimester	Birth, 6, 12, and 24 months old Weight, ponderal index (PI), and BMI	Uni-pollutant—mixed linear models	Yes	MECPP and MEOHP were positively related to the average weight z-scores in male offspring. DEHP levels at T3 were positively related to 6-month and 12-month BMI z-scores among all children and boys. DEHP levels at T1 were positively associated with BMI z-scores at 24 months among boys.	[189]
CCCEH USA N = 424	Phthalates	MEHP, MEHHP, MECPP, MEOHP, MCPP, MiBP, MBP, MBzP, MEP	Urine N = 1 third trimester	5 (only BMI) and 7 years old BMI z-scores, percentage body fat, fat mass index, and WC	Analyses by DEHP component and non-DEHP component; GEE for BMI analyses, linear regression for other outcomes	Yes	Non-DEHP components were negatively associated with all outcomes of interest among boys.	[205]
German LIFE Child cohort study, Germany N = 164	Phthalates	MBzP, MEHP, MEHHP, MEOHP, MECPP, MCIOP, oxo-MiNP, OH-MiNP, ∑LMW, ∑HMW	Urine N = 1 late pregnancy (24 or 36 GWs)	Birth, 1 and 2 years old Weight and BMI	Uni-pollutant, robust linear regression	Yes	∑HMW was negatively associated with birth weight in all children and among girls (not boys).	[206]
Shanghai Obesity and Allergy Birth Cohort Study, China N = 218	Phenols	BPA	Urine N = 1 late pregnancy	2 years old Weight, mid-upper-arm circumference (MUAC), and skinfold thickness (triceps, subscapular, and abdominal)	Uni-pollutant, linear regression	Yes, all analyses stratified by gender	No differences were found in any adiposity measures.	[176]
EDEN France N = 520	Phenols and Parabens	2,4-DCP, 2,5-DCP, BPA, BP-3, TCS, MeP, EtP, PrP, n-BuP	Urine N= 1 between 22 and 29 GWs	6 months, 1, 2, and 3 years old Birth measurement and postnatal Jenss growth trajectories	Uni-pollutant	Only boys	Parabens were positively associated with weight at birth; remained for 3 years for methylparaben.	[178]
HOME, USA N = 219	Phthalates	∑DEHP, MCPP, MBP, MiBP, MEP, MBzP	Urine N = 2 around 16 and 26 GWs	8 years BMI, WC, and percent body fat	Uni-pollutant, 2 samples averaged for prenatal exposure estimation	Yes	MBzP was negatively associated with the BMI z-score. MBzP was negatively associated with body fat when adjusted for creatinine.	[196]
Rhea Study, Greece N = 230	Phthalates	MEP, MBP, MiBP, MBzP, MEHP, MEHHP MEOHP, ΣDEHP	Urine N = 1 first trimester	4 and 6 years Weight, BMI, WC, and skinfold thickness	Uni-pollutant, GAMs to explore shape GEE for associations with repeated outcomes	Yes	Only MnBP was associated with a change in waist-to-height ratio at ages from 4 to 6 years old.	[198]
Rhea Study, Greece N = 235	Phenols	BPA	Urine N = 1 first trimester	Birth (weight), from 6 to 4 years old (BLI, at 4 years for other outcomes) Weight, BMI, WC, skinfold thickness	Uni-pollutant, first GAM to explore the shape of the relationships, then linear regressions; mixed effects linear regression model for BMI trajectories	Yes	No significant association, but patterns of BMI trajectories differed between boys and girls (only visually; not tested).	[179]
INMA-Sabadell Spain N = 391	Phthalates	ΣHMW, ΣLMW	Urine N = 2 first (around 13 GWs) and third trimesters (around 34 GWs)	From birth to 6 months old for weight gain, and 1, 4, and 7 years old for BMI Weight and BMI	Uni-pollutant and one multi-pollutant model simultaneously adjusted for ΣHMW and ΣLMW; GAMs to explore shape GEE for associations with repeated outcomes	Yes	ΣHMW was inversely associated with the weight gain z-score (from 0 to 6 months) and BMI z-scores at 4 and 7 years old among boys, while associations tended to be positive among girls. Zero associations among all children.	[191]
HELIX Spain N = 1301	Phenols, Parabens, and Phthalates	MEP, MiBP, MBP, MBzP, MEHP, MEHHP, MEOHP, MECPP, OH-MiNP, oxo-MiNP, ΣDEHP, EtP, PrP, n-BuP, BP-3, BPA, TCS	Urine N= 2 first and third trimesters	One visit between 6 and 11 years old Weight and BMI	Uni-pollutant and DSA	Not with phenols and phthalates	No significant association.	[173]
China N = 850	Phenols	TCS	Urine N = 3 one sample per trimester	6 months, 1 and 2 years old z-scores of weight	Uni-pollutant trimester-specific expo and average of expo T1-T2-T3	Yes	Prenatal exposure (mean expo) to triclosan was associated with elevated 2-year-old weight z-score in all and in girls. Exposure during T1 and T2 was associated with increased weight at 2 years old.	[184]
ELEMENT Mexico N = 249	Phenols and Phthalates	BPA, MBP, MBzP, MCPP, MEP, MiBP, MEHP, MECPP, MEHHP, MEOHP, ΣDEHP, ΣHMW, ΣLMW	Urine N= 1 third trimester	One single visit between 8 and 14 years old BMI z-score, WC, and the sum of skinfolds	Uni-pollutant, linear regression	Yes, all *p*-values of sex interaction > 0.2	Only MBzP was negatively associated with the BMI z-score.	[177]
SEPAGES cohort France N = 484 pregnant women Recruitment: 2014–2017	Phenols, Parabens, and Phthalates	4 phenols, 4 parabens, 7 phthalates, and 1 non-phthalate plasticizer	Weekly pooled urine samples collected from the mother during T2 and T3 trimesters	BMI, height, weight, and head circumference at 3 and 36 months of age	Associations with individual chemicals using adjusted linear regression and mixtures of chemicals using a Bayesian kernel machine regression model	Yes	BP at T2: ↓ BMI and weight at 36 months of age. BP at T3: ↓ height at 36 months of age. BPS at T2: ↑ BMI, weight, and height at 3 and 36 months of age (tendance). BPS at T3: ↑ BMI, weight, and head circumference at 3 months of age (tendance). ΣDEHP at T3: ↑ BMI and weight at 3 months of age. ΣDiNP at T2: stratification for child sex, ↑ of head circumference in males, and ↓ in females at 3 and 36 months of age.	[175]

BBP: benzyl butyl phthalate; n-BuP: n-butylparaben; BP-3: benzophenone-3; BPA: bisphenol A; BzP: benzylparaben; DBP: di-n-butyl phthalate; DEHP: di-(2-ethylhexyl) phthalate; DEP: diethyl phthalate; DiBP: diisobutyl phthalate; DiDP: di-isodecyl phthalate; DiNP: di-isononyl phthalate; EtP: ethylparaben; HMW: high-molecular-weight phthalates; LMW: low-molecular-weight phthalates; MBP: mono-n-butyl phthalate; MBzP: monobenzyl phthalate; MCIOP: mono-(4-methyl-7-carboxyheptyl) phthalate; MCMHP: mono [2-(carboxymethyl)hexyl] phthalate; MCOP: mono-carboxy-iso-octyl phthalate; MCPP: mono(3-carboxypropyl) phthalate; MECPP: mono-(2-ethyl-5-carboxypentyl) phthalate; MECPTP: mono-2-ethyl-5-carboxypentyl terephthalate; MEHHP: mono(2-ethyl-5-hydroxyhexyl) phthalate; MEHP: mono(2-ethyl-hexyl) phthalate; MEOHP: (mono(2-ethyl-5-oxohexyl) phthalate; MEP: mono-ethyl phthalate; MetS: risk of metabolic syndrome; MiBP: mono-iso-butyl phthalate; MiDP: mono-iso-decyl phthalate; MiNP: mono-iso-nonyl phthalate; cx-MINP: mono-carboxy-iso-nonyl phthalate; MMP: monomethyl phthalate; MNP: mono-isononyl phthalate; MeP: methylparaben; OH-MiNP: mono-hydroxy-iso-nonyl phthalate; oxo-MiNP: mono-oxo-iso-nonyl phthalate; PrP: propylparaben. Arrows indicate the direction of the effect: a downward arrow (↓) indicates a decrease and an upward arrow (↑) indicates an increase.

#### 6.1.2. Maternal Exposure to PPPs and Metabolic Outcomes in Experimental Models (Table 4)

Studies of exposure to PPPs were initially interested in the risk of allergies in offspring, and also on the risk of mammary and genital carcinogenesis [207]. These will not be addressed in this review. These studies then focused either on the reproductive toxicity for the offspring, discussed later, or on the neurodevelopmental effects and their repercussions on behavior and learning. Both of these subjects were reported in a very recent review (for the bisphenols and phthalates) [208]. Finally, more recently, studies have focused on the metabolic effects of these PPP exposures and the repercussions on bodyweight, which will be the subject developed here.

Maternal oral exposure to bisphenol during the perinatal period, either A, S, or F, resulted in dose-grading adverse effects in female offspring. These adverse effects on the intestinal and systemic immune response depend on the bisphenol nature, i.e., A, S, or F. In mice, stronger impacts on inflammatory markers in feces were observed with BPS at the dose of 0.005 mg/kg bw/d. Exposure to BPA and BPF at low doses induced significant immune response changes in the offspring. These changes led to both intestinal and systemic Th1/Th17 inflammation [209]. Additionally, maternal exposure to BPS resulted in adverse effects on the triacylglycerol (increase in males), hormone levels (increase in T3 males and T4 females), and behavior (decreased food intake) of the offspring. These effects were observed as a function of the dose with a non-monotonic response [210]. Maternal exposure to BPA induced lighter weaning weights in some males. These males experienced rapid catch-up growth immediately after weaning [211]. So, they represent a subpopulation sensitive and vulnerable to very low fetal serum concentrations of BPA in the pg/mL range, particularly in the case of glucose intolerance. Gestational exposure to BPA has been shown to upregulate offspring pancreatic β-cell division and mass in an ERβ-dependent manner in adult male mice [212]. In C57BL/6J, maternal exposure to BPA mimicking human exposure levels (from 0.010 to 10 mg/kg bw/day) led to dose-specific effects on pancreatic islets in both the first (F1) and second generations (F2) in males only. Moreover, an increase in bodyweight was observed only in the F3 males. In addition, the lowest dose reduced the β-cell mass and smaller islets associated with increased insulin secretion, without a change in glucose tolerance. However, changes in the cytokine levels were reported across the generations in males [213]. In a transgenerational context, female offspring from males exposed in utero and during lactation to BPA were shown to exhibit impaired glucose tolerance despite the absence of compromised insulin sensitivity in vivo or reduced ex vivo glucose-stimulated insulin secretion. However, male offspring showed normal glucose tolerance [214]. Prenatal exposure to BPA (5 mg/kg bw/day) disrupted hepatic lipid homeostasis in a sex- and age-dependent manner. These effects, investigated by lipidomic and transcriptomic approaches, were marked around weaning (the accumulation of lipids and inflammation of the liver), but tended to fade with age, especially in females (bodyweight and total lipid content) [215,216].

In sheep, prenatal exposure to BPA (0–0.05–0.5–5 mg/kg bw/day by subcutaneous injections from days GD30 to GD90; term: 147 days) induced peripheral insulin resistance and adipose tissue disruptions in female offspring of 21 months of age [217]. In addition, lipotoxicity (an increase in blood and tissue triglycerides), accompanied by an increase in oxidative stress, was observed in a non-monotonic manner. In parallel, a reduction in antioxidants was shown in both liver and skeletal muscles, as well as altered proinflammatory markers in the liver (an increase in TNF-α and CD68, but IL-6 and IL-1B) and skeletal muscle (an increase in IL-6, IL-1B, CCL2, and CD38) [218]. These effects contributed to the resistance to insulin. These BPA-induced prenatal metabolic dysfunctions were corroborated for the middle dose (0.5 mg/kg/day) by transcriptomic analyses. These data provided mechanistic clues to explain oxidative stress and lipid accumulation, and potential mitochondrial and fibrotic defects in these tissues [219]. In the same animal model, prenatal exposure to BPA (0.05, 0.5, or 5 mg/kg/day) by subcutaneous injections from days GD30 to GD90 induced a trend toward decreased insulin and β-cell counts associated with an increase in glucagon and α-cell counts. The results were most consistent at the lowest BPA dose in fetal pancreata at GD90 and in adult offspring. These data suggest that early-life BPA exposure poses a likely threat to metabolic health [220].

In mice, maternal subcutaneous exposure to BPA (0.010 or 0.100 mg/kg bw/day) from GD9 to GD16 impaired pancreatic function, with a decrease in the mass of β-cell [221] and impaired glucose homeostasis in 6-month-old male offspring, but not in females [222]. The same replicated protocol showed that such maternal exposure of PND30 increased the retinoid concentrations and gene expression of key elements involved in the retinoid system in the liver in male offspring [223].

In mice, maternal exposure to BPA throughout gestation and lactation (1000 nM via drinking water) increased the cytokine levels in the spleens of the PND21 and PND42 offspring. The levels of cytokines were derived from Th17 cells (IL-17 and IL-21) and were essential for the differentiation of Th17 cells (IL-6 and IL-23). These increases were more pronounced in females and started at the lowest dose of BPA (100 nM), promoting inflammation induced in offspring [224]. When the mice were orally exposed to BPA (0.000050, 0.050, or 50 mg/kg diet) throughout gestation and lactation, primary bone marrow-derived mast cells (BMMCs) presented, after activation, increased cysteinyl leukotriene and TNFα production in all of the exposed groups [225]. Additionally, an increase in prostaglandin D2 and IL-13 production was observed only in the most BPA-exposed group. These BMMCs were generated from the bone marrow culture extracted from the femurs of PND21 progeny [225]. This production of proinflammatory mediators is generally associated with asthma, another harmful effect linked to the exposure of BPA. In mice exposed to BPA (0.010 or 10 mg/kg bw/d) throughout gestation and lactation, males exposed to the lowest dose were lighter at birth. These males then underwent rapid catch-up growth until weaning and showed increased bodyweight after PND117. Furthermore, glucose intolerance and insulin resistance resulted in increased body fat in offspring exposed to the higher dose [226]. Another study exposing the dam to BPA (0–30 mg/kg bw/day) for the same periods as above showed dose-dependent increases in body and liver weights in 20-week-old offspring [227]. A dose-dependent decrease in circulating glucagon in male offspring was also observed. However, in this study, females showed a dose-dependent decrease in body, liver, muscle, and fat pad weights. The latest effects in females were accompanied by decreased adipocyte size, serum lipids, serum leptin, and adiponectin, in parallel with increased physical activity [227]. Maternal exposure to BPA through drinking water (10 mg/L) induced insulitis, i.e., a pancreatic disease caused by lymphocyte infiltration, and accelerated the prevalence of diabetes in the 20-week-old female offspring [228]. In mice, perinatal exposure to BPA via the maternal diet (0.000050, 0.050, or 50 mg BPA/kg diet) induced an increase in the energy expenditure of the offspring during life until the age of 10 months. In female offspring, BPA exposure tended to decrease bodyweight and fats, with improved glucose and insulin profiles, at the highest dose, leading to a hyperactive and lean phenotype [229].

Maternal exposure of mice to the antibacterial TCS (8 mg/kg bw/d) from GD6 to GD14 has been shown to alter prenatal and postnatal growth and development, as well as metabolic phenotypes in male and female offspring. Compared with control offspring, TCS offspring (male or female) initially showed reduced bodyweight at birth, but then showed more rapid bodyweight gain during the fifth day of gestation, which increased over time [160]. Indeed, PND30 overweight TCS offspring showed, at PND60, increased visceral fat and adipocyte size, with delayed glucose clearance and insulin resistance. In rats, maternal oral exposure to TCS (1 mg/kg/day from GD14 to PND20 during lactation) increased bodyweight, blood glucose, and cholesterol in 5-month-old offspring, as well as food intake [230]. The increase in the latter is probably due to the increased hypothalamic expression of orexigenic neuropeptides. Moreover, in aged rats with high in utero exposure to TCS, a decreased hepatic glycogen content was observed, while the serum and hepatic triglyceride content increased with the upregulation of genes involved in carbohydrate and lipid metabolism pathways in the liver [231]. This was also observed in mice, along with an increase in serum and liver triglycerides with the increased gene expression of a protein involved in fatty acid synthesis, but the decreased gene expression of a protein involved in fatty acid oxidation [232].

What has been shown for any of these pollutants, depending on the dose studied, the route of exposure, the group of offspring considered (male or female), their age at the time of analysis, in addition to the animal model and species, must be rethought when it comes to combining these pollutants, as all of the cards of effects have to be reshuffled. The study of cocktail effects is likely to yield surprising results, like those investigated in a few studies [233], with a reduction in live weight for all of the mixtures in females regardless of the dose, and in males only at mid-dose.

Maternal n-BuP (parabens) exposure in mice was shown to induce a higher food intake and weight gain in female offspring only, probably reducing hypothalamic POMC expression induced by epigenetic modification [234].

According to the literature, early-life exposure to DEHP is potentially associated with increased adiposity in rodents [235,236]. Perinatal exposure to different phthalates will cause different metabolic outcomes in mice, with sex-specific responses. In mice, females exposed in utero to DEHP exhibited increased body fat and decreased lean mass, whereas exposure to DiNP induced only a decrease in glucose tolerance. In contrast, prenatal exposure of males to phthalate did not lead to any significant differences in the measured metabolic outcomes [237]. However, other studies in mice have reported that prenatal exposure to DEHP at a low dose (0.2 mg/kg/day) led to a metabolic syndrome in male offspring, including abnormal adipogenesis, energy expenditure, and glucose metabolism, by the deregulation of hepatic thiamine transport enzymes [238]. In the mouse liver, perinatal exposures to phthalates were associated with the short- and long-term activation of PPAR target genes, which was manifested by increased fatty acid production in early postnatal life and increased fatty acid oxidation in adulthood [239]. A similar accumulation of hepatic lipids was observed in rats exposed to DEHP during the perinatal period [240], as described for TCS [231,241]. Additionally, maternal exposure to DEHP in rats (0.75 mg/kg bw/day from GD6 to PND21) decreased serum insulin and triglyceride levels in PND70 male offspring. These outcomes were linked by the authors to the elevated expression of PPARγ (mRNA and protein) in white adipose tissue [242]. At PND21, male and female offspring pups exposed in utero to DEHP (700 mg/kg bw/day during the last third of gestation and lactation) and DBP (500 mg/kg bw/day) showed increased fasting glucose levels, as well as metabolic alterations [243]. Indeed, gestational exposure to DEHP has been shown to promote β-cell dysfunction and whole-body glucometabolic abnormalities in F1 offspring by downregulating the expression of critical genes involved in β-cell development and function [244]. Maternal exposure to a lower dose of DEHP (10 and 100 mg/kg bw/day) from GD9 to PND21 (lactation period) by oral gavage in the male offspring induced hyperglycemia, impaired tolerance to glucose and insulin, as well as hyperinsulinemia at PND80. This phenotype occurs because the levels of insulin signaling molecules such as insulin receptors, IRS1, Akt, and its phosphorylated forms are reduced [245]. In addition, maternal exposure to DEHP throughout gestation has been shown to disrupt thyroid function in offspring pups. DEHP disrupts thyroid function by damaging thyroid follicles and affecting thyroid transcription factor 1 (TTF-1), paired box 8 (PAX8), sodium iodide symporter (NIS), and thyroid peroxidase (TPO) both at the transcriptional level and at the protein level. In pups, this damage leads to a reduction in total thyroxine (T4) and an increase in thyroid-stimulating hormone (TSH) [246]. In adults, this damage was observed without an altered macro-index such as bodyweight in males at 14 weeks. The males were then likely to develop insulin resistance (hyperinsulinemia), oxidative stress (increased CAT catalase), and hypothyroidism (decreased T4) [247].

Prenatal exposure to low doses of DEHP or other phthalates has resulted in life-long metabolic consequences in a sex-dependent manner in offspring. Most of the time, the results are contradictory, with effects only in females or males. This exposure suggests a potential risk factor for later obesity and metabolic syndrome development in adulthood. Additionally, most studies on perinatal exposure to phthalates introduce bias to the extent that they focus only on the effects on male offspring in rodents, and only on female offspring in sheep.

As we can see in Table 4, the experiments relating to maternal oral exposure to phenols, parabens, and/or phthalates during the perinatal period (all or part of gestation and/or lactation) have studied very varied doses of the order of mg/kg with non-monotonic dose–responses and sex-specific metabolic effects in offspring. The observed effects are often transitory around weaning and deserve to be studied later in adulthood.

**Table 4:** Synthesis of the offspring outcomes related to maternal oral exposure to phenols, phthalates, and/or parabens during the perinatal period (all or part of gestation and/or lactation) according to the animal model, the chemicals, the dose administrated, the exposure route and duration.

Animal Model	Chemicals	Dose Administered	Exposure Route	Exposure Duration	Observation Stage	Function Studied	Metabolic Postnatal Outcomes	Additional Outcomes	Reference
Mouse	BPA	0.01, 10 mg/kg bw/d	Oral route (food)	Paternal exposure (12 weeks from 5 weeks of age)	0–20 weeks F1	Metabolism	Glucose tolerance and body composition: no effect Glucose tolerance at 4 and 7 months of age: BPA ↓ (♀)	Insulin-dependent glucose disposal in post-pubertal: BPA ↑ (0.01) Glucose tolerance (6 months) and glucose disposal (1 year): BPA ↓ Glucose accumulation: BPA ↑	[214]
Mouse	BPA	500 mg/kg bw/d	Oral administration	GD8-GD14	PND56	Reproduction/fertility	Mortality at birth: BPA ↑ Serum testosterone, FSH, and LH level: BPA ↓ (PND56) Serum estradiol level: BPA ↑ (PND56) Bax protein expression in Leydig cells, ovaries, and testis: BPA ↑ Ovaries and testis Bcl-2 protein expression: BPA ↓	Number of mature spermatozoids: BPA ↓ (♂) Number of granular cells: BPA ↓ (♀)	[248]
Mouse	BPA	0.01, 0.1 mg/kg bw/d	Subcutaneous injections	GD9-16	PND1-21 + 6 months	Glucose homeostasis	Bodyweight at birth and PND21: BPA ↑ (0.01)/↓ (0.1) Bodyweight at 3 months of age: BPA ↓ (♀) Insulin sensitivity and glucose tolerance: BPA ↓ (♂: 6 months of age) Serum insulin level: BPA ↑ (♂) Serum glycerol level: BPA ↑ (♂: 0.1) Glucose-stimulated insulin secretion and islets insulin secretion: BPA ↑ (0.01) Global intracellular calcium entry after glucose stimulation: BPA ↑ Pancreatic β-cell area: no effect Pancreatic β-cell proliferation: BPA ↓ (♂)	Litter size: no effect Maternal glucose intolerance and total mean area under the curve of glucose tolerance: BPA ↑ (0.01) Akt phosphorylation in the maternal liver after insulin stimulation: BPA ↓ (0.01) Insulin and TG serum level: BPA↑ Plasma glycerol and leptin level: BPA ↑ Maternal bodyweight 3–4 months after delivery: BPA ↑ Food intake: no effect	[222]
Mouse	BPA	5 mg/kg bw/d	Oral gavage	GD1-20	PND5-3 weeks of age	Metabolism	Bodyweight: BPA ↓ Serum lipid parameters: BPA ↓ (♂) Serum glucose level: BPA ↑ (♂) Liver number of differentially expressed genes (DEGs): 855 Liver *Cyp51* (sterol 14-α demethylase) expression (DEG common): BPA ↑ GSEA-enriched pathways: lipid metabolism (lipid transport/fatty acid metabolism/cholesterol biosynthesis)/energy metabolism (biological oxidation/tricarboxylic acid cycle) Liver peroxisome proliferator-activated receptor (PPAR) signaling and arachidonic acid pathways enrichment: BPA ↓ Liver number of differentially methylated CpGs (DMCs) in males: 476 Number of transcription factor (TFs) differentially expressed in the liver: 14 (*Esr1*, *Esrra*, *Hnfl1a*, *Pparg*, *Tcf21*, and *Srebf1*) Liver differentially expressed transcription factor involvement: estrogen and PPAR signaling	Number of differentially expressed genes (DEGs): 86 (adipose tissue)/93 (hypothalamus) *Cyp51* expression (DEG common): BPA ↑ (hypothalamus)/↓ (adipose tissue) Number of DMCs in males: 5136 (adipose tissue)/104 (hypothalamus) Top DMC-enriched processes: intracellular and extracellular communication and signaling-related pathways	[249]
Mouse	BPA	0.01, 10 mg/kg bw/d	Oral route (food)	GD-14 to PND21	PND7, 14, and 21 weeks of age	Metabolism	F2 and F3 bodyweight: no effect Obesity phenotype: BPA ↑ (♂ F2, F3) Body composition: no effect (♀) Glucose tolerance and insulin sensitivity: no effect (♂ F3) Glucose-stimulated insulin secretion: BPA ↑ (♂ F3: 0.01) β-cell mass and proliferation: BPA ↓ (♂ F3: 0.01)	Proinflammatory cytokine levels: BPA ↓ (IL-1β and IL-12p70) Immunostaining of CD3 (T lymphocyte markers) and F4/80 (macrophage markers) in the pancreas: BPA ↑ (♂ F3) Transforming growth factor-beta 1 (TGF-β)1 level: BPA ↑ (10) F2 maternal metabolic milieu: no effect	[213]
Mouse	BPA	0.1, 1, or 10 mg/L	Oral route (drinking water)	GD0-PND21	7 to 28 weeks of age	Metabolism	Mean insulitis grade and number of diabetic mice: BPA ↑ (10) Offspring’s number, fetal bodyweight, and sex ratio: no effect Number of regulatory T cells: BPA ↑ (♀: 10) Number of F4/80-positive tissue-resident macrophages in pancreatic islets: BPA ↓ (♀: 10)	Activated caspase-3-positive, insulin-positive, and glucagon-positive apoptotic cells in pancreatic islets: BPA ↑ Number of apoptotic cells: BPA ↑ LPS-induced interleukin secretion from splenocytes: BPA ↑ (10) IL-2 secretion: BPA ↓ (1, 10)	[228]
Mouse	BPA	0.01 mg/kg bw/d	Subcutaneous injections	GD9-16	PND0 and 30	Pancreatic function	Pancreatic β-cell area, mass, and proliferation: BPA ↑ (♀)		[212]
Mouse	BPA	0.01, 0.1 mg/kg bw/d	Subcutaneous injections	GD9-16	PND30	Retinoid signaling pathway	ATRA (all-trans-retinoic-acid) hepatic concentration: BPA ↑ (0.01) Expression of genes involved in ATRA biosynthesis: BPA ↑ (0.01: *Adh1*, *Aox1*, and *Cyp1a2*) Expression of genes involved in storage and metabolization of retinoids and ATRA biotransformation: no effect Expression of genes involved in the disposition of retinoid metabolites: BPA ↑ (0.1: *Bcrp*)/↓ (*Mrp3*) Expression of nuclear receptors modulated retinoid-dependant signaling in hepatic cells: BPA ↑ (0.01: Fgf21)/↓ (0.1: Rxr-β)		[223]
Mouse	BPA	0.01, 0.1 mg/kg bw/d	Subcutaneous injections	GD9-16	PND0, 21, 30, and 120	Pancreatic function	Bodyweight: BPA ↓ (PND0/PND21: 0.1)/↑ (PND21-30) Bodyweight gain: BPA ↓ (PND0-21)/↑ (PND21-30) Non-fasting plasma insulin, plasma leptin, and C-peptide level: BPA ↑ Insulin release: BPA ↑ Glucose-stimulated insulin secretion and pancreatic insulin content: BPA ↓ (0.01) Number and expression of DEGs in the islets of Langerhans: BPA ↑ (325)/↓ (330) Pancreatic β-cell mass: BPA ↑ (PND0/PND21/PND30)/↓ (0.1: 4 months) Pancreatic percentage of β-cell area: BPA ↑ (PND30: 10) β-cell proliferation and apoptosis: BPA↑ (PND30)	DEGs involvement: cell cycle/mitosis/cell division	[221]
Mouse	BPA	10, 100, or 1000 nM	Oral route (drinking water)	GD0-PND21	PND21, 42	Spleen immune response	Litter size, birth weight, survival rate, and sex ratio: no effect Weaning weight: BPA ↑ (PND21: 10)/↑ (PND35: 1000) T helper 17 cell (Th17) cell frequency in spleen: BPA ↑ (♀: 100, 1000/♂: 1000) *RORγt* expression: BPA ↑ (♀: 100, 1000/♂: 1000) IL-17 and IL-21 production level by Th17 cells: BPA ↑ Serum IL-6 and IL-23 levels: BPA ↑ Serum TGF-β level: no effect	Gestational weight: no effect	[224]
Mouse	BPA	0.00005, 0.05, or 50 mg/kg food/d	Oral route (food)	GD-14 to PND21	6 months of age	Inflammatory mediators/asthma	Bone marrow-derived mass cell (BMMC) cysteinyl leukotriene secretion: BPA ↑ BMMC prostaglandin D2 production: ↑ (50) BMMC tumor necrosis factor-alpha (TNFα) secretion: BPA ↑ IL-13 level: BPA ↑ (50) IL-4, IL-5, and IL-6 levels and histamine release: no effect BMMC DNA methylation level: BPA ↓ (50)	-	[225]
Mouse	BPA	0.01, 10 mg/kg bw/d	Oral route (food)	GD-14 to PND21	GD9.5-10.5, GD16.5-17.5, PND1, 14, 21, 28, and 98-117	Metabolism	Fetal bodyweight at PND1, 14, and 21: BPA ↓ (♂: 0.01) Fetal bodyweight at PND28 and food intake: no effect Bodyweight between PND98 and 117: BPA ↑ (♂: 0.01) Body fat content between PND98 and PND 117: BPA ↑ (♂) Bone mineral density and content: BPA ↓ (♂: 10) Insulin level: BPA ↑ (♂: 0.01) Glucose intolerance and basal rate of insulin release: BPA ↑ (♂: 10) Maximal glucose-stimulated insulin release: BPA ↓ (♂: 0.01)	F2 total insulin-like growth factor 2 (*Igf2*) mRNA expression: BPA ↑ (10) Bodyweight and fat: no effect Glucose intolerance: BPA ↑ (♂: 10) Insulin secretion: no effect Body fat content at PND98 to 117: BPA ↑ (♂: 10) Islets glucose-stimulated insulin secretion: BPA ↓ (0.01)	[226]
Mouse	BPA	0.006, 0.06, or 0.6 mg/kg bw/d	Subcutaneous capsule	GD9 to at least 3 weeks	PND2, 21, and 3 to 12 weeks of age	Glucose metabolism	Bodyweight at weaning and 5 weeks: BPA ↓ (♂: 0.006) Bodyweight gain between 3 and 5 weeks: BPA ↑ (♂: 0.006)/↓ (♂: 0.06) Bodyweight gain between 5 and 12 weeks: BPA ↑ (♂: 0.6) Bodyweight at 12 weeks and food intake: no effect Glucose intolerance: BPA ↑ Blood glucose level: BPA ↓ (♂: 0.6) Percent change relative to baseline in blood glucose level 30 min after glucose injection (GTT): BPA ↑ (♂: 0.06/0.6) Percent change relative to baseline in blood glucose level 60 min after GTT: BPA ↑ (♂)		[211]
Mouse	BPA	0.003, 0.01, 0.03, 0.1, 0.3, 1, or 3 mg/kg bw/d	Oral route (food)	GD-14 to PND21	PND21, and 5 to 23 weeks of age	Metabolism	Litter size, sex ratio, and survival rate: no effect Bodyweight: BPA ↑ (♂ from 6 weeks)/↓ (♀ 8 weeks) Bodyweight gain, body length, femur length and weight, and relative liver weight: no effect Glucose tolerance: no effect Liver weight: BPA ↑ (♂)/↓ (♀) Interscapular, perigonadal, perirenal, and caudal subcutaneous fat pad weights: BPA ↓ (♀) Perirenal white adipose tissue adipocyte size: BPA ↑ (♂)/↓ (♀) Histopathological liver examination: no effect Circulating glucagon level: BPA ↓ Insulin level: no effect Adiponectin, leptin, free fatty acid, and TG levels: BPA ↓ (♀)	Mortality: no effect Dams bodyweight, bodyweight gain, and food consumption: no effect Brown adipose tissue ucp1 expression: BPA ↑ (♀) Muscle weight: BPA ↓ (♀) Histopathological quadriceps femoris muscle and thyroid gland examination: no effect Histopathological pancreatic islets and adrenal examination: no effect	[227]
Mouse	BPA	0.00005, 0.05, or 50 mg/kg diet	Oral route (food)	GD-14 to PND21	3, 6, 9, and 10 months of age	Metabolism	Litter size, survival rate and sex ratio: no effect Fetal wean weight: BPA ↓ (0.00005) Food intake: BPA ↑ (♀ 6 months) Bodyweight and body fat mass: BPA ↑ (♀ 6 months: 0.05) Mean baseline glucose and insulin level: BPA ↓ (♀: 50) Mean adiponectin level: BPA ↑ (♀: 0.05)	Oxygen consumption: BPA ↑ (♀ 3 months: 50/♀ 6 months: 0.05, 50/♀ 9 months: 0.00005/♂ 9 months: 0.05, 50) Oxygen consumption corrected for lean body mass: BPA ↑ Carbon dioxide production level: BPA ↑ (♀ 9 months: 0.00005/♀ 6 months: 50/♂ 3 months: 0.05, 50) Respiratory exchange ratio: no effect	[229]
Rat	BPA	0.05 or 5 mg/kg bw/d	Oral gavage	GD3 to GD18	PND21 and PND60	Lipid metabolism	Total lipid content: BPA ↓ (♀ PND21: 5) Lipid accumulation and TG level: BPA ↓ (♀ PND21) PLS-DA analysis: separations of control, low-BPA-, and high-BPA-dose exposed groups Changes in several lipid classes: BPA ↑ (FA, acylcarnitine, cholesterol ester, monoacylglycerol, TG, monogalactosyl diacylglycerol, sphingomyelin, cardiolipin, phosphatidylserine, phosphatidylglycerol, and sulfatide) Total free fatty acid and acylcarnitine level: BPA ↑ (PND21) Level of total monoacylglycerol: BPA↑ (♂ PND21/♀ PND21: 0.05) Level of total cholesterol ester: BPA ↑ (♀ PN21: 5) Lipogenesis: BPA ↑ (♀ PND1)/↓ (♂ PND1 to PND21) Ceramide and phosphatidylcholine level: BPA ↓ (♀ PND21) Cardiolipin level: BPA ↓ (♀ PND21)/↑ (♂ PND21: 0.05) Sulfatide level: BPA ↑ (♀ PND21 and PND60) Total sphingomyelin, phosphatidylglycerol level: BPA ↑ (♂ PND21) Digalactosyl and sulfoquinovosyl diacylglycerol level: BPA ↑ (♀ PND60) Phosphatidylethanolamine level: BPA ↓ (♂ PND60) *Gpd1* gene expression involved in glycerophospholipid metabolism: BPA ↓ (♀) *Gnpat* gene expression involved in glycerophospholipid metabolism: BPA ↓ (♂) Bodyweight: BPA ↑ (♀ PND1)	PUFAs: BPA ↑ (PND21) MUFAs (C15:1 and C18:1): BPA ↑ (♂ PND21) Saturated very-long-chain FAs (C32:0 and C34:0): BPA↓ (♂ PND21: 0.05/♀ PND21: 5) Unsaturated very-long-chain FAs (C36:4): BPA ↓ (♀ PND21) Potential biomarkers for prenatal BPA exposure: ♀ (8 genes, 9 FAs, 1 cholesterol ester, 19 TG, 3 diacylglyceryl-trimethylhomoserine, 1 ceramide, and 1 phosphatidylcholine)/♂ (30 genes, 1 FA, 1 TG, 1 phosphatidylmethanol, 2 phosphatidylcholine, and 4 phosphatidylethanolamine)	[215]
Rat	BPA	0.05 or 5 mg/kg bw/d	Oral gavage	GD3-18	PND1, 13, 21, 30, and 60	Liver metabolism	Bodyweight: BPA ↑ (♀ PDN1-PND60/♂ PND13, PND21-PND30/♂ PND60: 5) Liver weight: BPA ↑ (♀ PND21)/↓ (♂ PND21) Relative spleen weight: BPA ↑ (♀ PND1) Relative heart weight: BPA ↑ (♀ PND1)/↓ (♀ PND60) Relative kidney weight: BPA ↓ (♀ PND60) Number of DEGs: 1239 (♀ PND1: 0.05)/1672 (♀ PND1: 5)/1250 (♂ PND1: 0.05)/722 (♂ PND1: 5)/217 (♀ PND21: 0.05)/337 (♀ PND21: 5)/326 (♂ PND21: 0.05)/443 (♂: PND21: 5) Principal component analysis: a clear distinction between exposed and control PDN1 males, between exposed and control PND1 females, between exposed and control groups, between high-BPA exposed and control PDN21 females, between exposed and control PND21 males, and between PND21 males and females Number of differentially expressed proteins (DEPs): 101 (♀ PND1)/188 (♂ PND1, PND21: 0.05)/204 (♂ PND1: 5)/176 (♀ PND21: 0.05)/159 (♀ PND21: 5)/137 (♂ PND21: 5) Number of enriched pathways common from DEGs and DEPs in both sexes: 30 (♀ > ♂) Top pathways enriched: fatty acid degradation, steroid hormone biosynthesis, and PPAR signaling pathways Enriched diseases: fatty liver, diabetes, obesity, and cardiovascular diseases TC and cholesteryl ester levels: BPA ↑ (♀ PND60: 0.05) HDL cholesterol level: BPA ↑ (♀ PND60: 0.05/♂ PND60: 5) LDL cholesterol level: BPA ↓ (♂ PND60: 0.05)	Anogenital distance (AGD): BPA ↑ Anogenital index (AGI): BPA ↑ (♀ PND21)	[216]
Rat	BPA	0, 0.05, 0.5, or 5 mg/kg bw/d	Oral gavage	GD5-19	PND1, 21 and 56	Lipid metabolism	Bodyweight: BPA ↓ (♀ PND56: 0.5) Liver-to-bodyweight ratio: BPA ↑ (♀: 0.05/♀: 0.5) Serum TG and TC level: BPA ↑ TG liver level: BPA ↑ TC liver level: BPA ↑ (PND21) Liver fatty acid oxidation-related gene expression: BPA ↓ (PND21 P*PARα*/PND21 *CPT1α*: 5/PND56 *PPARα*: 0.5, 5) Liver fatty acid oxidation-related protein expression: BPA ↓ (PND21 *PPARα CPT1α*: 5/PND56 *PPARα* 0.5, 5/PND56 *CPT1α*: 5) Liver fatty acid synthesis-related gene expression: BPA ↑ (*SREBP-1*, *ACC1*, *FAS*, *SCD-1*) Liver fatty acid synthesis-related protein expression: BPA ↑ (*SREBP-1*, *SCD-1*)	Liver *mTOR* mRNA expression: BPA ↑ (PND21: 0.05/PND56: 5) Liver mTOR protein expression: BPA ↑ (PND21/PND56: 0.5, 5) Liver *CRTC2* mRNA expression: BPA ↑ (PND21/PND56: 0.05, 0.5) Liver CRTC2 phosphorylation level: BPA ↑ (0.5, 5)	[250]
Sheep	BPA	0.05, 0.5, or 5 mg/kg bw/d	Subcutaneous injections	GD30-90	GD68, 6, 14, 15, 19, 21 weeks, and 13 months of age	Metabolism	Fasting glucose level: BPA ↑ (6 weeks: 0.05) Cumulative insulin and insulin/glucose ratio responses: BPA ↑ (13 months: 0.5) Acute insulin response: BPA ↑ (0.5) Glucose tolerance: no effect (15 months) Bodyweight, total fat, visceral fat, and subcutaneous fat: no effect Visceral adipose tissue cell area and diameter: BPA ↑ (♀) Subcutaneous adipose tissue marker of macrophage infiltration CD68 expression: BPA ↑		[217]
Sheep	BPA	0.5 mg/kg bw/d	Subcutaneous injections	GD30 to GD100	21 months F1	Liver and muscle metabolic function	Number of differentially expressed (DEGs) genes in the liver: BPA ↓ (138)/↑ (56) Top 10 DEGs in the liver: BPA ↓ (*WFDC2*, *MSLN*, *MMP7*, *COLEC12,* and *SLC44A4*)/↑ (CCDC152 and three other genes with yet-to-be-identified roles, including thioredoxin-like protein 1, putative olfactory receptor 3A4, and elongation factor 1-beta-like) Number of enriched gene pathways in the muscle and liver: 157 Pathways enriched in the muscle and liver: mitochondrial, extracellular matrix-related, and oxidative phosphorylation pathways Enriched pathways in the liver: the response to oxidative stress, lipid biosynthetic process, endoplasmic reticulum, and Golgi apparatus structure and function Number of differentially expressed lncRNAs in the liver: BPA ↓ (49)/↑ (28) Number of differentially expressed miRNAs in the liver: BPA ↓ (6)/↑ (8) Number of differentially expressed snoRNAs in the liver: BPA ↓ (63)/↑ (64) Number of differentially expressed snRNAs in the liver: BPA ↓ (15)/BPA ↑ (40) Top 10 of miRNAs in liver: BPA ↑ (*MIR200B*, *MIR409*, *MIR125B*, *MIR543*, *MIR25*, *MIR22*, and *MIR191*)/↓ (*MIR26B* and *MIR154A*) Potential biomarkers of prenatal BPA impact in the liver: BPA ↓ (4 lncRNAs)/↑ (5 lncRNAs)	Number of differentially expressed genes in the muscle: BPA ↑ (80)/↓ (32) Top 10 dysregulated genes in muscle: BPA ↓ (multidrug resistance-associated protein 4-like genes, ATP-binding cassette subfamily C member 4-like gene, two uncharacterized genes, high mobility group protein 20A-like gene, and 40S ribosomal protein S3a pseudogene)/↑ (HBB) Pathways enriched in the muscle: RNA biosynthetic process, immune function, and collagen synthetic gene pathways Number differentially expressed lncRNAs in the muscle: BPA ↑ (6) Number of differentially expressed snoRNAs in the muscle: BPA ↓ (47)/↑ (18)	[219]
Sheep	BPA	0.05, 0.5, or 5 mg/kg bw/d	Subcutaneous injections	GD30-90	21 months F1	Metabolism	Liver IL-1β, IL-6, and chemokine (C-C) ligand 2 expression: BPA ↓ Liver CD68 (macrophage marker) expression: BPA ↑ Liver TNF-α expression: BPA ↑ (0.05) Visceral adipose tissue IL-1β expression: BPA ↑ Visceral adipose tissue CCL2 expression: BPA ↑ (5) Liver oxidative stress marker 3-nitrotyrosine level: BPA ↑ Visceral adipose tissue 3-nitrotyrosine level: BPA ↑ (5) Liver and visceral adipose tissue lipid peroxidation marker Thiobarbituric acid reactive substances (TBARS) level: BPA ↑ (5) Liver antioxidant (*GSR*) mRNA expression: BPA ↓ (0.5, 5) Liver antioxidant superoxide dismutases 1 (*SOD1*) mRNA expression: BPA ↓ (0.05, 0.5) Liver *SOD2* mRNA expression: BPA ↑ (0.05) Visceral adipose tissue glutathione reductase (*GSR*) and *Cyp19* mRNA expression: BPA ↑ (0.5, 5) Liver GSR activity: BPA ↑ (5) Plasma TG content and high-molecular-weight adiponectin level: no effect Hepatic TG content: BPA ↑ (0.5, 5) Plasma low-molecular-weight adiponectin level: BPA ↓ (5) Visceral adipose tissue *Cyp17* mRNA level: BPA ↑ (5) Visceral adipose tissue *AR* mRNA expression and CYP17 and estrogen receptor 1 (ESR1) protein level: no effect Visceral adipose tissue AR protein expression: BPA ↓ (5) Visceral adipose tissue estrogen receptor 2 (ESR2) protein level: BPA ↓ (0.5, 5) Visceral adipose tissue *Esr1* mRNA expression: BPA ↑	Skeletal muscle IL-6 expression: BPA ↑ Skeletal muscle CD38 expression: BPA ↑ (0.5, 5) Skeletal muscle IL1B expression: BPA ↑ (0.5) Skeletal muscle 3 nitrotyrosine and TBARS levels: BPA ↑ (0.05, 0.5) Skeletal muscle *SOD1* mRNA expression: BPA ↓ (5) Skeletal muscle *GSR* mRNA expression: BPA ↓ Skeletal muscle *SOD2* mRNA expression: BPA ↑ (0.05) Skeletal muscle GSR activity: BPA ↓ (0.5) Muscular TG content: BPA ↑ (0.5, 5)	[218]
Sheep	BPA	0.5 mg/kg bw/d	Subcutaneous injections	GD30 to GD100	21 months F1	Cardiovascular function	Lung, kidney, and adrenal weight: BPA ↓ Heart rate and blood pressure: no effect	Cardiac failure gene expression in the left ventricle: BPA ↑ (*ANP*, *COL1A1*) Cardiac failure gene expression in the right ventricle: BPA ↑ (*ANP*)/↓ (*COLA1*)	[251]
Sheep	BPA	0.05, 0.5, or 5 mg/kg bw/day	Subcutaneous injections	GD30-GD90	GD65, GD90, 21 months F1	Pancreas	Fetal weight: no effect (GD65, GD90) Pancreas weight: no effect (GD65, GD90) Pancreas/fetal weight: no effect (GD65, GD90) Pancreatic islet insulin: BPA 0.5 ↓ (GD90) Beta-cell size: no effect Beta-cell count: BPA 0.5 ↓ (GD90) Pancreatic islet glucagon: no effect (GD60, GD90), BPA 0.05 ↑ (adult F1) Alpha-cell size: BPA 0.5 ↑ (GD90), BPA 0.05 ↑ (adult F1) Alpha-cell count: BPA 0.5 ↑ (GD90) Alpha-to-Beta-cell count: BPA 0.5 ↑ (GD90), BPA 0.05 ↑ (adult F1) Islet collagen accumulation: 0.05 ↑ (adult F1) Pancreatic apoptosis: no effect (GD65, GD90, and adult F1) Gene expression of apoptotic and cell proliferation markers: no effect (GD65, GD90, and adult F1) Fibrosis gene expression: BPA ↓ ACT1 (GD65)		[220]
Rat	BPS	0.01, 0.05 mg/kg bw/d	Oral gavage	GD1-PND21	PND21, PND160-180	Metabolism, endocrine system	Plasma triacylglycerol and thyroxine (T4) level: BPS ↓ (♂ PND21: 0.05) Plasma25-hydroxyvitamin D (25(OH)D) level at PND21 and 180: BPS ↑ (♀: 0.05) Food intake: BPS ↓ PND180 visceral fat mass: BPS ↓ (♂) PND180 brown adipose tissue lipid droplets: BPS ↓ (650: ♀) Preference for high-fat diet over high-sugar diet: BPS ↑ Plasma triacylglycerol level: BPS ↑ (♂ PND180: 0.05) Plasma total triiodothyronine (T3) level: BPS ↓ (♂ PND180: 0.05) PND180 plasma free T4 level: BPS ↓ (♀)	Plasma testosterone level: BPS ↓ (0.05) Plasma progesterone level: BPS ↓ (♀ PND180: 0.01)	[210]
Mouse	BPA, BPS, BPF	0.005, 0.05 mg/kg bw/d	Oral route	GD15-PND21	PND70	Intestinal and systemic immune response	Bodyweight: BPF ↓ (0.05: PND70) Litter size: BPA ↓ (0.05)/BPS ↓ (0.005) Fecal immunoglobulin A (IgA) level: BPA ↓ (♀: 0.05) Lipocalin fecal level: BPS ↑ Plasmatic immunoglobulin G (IgG) level: BPA ↓ (♀)/BPS ↓ (♀)/BPF ↓ (♀) Total IgA level: no effect T helper 1 (Th1) subpopulation in the lamina propria: BPA ↑ (♀: 0.005) Th1 frequency in the spleen: BPA ↑ (♀: 0.05)/BPF ↑ (♀: 0.05) Intestinal Th17 frequency: BPA ↑ (0.05)/BPF ↑ (0.05) Small intestine *lamina propria* cell T cell receptor (TCR)-stimulated IL-17 secretion: BPA ↑ (0.05) T cell frequency in siLP and at systemic level: BPA ↓ (0.05) Principal component analysis: a higher dose BPA- and BPF-exposed groups were the most distant ones and were well separated from the control group/The higher dose BPS-exposed group was less distant to the control group/The high-dose BPA exposed group differed from the control group in terms of the IFN-γ and IL-17 levels and Treg frequency/BPF-exposed groups differed to the control in term of bodyweight and plasmatic IgA level	Plasma-specific anti-*E. coli* IgG: BPS ↑ (0.05) Plasma-specific anti-*E. coli* IgA: no effect	[209]
Mouse	BPA + NP, OP or IsoBP	5, 50, or 500 mg/kg bw/d	Oral gavage	GD1-21	PND1, and 21 and 41	Fetal development	Survival rate: BPA + OP ↓ (5) Sex ratio: BPA + OP ↓ (50)/BPA + IsoBP (5, 500) Bodyweight: BPA + OP ↓ (♀)/BPA + NP ↓ (♀)/BPA + IsoBP ↓ (♀) Pituitary and liver weight: BPA + OP ↑ (♀: 5, 500)/BPA + IsoBP ↑ (♀: 5, 50) Adrenal weight: BPA + OP ↑ (5, 500)/BPA + IsoBP ↑ (♀: 5, 50) Kidney weight: BPA + OP ↑ (♀: 5, 500)/BPA + IsoBP ↑ (♀: 5, 50)/BPA + IsoBP ↓ (♂: 5, 500)	Dams gestational day times: BPA + IsoBP ↓/BPA+ NP ↓ (50, 500)/BPA + OP (50, 500) Spleen and reproductive organs weight: no effect Thyroid weight: BPA + NP ↓ (♂)/BPA + OP ↓ (♂: 50)/BPA + IsoBP ↓ (♂: 5/50)	[233]
Rat	BPA, DEHP	5 µg/kg bw/d (BPA) and/or 5µg and 7.5 mg/kg bw/d (DEHP)	Oral administration	GD6-GD21	PND1, 7, 14, and 21, PND21 to PND84, PND112, and PND168	Fetal development/food intake	Maternal gestational weight gain: no effect Post-weaning bodyweights, and food and water intakes: no effect Heart weight: BPA + DEHP ↑ (♂: 7.5) Relative thymus weight: BPA ↓ (♂)/DEHP ↓ (♂: 7.5) Cortex apoptotic index: BPA + DEHP ↑ (7.5) Medulla apoptotic index: BPA + DEHP (♀: 7.5)	Abortion rate: BPA + DEHP ↑ Gestational index: DEHP ↓ (7.5)/BPA + DEHP ↓ Litter size and PND21 pup weight and sex ratio: no effect Crown-to-rump length: BPA ↑/BPA+ DEHP ↑ (5) Chest circumference: DEHP ↑ (5)/DEHP + BPA ↑ (5)/DEHP ↓ (7.5)/DEHP+ BPA ↓ (7.5)	[158]
Mouse	TCS	4, 8 mg/kg bw/d	Oral gavage	GD6-14	PND1, 30, and 60	Placenta/metabolism	Bodyweight gain: TCS ↑ (PND5, PND30, PND60: 8) Visceral fat-to-bodyweight ratio and adipocyte size: TCS ↑ (PND30: 8) Fasting plasma glucose and serum insulin level: TCS ↑ (PND60) Areas under the curve of glucose and insulin tolerance test: TCS ↑ (PND60) The phenotype of insulin resistance and hyperphagic obesity: TCS ↑ Cumulated food intake within 4 days: TCS ↑ (PND30) Food intakes normalized by bodyweight: TCS ↑ (PND30) Oxygen consumption: no effect		[160]
Rat	TCS	1 mg/kg food	Oral route (food)	GD14-PND20	PND1 to 8 months	Food intake/metabolism	Bodyweight: TCS ↓ (♂ 2 months)/↑ (♂ 4 months/♀ 8 months) Food intake: TCS ↑ (5 months) Liver weight and liver-to-bodyweight ratio: TCS ↑ (♂ 4 months) Serum cholesterol level and glucose concentration: TCS ↑ (8 months)	*Npy* and *Agrp* hypothalami mRNA expression (involved in the appetite regulatory network): TCS ↑ (♂)	[230]
Rat	TCS	10, 50 mg/kg bw/d	Oral gavage	GD0-PND21	3, 21, and 52 offspring’s weeks of age	Metabolism/microbiota	Glucose clearance: TCS ↓ (52 weeks: 50) Blood glucose level: TCS ↑ (21 weeks: 50/3 and 52 weeks: 10) Serum HDL cholesterol level: TCS ↑ (52 weeks/21 weeks: 50) Serum leptin level: TCS ↑ (52 weeks) TG and LDL cholesterol level: TCS ↑ (21 and 52 weeks) Hepatic glycogen level: TCS ↓ (52 weeks/21 weeks: 50) Number of DEGs: 709 (21 weeks: 10), 699 (52 weeks: 10), 473 (21 weeks: 50), and 1103 (52 weeks: 50) Involvement of DEGs: lipid metabolic process, fatty acid metabolic process, cellular glucuronidation, linoleic acid metabolism, glycolysis/gluconeogenesis, lipid and carbohydrate metabolism, arachidonic acid metabolism, and biosynthesis of unsaturated fatty acids	Alpha diversity of the microbial community in gut microbiota: TCS ↓ (50) Alpha diversity among gut microbiome and abundance of Bacteroides in the microbiota: TCS ↑ (50) Abundance of Verrucomicrobia and level of Akkermansia muciniphila in the microbiota: TCS ↓	[231]
Mouse	DEHP	10–12, 55–64, and 119–145 mg/kg bw/d	Oral route (food)		GD18	Fatty acid metabolism	α-linolenic acid level: DEHP ↑ (10–12) Palmitoleic acid level: DEHP ↑ (55–64, 119–145) Oleic acid level: DEHP ↑ (119–145) *Fads* mRNA expression (conversion EFA into LC-PUFA): DEHP ↑ (119–145: PPARα null mice)	Linoleic and oleic acid level: DEHP ↓ (55–64, 119–145) α-linolenic and eicosapentaenoic acid level: DEHP ↓ Palmitic acid level: DEHP ↓ (10–12, 55–64)	[252]
Mouse	DEHP	0.2, 2, or 20 mg/kg bw/d	Oral gavage	GD-7 to GD21	12 weeks of age	Metabolism	Bodyweight at 12 weeks of age: DEHP ↑ (♂: 0.2) ↑ (♂: 0.2) White adipocyte hypertrophy: DEHP ↑ (0.2) Plasma TC, TG, LDL, and HDL cholesterol levels: DEHP ↑ (0.2) Lipid deposition in liver cells at 12 weeks of age: DEHP ↑ (0.2) Number of liver DEGs: DEHP ↑ (♂: 0.2: (932))/↓ (♂: 0.2 (794)) DEGs involvement: metabolism-related pathways (fatty acid metabolism pathway) Hepatic thiamine and D-glucuronic acid level: DEHP ↓ (0.2) Hepatic glucose-6-phosphate and N-acethylglutamic acid level: DEHP ↑ (0.2) The area under the curve of the glucose tolerance test: DEHP ↑ (0.2) Plasmatic glucose level: DEHP ↑ (♂: 0.2) Hepatic *Slc2a2* expression involved in hepatic glucose secretion and hepatocyte blood glucose uptake: DEHP ↑ (0.2) Hepatic *Slc19a2* expression involved in thiamine transport: DEHP ↓ (0.2) Cumulative food intake: no effect Bodyweight gain at 12 weeks of age: DEHP ↑ (0.2)	Expression of thermogenic genes: DEHP ↓ (0.2 (*Ucp1*, *Cidea*, *Adrb3*)) Differentially generated metabolites involvement: ascorbate and aldarate metabolism, phenylalanine, tyrosine and tryptophane biosynthesis, and thiamine metabolism	[238]
Mouse	DEHP	25 mg/kg chow/d (5 mg/kg bw/d)	Oral route (food)	GD-14 to PND21	5 months of age	Cardiac DNA methylome	Mortality, litter size, sex ratio, bodyweight, and relative heart weight ratio: no effect Heart DMC and differentially methylated region (DMR): DEHP ↑ DEHP pathways enrichment about hypermethylated DMCs: receptor binding (♀)/neurotransmitter transport (♀)/smooth muscle differentiation (♀)/histone demethylation (♀)/insulin signaling (♀)/meiosis (♀)/glucose transport (♂) DMC and DMR mapped gene differentially methylated in human heart failure: DEHP ↑ Common genes involved in cardiac fibrosis and development or contribute to sex differences in ischemia–reperfusion injury: *PRKCE* (♀)/*SPRY1* (♀)/*GJA5* (♀)/*ECE1* (♀)/*SMAD7* (♂)/*DNMT3A* (♂)		[253]
Mouse	DEHP	250 mg/kg bw/d	Oral gavage	GD0-PND21	PND21	Skeletal muscle development	Bodyweight change: DEHP ↓ Ratios of quadriceps, gastrocnemius, and tibialis anterior muscle weights to tibia lengths: DEHP ↓ Average tibialis anterior myofiber cross-sectional areas: DEHP ↓ Muscle proteolytic marker (MuRF1 and atrogin 1) expression: DEHP ↑ Muscle myogenesis marker (MyoD and Myogenin) expression: DEHP ↓		[254]
Mousse	DEHP	30 mg/kg bw/d	Oral administration	GD-28 to PND28	8 weeks of age	Metabolism	Diastolic arterial, systolic, and mean blood pressure: DEHP ↑ Heart rate: no effect Bodyweight: DEHP ↑ White and brown adipose tissue-to-bodyweight ratio: DEHP ↑ White adipose tissue adipocyte size: DEHP ↑ Brown adipose tissue adipocyte number: DEHP ↑ Serum cholesterol level and liver cholesterol content: DEHP ↑ Serum TG and glucose level: no effect LDLR, SR-B1, CYP7A1, and ABCG5 hepatic protein expression: DEHP ↓ LRP-1 hepatic protein expression: no effect	Aorta phosphorylated eNOS^thr497^: DEHP ↑ Aorta phosphorylated eNOS^Ser1179^ and eNOS^Ser635^: DEHP ↓ Aorta AT1R protein expression: DEHP ↑ Aorta AT2R protein expression: no effect	[236]
Rat	DEHP	300, 600 mg/kg bw/d	Oral gavage	PND21 to 24 weeks (males)	25 weeks	Metabolism	Accumulation of DEHP in fat and serum: DEHP ↑ (long-term) Bodyweight changes: DEHP ↑ (24 weeks) TC, TG, ASAT, ALAT, and high-density lipoprotein levels: DEHP ↑ (24 weeks)) Liver-to-bodyweight ratio: DEHP ↑ (300) Lipid droplets in the form of subcircular vacuoles in the liver: DEHP ↑ Degenerative and necrotic tubular epithelial cells along with fat vacuoles in the liver: DEHP ↑ (600) Separated ellipses of the principal component analysis of the serum metabolic profiling between control and exposed groups Cell necrosis, and fatty and vacuole degeneration in the liver cytoplasm: DEHP ↑ *UDPGT* mRNA expression: DEHP ↓ *D1* mRNA expression: DEHP ↑		[255]
Rat	DEHP	0.75 mg/kg bw/d	Oral gavage	GD6-PND70	PND0-70	Lipid metabolism	Bodyweight: DEHP ↑ (GD6-PND21)/↓ (GD6-PND70) TG blood level: DEHP ↓ (GD6-PND21) ALAT blood level: DEHP ↑ (GD6-PND70) TG hepatic level: DEHP ↑ Small lipid droplets in hepatocytes: DEHP ↑ TG-related gene expression: DEHP ↑ (GD6-PND21: *AGPAT1*, *DGAT1*/GD6-PND70: *ACSL1*, *PNPLA2*, *MGAT1*, *DGAT1*, and *PPARα*)/↓ (GD6-PND21: *SREBP-1c* and *GPAT*) TG-related protein expression: DEHP ↑ (GD7-PND21 and GD6-PND70: *DGAT1*)/↓ (GD-PND70: *SREBP-1c*)		[240]
Rat	DEHP	300 mg/kg bw/d	Oral gavage	GD14-PND0	PND60, 200	Blood pressure/heart rate	Systolic blood pressure: DEHP ↓ (nighttime) Diastolic arterial pressure: DEHP ↓ (low-sodium diet) Heart rate: no effect Aldosterone level: DEHP ↓ (normal salt diet)	Locomotor activity during daytime: DEHP ↓ (PND60) Locomotor activity during night-time: DEHP ↓	[256]
Rat	DEHP	20, 50, 100, 300, and 750 mg/kg bw/d	Oral gavage	GD14-PND0	PND60	Metabolism	Circulating aldosterone concentration: DEHP ↓ (100, 300, 750)/↑ (♀: 300) Corticosterone and adrenocorticotropin hormone (ACTH) level: no effect Estradiol level: DEHP ↓ (♀: 300) Liver-made angiotensinogen (*Agt*) and renin (*Ren1*) mRNA level: no effect Adrenal morphology: no effect Adrenal weight: DEHP ↓ (750) Adrenal *AGTR1a* mRNA level: DEHP ↓ (300) Adrenal *AGTR1b* mRNA level: DEHP ↓ Adrenal *AGTR2* mRNA level: DEHP ↓ (750, 950) Kidney *AGTR2* mRNA level: no effect Adrenal and kidney *Agtrap* mRNA level: DEHP ↓ Adrenal AGTR1A and AGTR1B protein level: DEHP ↓ (300, 750) Adrenal aldosterone gene synthase (*Cyp11b2*) mRNA level: DEHP ↓ (500, 750) TG, TC, HDL, and LDL cholesterol levels: no effect Adrenal cholesterol uptake receptor (*LDLR*) mRNA level: DEHP ↑ Heart *LDLR* mRNA level: DEHP ↓ (300)/↑ (750) Adrenal HMG-CoA reductase and synthase (*Hmgcr* and *Hmgcs1*) and insulin-induced gene 1 (*Insig1*) mRNA level: DEHP ↑ Heart *Hmgcr*, *Hmgcs1*, and *Insig1* mRNA level: no effect Adrenal lipid-droplet accumulation: DEHP↑	Circulating testosterone concentration: DEHP ↓ (100, 300, 750) Sodium, potassium, chloride, calcium, and angiotensin II (AT II) level: no effect	[257]
Rat	DEHP	0.75 mg/kg bw/d	Oral administration	GD6-PND21 or/and PND22-70	PND70	Metabolism	Serum insulin and TG levels: DEHP ↓ (GD6-PND21) Serum leptin and adiponectin levels: no effect White adipose tissue *PPARγ* mRNA and protein expression levels: DEHP ↑		[242]
Rat	DEHP	10 or 100 mg/kg bw/d	Oral gavage	GD9-PND21	PND80	Glucose metabolism	Fasting blood glucose level and insulin resistance: DEHP ↑ Serum ASAT, ALAT, alkaline phosphatase, urea, and creatinine levels: DEHP ↑ Serum insulin concentration: DEHP ↑ Hepatic glycogen concentration and glucogen synthase activity: DEHP ↓ Molecules involved in insulin signaling in the liver: DEHP ↓ (Insulin receptor beta (IRβ), IRβ^Tyr1162^, insulin receptor substrate 1 (IRS1), IRS1^Tyr632^, β-Arrestin, Akt, Akt^Ser473^/100: c5rc protein, Akt^Thr308^) Targets of insulin signal transduction: DEHP ↑ (GSK3β, FoxO1)/↓ (GSK3β^Ser9^, FoxO1^Ser256^) Liver G-6-Pase and PEPCK (enzymes involved in gluconeogenesis) mRNA expression: DEHP ↑ Birth weight: DEHP ↓ (♂)	Serum testosterone and estradiol concentration: DEHP ↓	[245]
Rat	DEHP	1, 10, or 100 mg/kg bw/d	Oral gavage	GD9-21	PND60	Pancreatic function	Fasting blood glucose level: DEHP ↑ Serum insulin concentration: DEHP ↓ Glucose and insulin intolerance: DEHP ↑ Body and pancreatic wet weight: DEHP↓ Insulin autocrine action in endocrine pancreas: DEHP ↓ (PND60) Plasma membrane insulin receptor beta (InsRβ) protein level: DEHP ↓ IRS-2 protein level: DEHP ↓ Endocrine pancreas Akt protein level: DEHP ↓ (PND60) Islets ex vivo glucose-stimulated insulin secretion: DEHP ↓ Islets GLUT2 cytosolic and plasma membrane GLUT2 protein level: DEHP ↓ (10, 100) Glucokinase activity: DEHP ↓	Islets mRNA expression: DEHP ↑ (*FoxO1*, *Atf4*, *Atf6*, *Bip*, *DNMT3a*, *Cnmt3b*, *MeCP2*, *MDB2*, *mir143*, and *mir375*/10, 100: *DNMT1*)/↓ (10, 100: *GLUT2*) Insulin autocrine action in the endocrine pancreas: DEHP ↓ (PND60 pInsRβ^Tyr11162−1163^ protein level: DEHP ↓ pIRS^Tyr632^ protein level: DEHP ↓ (♂/♀: 10, 100) pIRS^Ser636/639^ protein level: DEHP ↑ pAkt^Ser473^ and pAkt^Thr308^ protein level: DEHP ↓ pAkt^Tyr315/316/312^ protein level: DEHP ↓ (10, 100) Islets protein level: DEHP ↑ (FoxO1)/↓ (MafA, Pdx1, Pax4 and Pax6 and HNF4-α)	[244]
Rat	DEHP	600 mg/kg bw/d	Intragastrically administration	GD0-PND21	8 and 14 weeks of age	Metabolism	Body and organ weight, body length, food intake, fat pad weight, and ratio: no effect Serum ALAT, TP, ALB, BUN, and creatinine level: ↓Blood lipids content: no effect Insulin level: DEHP ↑ Identification of biomarkers in several metabolic pathways: lipid metabolism pathway, and retinol metabolism pathway	Serum catalase level: ↓ Serum total antioxidant capability, SOD, and malondialdehyde level: no effect T4 level: DEHP ↓ Thyroid-stimulating hormone (TSH) level: DEHP ↑ T3, free triiodothyronine (FT3), and free thyroxine (FT4) level: no effect Potential biomarker metabolites of DEHP exposure: DEHP ↑ (L-allothreonine, creatinine, uric acid, retinyl ester, and L-palmitoylcarnitine)/↓ (glycocholic acid, LysoPC(18:3))	[247]
Rat	DEHP	1, 10, or 100 mg/kg bw/d	Oral gavage	LD1-21, lactational	PND22	Cardiac metabolism/function	Fetal bodyweight: DEHP ↓ (PND9-22) Fasting blood glucose level: DEHP ↑ (100) GLUT4 protein: DEHP ↓ (PND22) Phosphorylation GLUT4^ser488^: DEHP ↑ (100) Development of cardiac glucometabolic disorders: DEHP ↑ (♂)	Heart weight and deoxyglucose uptake: DEHP ↓ (PND22) Glucose oxidation in the cardiac muscle: DEHP ↓ (PND22: 10/PND22: 100) InsRβin in the cardiac muscle: DEHP ↓ (♂) IRS1 protein and phosphorylation of Akt^ser473^ in the cardiac muscle: DEHP ↓ (♂: 100) Phosphorylation of IRS1^tyr632^ in the cardiac muscle: DEHP ↓ (♂: 10/100)	[258]
Rat	DEHP	300 mg/kg bw/d	Oral gavage	12 weeks, postnatal (adult)	adult	Liver metabolism	Bodyweight: no effect Liver weight, viscera coefficients, and hepatic cord disorder expansion: DEHP ↑ ALAT, TC, and HDL-cholesterol levels: DEHP ↑ ASAT, TG, and LDL-cholesterol level: no effect Number of liver differentially expressed genes: DEHP ↑ (130)/↓ (117) Liver-modulated pathways: steroid catabolic process, regulation of intestinal cholesterol absorption, oxidation-reduction process, cholesterol metabolism, primary bile acid biosynthesis, and bile secretion	Liver malondialdehyde content: DEHP ↑ Liver total superoxide dismutase, total antioxidant capacity, and catalase activities: DEHP ↓ Liver GSH-px activities: no effect	[241]
Rat	DEHP	30, 300, or 750 mg/kg bw/d	Oral gavage	GD0-PND21	PND7, 14, and 21	Thyroid function	Serum total T3 level: no effect Serum total T4 and TSH level: DEHP ↓ (PND7/PND14/PND21: 300, 750) PND 14 and PND21 mRNA level of genes involved in thyroid development: DEHP ↑ (300: *NIS*, *TTF-1*/750: *NIS*, *PAX8*, and *TTF-1*) PND14 protein level of the genes involved in thyroid development: DEHP ↑ (300: NIS/750: NIS, PAX8, and TTF-1) PND21 protein level of the genes involved in thyroid development: DEHP ↑ (30: NIS/300: NIS, PAX8, TTF-1/750: NIS, PAX8, TTF-1) mRNA expression and protein level of thyroid peroxidase (TPO; involved in thyroid function): DEHP ↑ (750)	PND14 deiodinases (*Dio*) mRNA expression: DEHP ↑ (30: *Dio2*/300: *Dio1*, *Dio2*/750: *Dio1*, *Dio2*)/↓ (*Dio3*) PND21 deiodinases mRNA expression: DEHP ↑ (*Dio1*, *Dio2*)/↓ (*Dio3*) Ultrastructure damage of the thyroid: DEHP ↑	[246]
Rat	DEHP	1, 20, 50, 100, or 300 mg/kg bw/d	Oral gavage	GD14-PND0	GD19, PND3, and 21 and 60	Metabolism	PND21 number of adrenal glands differentially expressed genes: DEHP ↑ (100 (67), 300 (175)/↓ (100 (238), 300 (252)) PND60 number of adrenal glands differentially expressed genes: DEHP ↑ (100 (213), 300 (331))/↓ (100 (158), 300 (260)) PPAR signaling pathway: DEHP ↓ Steroid biosynthesis pathway: DEHP ↑ (PND60) PPAR-driven gene expression: DEHP ↓ (PND21-60: *Adipoq*, *Pck1*, *Ucp1*, *Fabp4*/PND21: *Lpl*, *Rxrg*, *Pparg*, *Ppargc1b*, *Srebf1*/PND60: *Hmgcr*, *Hmgcs1*)/↑ (PND60: *Ppard*) Differentially expressed gene involvement: lipid metabolism, adipocyte differentiation, adaptive thermogenesis, and gluconeogenesis Cholesterol biosynthesis pathway: DEHP ↑ (PND60: 100, 300)	Immune response pathways: DEHP ↑ MAPK signaling pathway: DEHP ↑ (PND21: 100/PND60: 100, 300) PND60 PPAR nuclear receptors mRNA levels: DEHP ↑ (100, 300 (*Ppard*))/↓ (*Ppara*) PND3 PPAR nuclear receptor mRNA level: DEHP ↓ (*Pparg*) Expression of genes involved in fatty acid, triacylglycerol, and ketone body metabolism: DEHP ↓ (PND21: 100, 300)	[259]
Rat	DEHP	0.25, 6.25 mg/kg bw/d	Oral gavage	GD0-PND21	PND0, 2, 15, and 21	Kidney development and fetal metabolism	Birth, PND15-21 bodyweight: DEHP ↓ (6.25) Weaning bodyweight: DEHP ↓ Absolute kidney weight: DEHP ↓ (♀ PND15: 6.25)/↑ (♂ PND21: 6.25) Kidney-to-bodyweight ratio: ↓ (♀ PND15: 0.25)/↑ (PND21: 6.25/♂ PND15: 6.25/♂ PND21: 6.25) Creatinine clearance rate: DEHP ↓ Serum creatine and blood urea nitrogen level: DEHP ↑ (♀/♂: 6.25) Renal PPARα protein expression: DEHP ↑ (PND0, PND21: 6.25) Renal PPARγ protein expression: DEHP ↑ (PND0, PND21) Serum renin level: DEHP ↑ (0.25)	Maternal postpartum weight, litter size, and sex ratio: no effect Proportion of cortex in the nephrogenic zone: DEHP ↑ Nephron number: DEHP ↓ Total glomerular volume: DEHP ↑ (♀ PND21: 0.25)/↓ (PND33) Glomerular alterations and tubular damage: DEHP ↑ Serum nitric oxide level: DEHP ↓	[260]
Rat	DBP	850 mg/kg bw/d	Oral perfusion	GD14-18	PND1, and 18 months of age	Renal development and fibrosis	Bodyweight, kidney size, and kidney-to-bodyweight ratio: DBP↓ (PND1) Renal *Foxd1*, *Wnt11*, *Pax2*, and *Gdnf* mRNA expression: DBP ↓ (PND1) Renal Bmp4, Cdh11, Ywhab, and Calm1 mRNA expression: DBP ↑ (PND1) Kidney alpha-smooth muscle actin (α-SMA), fibronectin, and TGF-β staining: DBP ↑ (18 months)	Swelling of the glomerular tufts: DBP ↑ (18 months) Tubular atrophy and tubular cell compression: DBP ↑ (18 months) Widening of intertubular spaces and thickening of the tubular basement membrane: DBP ↑ (18 months) Interstitial extracellular matrix accumulation: DBP ↑ (18 months)	[261]
Rat	DBP	850 mg/kg bw/d	Oral perfusion	GD12-18	PND70	Renal development and fibrosis	Bodyweight: DBP ↓ (♂) Kidney size and kidney-to-bodyweight ratio: DBP ↓ (♂) Renal fibrosis, kidney tubular damage, and atrophy: DBP ↑ (♂) Kidney interstitial extracellular matrix accumulation: DBP ↑ (♂)	Kidney Fgf10, Fgfr2, and AR protein levels: DBP ↓ (♂) Serum testosterone level: DBP ↓ (♂)	[262]
Rat	DBP	850 mg/kg bw/d	Oral gavage	GD14-18	PND1 and 8 weeks of age	Renal development and fibrosis	Fetal bodyweight and survival rate: DBP ↓ Kidney mRNA and protein expression level of RhoA and ROCK1: DBP ↑ (PND1)	Mother and fetal morbidity: no effect Maternal bodyweight gain: DBP ↓ (GD14-18) Gestational length: DBP ↑	[263]
Rat	DEHP, DBP	7, 70, or 700 mg/kg bw/d DEHP—5, 50, or 500 mg/kg bw/d	Oral gavage	GD13—PND21	PND4, 21, 22, 33, 78, 88, and 90	Metabolism	Bodyweight from PND16 to 21: DEHP ↑ (700)/DBP ↑ (5) Bodyweight gain from PND29 to 53: DEHP ↑ (♂: 700/♀: 70) Bodyweight: DBP ↑ (♂ PND43: 5) Bodyweight gain from PND57 to 67: DBP ↓ (♂: 50) PND60 bodyweight: DBP ↓ (♀: 50) Adulthood bodyweight, bodyweight gain and mesenteric, retroperitoneal, epididymal, and ovarian fat tissue weight: no effect Fasting plasma glucose concentration: DEHP ↑ (700)/DBP ↑ (500) Plasma cholesterol concentration: DEHP ↑ (♂: 700)/DBP ↑ (♂: 5) Plasma TG level: DBP ↑ (♀: 500) Glucose uptake in response to insulin overload: DEHP ↓ (700) Response to glucose overload: no effect Islets glucose-stimulated insulin secretion: DEHP ↓ (♂: 700)/↑ (♂: 70)	Dams bodyweight gain and organ weight: no effect Litter size and post-implantation loss: no effect AGI: DEHP ↓ (♂: 700) Age of preputial separation: DEHP ↑ (700) Age of puberty: DEHP ↑ (♂: 700/♀: 70, 700)/DBP ↑ (♀: 500) Number of pups with hypospadias and cryptorchidism: DEHP ↑ (700) Reproductive organs weight: no effect	[243]
Mouse	DEHP, DiNP	DEHP 25 mg/kg chow/d, DiNP 75 mg/kg chow/d or both	Oral route (food)	GD-15 to PND21	PND21 and 10 months	Liver metabolism	Number of DEGs at PND21: DiNP (♀ (61))/DEHP + DiNP (♀ (1)) Number of pathways enriched: DiNP (♀: 12, such as acetyl-CoA/♂: 15, such as alpha-amino acid and organic acid metabolic processes) Number of dysregulated PPAR target gene expressions involved in enriched pathways: DiNP (♀ (13)/♂ (15)) DNA methylation of *Cs* promoter: DEHP + DiNP ↓ (♀)/↑ (♂) *Cs* and *Acly* liver expression: DEHP + DiNP ↑ (♀) DNA methylation of *Acly* promoter: DEHP + DiNP ↓ (♀) DNA methylation of *Fasn* promoter and *Fasn* liver expression: DiNP ↑ (♀) Hepatic acylcarnitine levels: DEHP ↑/DiNP ↑/DEHP + DiNP ↑ Hepatic acetyl-CoA levels: DEHP + DiNP ↓ (♀: PND21)/↑ (♀: 10 months)		[237]
Rat	n-BuP	10, 100, or 500 mg/kg bw/d	Oral gavage	GD7-PND22	PND1, 6, 14, 16, 17, 22, and 80-90	Fetal development/Reproduction	Fetal AGD: n-BuP ↓ (10, 500) Ovary weights: n-BuP ↓ (PND17: 100/500) Ventral prostate, prostate, and seminal vesicle weight: n-BuP ↓ (PND90: 500) Epididymal weight: n-BuP ↑ (PND90: 100) Epididymal sperm count: n-BuP ↓ Testicular *Cyp19a1* expression: n-BuP ↓ (PND16) Expression level of germ cell, Sertoli cell, and Leydig cell markers: no effect (PND16) Ventral prostate epithelial area and ratio between epithelium and lumen: n-BuP ↓ (PND22: 100)	Maternal bodyweight and gestational length: no effect Litter size, survival rate, and fetal and postnatal bodyweights: no effect Offspring sexual maturation, and testis and epididymis histological examination: no effect Number of terminal mammary buds: BP ↑ (PND22: 100/500) Distance between breast tissue and lymph nodes: BP ↓ (PND22: 100)	[264]
Rat	n-BuP	850 mg/kg bw/d	Intragastric administration	GD14-18	PND1	Renal development	Kidney autophagy marker (LC3B, Beclin-1) staining and expression: n-BuP ↑ Kidney HhIP protein and mRNA expression involved in autophagy and hedgehog regulation: n-BuP ↑ mRNA expression of hedgehog signaling pathway-related gene (*Gli1*, *Ptch1*): BP ↓		[265]
Mouse	n-BuP	3.5 µg/week/mouse	Subcutaneous injection	GD0-PND28	PND28–PND84	Adipogenesis and food intake regulation	Bodyweight, fat mass, weekly food intake, fasting serum glucose, and leptin level: n-BuP ↑ (♀) Lean mass: n-BuP ↓ (♀) Glucose and insulin tolerances: no effect (PND63) Adipocyte size visceral adipose tissue: BP ↑ (♀) Serum adiponectin, resistin, ghrelin, and insulin level: no effect *A*dipose tissue expression of *glut4*, *insr*, and *pparg*: no effect	17β estradiol levels: no effect Hypothalamus *lepr* mRNA expression: n-BuP ↓ (♀) Hypothalamus *pomc* mRNA expression: n-BuP ↓ (♀: PND28, PND84) Hypothalamus *mc4r*, *agrp*, and *insr* mRNA expression: no effect Hypothalamus methylation of nPE1: n-BuP ↑ (♀ PND28, PND84)	[234]

Results in brackets correspond to the sex in which the effect was observable (♂, male; ♀, female), followed by stage at observation separated by a column from the doses administered at which the effect was observed, and, if so, followed by the number of DEGs in brackets. If one of the above-mentioned characteristics is not specified, the effect was observed for both sexes, at the different stages of observation and at the different doses used. Arrows indicate the direction of the effect: a downward arrow (↓) indicates a decrease and an upward arrow (↑) indicates an increase. ABCG5: ATP-binding cassette transporter G5; ACC1: acetyl-CoA carboxylase 1; Acly: ATP-citrate lyase; ACSL1: acyl-CoA synthetase long-chain family member 1; Adh1: alcohol dehydrogenase 1; Adipoq: adiponectin; Adrb3: adrenoceptor beta 3; AGPAT1: 1-acylglycerol-3-phosphate O-acyltransferase 1; Agrp: agouti-related protein; AGTR1A: angiotensin II receptor type 1A; AGTR1B: angiotensin II receptor type 1B; AGTR2: angiotensin II receptor type 2; Agtrap: angiotensin II receptor-associated protein; ALAT: alanine aminotransferase; ALB: albumin; ANP: atrial natriuretic peptide; Aox1: aldehyde oxidase 1; AR: androgen receptor; ASAT: aspartate aminotransferase; AT1R: angiotensin II receptor type 1; AT2R: angiotensin II receptor type 2; Atf4: activating transcription factor 4; Atf6: activating transcription factor 6; Bax: BCL2-associated X apoptosis regulator; Bcl-2: BCL2 apoptosis regulator; Bcrp: breast cancer resistance protein; Bip: heat shock protein family A (Hsp70) member 5 (or Hspa5); Bmp4: bone morphogenetic protein 4; n-BuP: butylparaben; BPA: bisphenol A; BPF: bisphenol F; BPS: bisphenol S; BUN: blood urea nitrogen; bw: bodyweight; Calm1: calmodulin 1; CCDC152: coiled-coil domain containing 152; CCL2: chemokine (C-C) ligand 2; Cdh11: cadherin 11; Cidea: cell death-inducing DFFA-like effector a; COL1A1: collagen-1a1; COLEC12: collectin subfamily member 12; CPT1α: carnitine palmitoyl transferase 1 alpha; CRTC2: CREB-regulated transcription coactivator 2; Cs: citrate synthase; Cyp1a2: cytochrome P450 family 1 subfamily A member 2; CYP7A1: cholesterol 7α-hydrolase; Cyp11b2: cytochrome P450 family 11 subfamily b: polypeptide 2; Cyp17: steroid 17-alpha-hydroxylase; Cyp19: cytochrome P450 family 19; Cyp19A1: cytochrome P450: family 19: subfamily a: polypeptide 1; d: day; D1: cyclin D1; DBP: dibutyl phthalate; DEHP: di(2-ethylhexyl) phthalate; DGAT1: diacylglycerol O-acyltransferase 1; DiNP: di-isononyl-phthalate; DNA: deoxyribonucleic acid; DNMT1: DNA methyltransferase 1; DNMT3A: DNA methyltransferase 3 alpha; DNMT3b: DNA methyltransferase 3 beta; ECE1: endothelin converting enzyme 1; EFAs: essential fatty acids; eNOS: endothelial nitric oxide synthase; Esr1: estrogen receptor 1; ESRRA: estrogen-related receptor alpha; F1: first generation: F2: second generation; F3: third generation; FA: fatty acid; Fabp4: fatty acid-binding protein 4; Fads: fatty acid desaturase 1; FAS: fatty acid synthase; Fgf10: fibroblast growth factor 10; Fgf21: fibroblast growth factor 21; Fgfr2: fibroblast growth factor 2; Foxd1: forkhead box D1; FoxO1: forkhead box O1; FSH: follicle-stimulating hormone; G-6-Pase: glucose-6-phosphatase; GD: gestational day; Gdnf: glial cell-derived neurotrophic factor; GJA5: gap junction protein alpha 5; Gli1: GLI family zinc finger 1; GLUT2: glucose transporter 2; GLUT4: glucose transporter 4; Gnpat: glyceronephosphate O-acyltransferase; GPAT: glycerol-3-phosphate acyltransferase; Gpd1: glycerol-3-phosphate dehydrogenase 1; GSEA: gene set enrichment analysis; GSH-px: glutathione peroxidase; GSK3β: glycogen synthase kinase 3 beta; GSR: glutathione reductase; HBB: hemoglobin subunit beta; HDLs: high-density lipoproteins; HhIP: hedgehog-interacting protein; Hnf1a: hepatocyte nuclear factor 1 homeobox A; HNF4-α: hepatocyte nuclear factor 4 alpha; IFN-γ: interferon gamma; IL-1β: interleukin 1 beta; IL-2: interleukin 2; IL-4: interleukin 4; IL-5: interleukin 5; IL-6: interleukin 6; IL-12p70: interleukin 12 heterodimer of p40 and p35 subunits; IL-13: interleukin 13; IL-17; interleukin 17; IL-21: interleukin 21; IL-23: interleukin 23; IsoBP: isobutylparaben; LC3B: microtubule-associated protein 1 light-chain 3 beta; LC-PUFAs: longer-chain polyunsaturated fatty acids; LDLs: low-density lipoproteins; LDLR: low-density lipoprotein receptor; lepr: leptin receptor; LH: luteinizing hormone; lncRNA: long noncoding ribonucleic acid; Lpl: lipoprotein lipase; LPS: lipopolysaccharide; LRP-1: LDLR-related protein 1; MafA: MAF bZIP transcription factor A; mc4r: melanocortin type 4 receptor; MDB2: methyl-CpG binding domain protein 2; MeCP2: methyl CpG binding protein 2; MGAT1: alpha-1,3-mannosyl-glycoprotein 2-beta-N-acetylglucosaminyltransferase 1; miRNA: microribonucleic acid; MMP7: matrix metallopeptidase 7; mRNA: messenger ribonucleic acid; Mrp3: multidrug resistance-associated protein 3; MSLN: mesothelin; mTOR: mammalian target of rapamycin; MUFAs: monounsaturated fatty acids; NIS: sodium iodide symporter; NP: 4-nonylphenol; nPE1: POMC neuronal enhancer; Npy: neuropeptide Y; OP: 4-tert octylphenol; Pax2: paired box 2; Pax4: paired box 4; Pax6: paired box 6; PAX8: paired box 8; Pck1: phosphoenolpyruvate carboxykinase 1; Pdx1: pancreatic and duodenal homeobox 1; PEPCK: phosphoenolpyruvate carboxykinase; PLS-DA: partial least-squares discriminant analysis; PND: postnatal day; PNPLA2: patatin-like phospholipase domain-containing 2; Pparg: peroxisome proliferator-activated receptor gamma; Ppargc1b: PPARγ coactivator 1; PRKCE: protein kinase C epsilon; Ptch1: patched 1; PUFAs: polyunsaturated fatty acids; RhoA: Ras homolog gene family member A; ROCK1: Rho-associated protein kinase 1; RORγt: retinoic acid-related orphan receptor t; Rxr-β: retinoid X receptor β; Rxrg: retinoid X receptor gamma; pomc: pro-opiomelanocortin; SCD-1: stearoyl-CoA desaturase 1; Slc2a2: solute carrier family 2 member 2; Slc19a2: solute carrier family 19 member 2; SLC44A4: solute carrier family 44 member 4; SMAD7: suppressor of mothers against decapentaplegic 7; SPRY1: sprouty RTK signaling antagonist 1; SR-BI: scavenger receptor class BI; Srebf1: sterol regulatory element-binding transcription factor 1; SREBP-1: sterol regulatory element-binding protein-1; snoRNA: small nucleolar ribonucleic acid; TC: total cholesterol; Tcf21: transcription factor 21; TCS: triclosan; TGs: triglycerides; TIF-1: thyroid transcription factor 1; TP: total protein; Ucp1: uncoupling protein 1; UDPGT: UDP glucuronosyltransferase; WFDC2: WAP four-disulfide core domain 2; Wnt11: Wnt family member 11; Ywhab: tyrosine 3-monooxygenase/tryptophan 5-monooxygenase activation protein beta.

### 6.2. Effects of PPPs on Cardiometabolic Disorders

#### 6.2.1. Epidemiological Data

Most studies examining environmental pollutants have reported associations with increased blood pressure in children. However, the literature reported only putative evidence for the effects of exposure to phenols or phthalates [239]. In human cohorts, the cardiovascular characteristics of the offspring after maternal exposure to PPPs have been poorly examined due to difficulties in assessing blood pressure in infants. Nevertheless, blood pressure was measured in 1277 children aged 6 to 11 years from the European Human Early-Life Exposome (HELIX) cohort. Increases in diastolic blood pressure in infants were observed with prenatal BPA exposure. Interestingly, blood pressure was also measured in 1015 Spanish preadolescents (mean age = 10.4 years), in whom prenatal exposure to BP-3 was associated with higher diastolic blood pressure [174].

Regarding parabens, a decrease in systolic blood pressure in preadolescents was observed with MeP, mainly in boys, and with n-BuP only in girls [174].

Regarding phthalates, prenatal exposure to these compounds was associated with lower systolic blood pressure at ages 4 and 7 in girls, but not boys [191], and with lower cholesterol levels at 8–14 years for both [266]. Vafeiadi et al. also observed a negative association between phthalate metabolite concentrations and systolic/diastolic blood pressure at 4 years in both sexes [198]. Among the few studies examining the association between prenatal phthalate exposure and children’s blood pressure, two studies reported a negative association between ∑DEHP and systolic/diastolic blood pressure [198] in the previously described European HELIX cohort, and between systolic blood pressure and MBzP [267]. Additionally, high- and low-molecular-weight phthalate mixtures were associated with a decreased metabolic score, which included waist circumference, systolic and diastolic blood pressure, triacylglycerol, high-density lipoprotein cholesterol (HDL), and insulin levels [202].

Most studies have highlighted the effects of phenols and phthalates on maternal health, child growth, and cardiometabolic outcomes, but the results are conflicting. Cardiometabolic risks in adulthood need to be confirmed and supported by further rigorous studies [268].

#### 6.2.2. Maternal Exposure to PPPs and Cardiovascular Outcomes in Experimental Models

A growing body of evidence suggests that early life exposure to BPA may also have a substantial influence on perinatal and postnatal cardiometabolic programming, contributing to higher cardiometabolic risks in adulthood [249].

In sheep, prenatal BPA exposure (0.5 mg/kg BW/day by injection for the last two-thirds of gestation) had no significant effect on blood pressure or cardiac morphometric measurements in the offspring. However, this exposure increased the atrial natriuretic peptide gene expression in the ventricles and reduced the collagen expression in the right ventricle. When the mothers are overfed, BPA amplifies septal hypertrophy and continues to block left ventricular hypertrophy and blood pressure. Prenatal BPA seems to thwart obesity-induced cardiovascular disorders [251]. The exposure of female Sprague–Dawley rats during the last two-thirds of gestation to a mixture of BPA (0.005 mg/kg bw/day) and a high dose of DEHP (7.5 mg/kg bw/day) produced postnatal outcomes, including increased relative heart weight in adult male offspring [158]. In mice, lifelong maternal exposure to DEHP (30 mg/kg bw through daily oral administration) until weaning had the following effects on 8-week-old offspring: (i) increased blood pressure, (ii) deregulated aortic eNOS (endothelial nitric oxide synthase) phosphorylation, and (iii) upregulated AT1R (angiotensin I receptor) protein expression (angiotensin II signaling) [236].

Additionally, this perinatal exposure to DEHP was shown to increase adiposity in the offspring with increased bodyweight and WAT- and BAT (white and brown adipose tissue)-to-body-weight ratios, along with impaired hepatic cholesterol metabolism (increased plasma and hepatic cholesterol). Linked to the cardiovascular effects of perinatal exposure to PPPs in offspring, a study in mice highlighted the potential role of DNA methylation in DEHP-induced cardiac effects and emphasized the importance of gender/sex as a biological variable in environmental health studies [253]. Exposure to DEHP (300 mg/kg bw/day) during the last third of gestation, reduced systolic and diastolic systemic arterial blood pressures, and locomotor activity, in PND200 male rats. This exposure to DEHP decreased aldosterone release in these males [259], while aldosterone increased in females. This suggests a sex-specific adrenal response to in utero exposure to DEHP and opens up the possibility of a hypertensive response induced by DEHP in the female offspring [256,257]. However, maternal exposure to a lower dose of DEHP (10 mg/kg bw/d) was shown to induce hypertension and bodyweight gain in male offspring [269]. Another study investigating the effects of maternal DEHP exposure during whole gestation showed impaired renal development in offspring. This led to a nephron deficit, and subsequently elevated blood pressure later in life, by the inhibition of the renin–angiotensin system [260]. Maternal exposure to DBP (850 mg/kg bw/day during GD14-18) induced kidney dysplasia and renal fibrosis in male offspring [261] by an expression of TGF-β1 and the abnormal activation of the epithelial–mesenchymal transition in fibrotic kidneys [270]. This exposure also decreased the testosterone concentration and reduced androgen receptor expression [262]. Maternal exposure to a lower dose of DEHP (10–100 mg/kg bw/d) induced a decreased heart weight and altered cardiac metabolic function in young offspring [258]. Maternal exposure to phthalic acid reduced the bodyweight (bw), heart weight (HW), and HW/bw in offspring, while their heart rates and blood pressures were conversely increased compared to the control group [271]. In the Sprague–Dawley rats, maternal exposure to DBP (850 mg/kg/day orally in the last third of gestation) induced renal fibrosis in the offspring [263,265].

These data highlight the difficulties in establishing a link between PPP exposure (alone or in combination or mixture) and the risk of metabolic and cardiovascular diseases. This is why it is necessary to develop new approaches using a new animal model to assess the long-term effects of exposure to PPPs, including at puberty and in adulthood, and taking into account the sex of the offspring.

### 6.3. Effects of PPPs on Gonadal Functions and Fertility in Cohorts and Animal Models

The gonads are the primary organs of the reproductive system responsible for the production of sex hormones and gametes. Thus, the gonads are particularly susceptible to endocrine disruptors, which are known to interfere with steroid hormone receptors. These include the estrogen receptor (ER), progesterone receptor (PR), and androgen receptor (AR), causing reproductive dysfunctions. Therefore, this part of the paper will be dedicated to the effect of maternal exposure to BPA, TCS, parabens, and phthalates or a mixture of pollutants on the gonadal functions of male and female offspring in cohorts and/or animal models.

#### 6.3.1. Epidemiological Data

Although difficult to undertake due to the duration of follow-up, the effects of prenatal exposure to PPPs on human reproduction are essential to understand the short-term and long-term effects on offspring [272].

One of the recognized markers of prenatal androgenization widely used and easily accessible is the measurement of anogenital distance [273]. In newborn boys (*n* = 72), but not in girls, a significant association between high cord blood BPA levels and shortened anoscrotal distance was suggested by Mammadov [274]. Similarly, boys whose mothers had detectable levels of BPA in their urine at 12–16 weeks of gestation were more likely to have shorter anogenital distance at birth, and at both at 6 and 12 months of age, but not in girls [275].

Related to puberty, the CHAMACOS cohort study reported an association of higher prenatal exposure to BPA with later puberty in girls and earlier puberty in boys [276]. In contrast, maternal urinary concentrations of BPA from three birth cohorts, the INMA (Spain), EDEN (France), and MoBa (Norway), were associated with delayed pubertal development in boys and girls [277]. In the study by Ferguson et al., prenatal exposure to BPA was associated with decreased odds of adrenarche (maturation of adrenal androgen secretion) and puberty in boys between 8 and 14 years of age [278].

To our knowledge, only two studies have investigated the association between the in utero exposure to phenol and reproduction in men in adulthood [279]. In the Western Australian Pregnancy Cohort, maternal BPA exposure at 18 and 34 weeks of gestation was positively associated with their sons’ sperm concentration and motility in adulthood (20–22 years), but not for their testicular function [279]. Furthermore, a high exposure to BPA and BP-3 during gestation was characterized by a compensated reduced Leydig cell function in adulthood, with no association with anogenital distance or semen quality [280].

Concerning prenatal TCS exposure, one study has explored its impact on reproductive hormones in cord blood, where TCS was associated with an increase in T and a decrease in E2 concentrations in cord blood among male infants [281]. Earlier puberty has been suggested in girls in all three birth cohorts, the INMA (Spain), EDEN (France), and MoBa (Norway) [277].

Later on, earlier menarche was reported with prenatal TCS exposure in girls from the CHAMACOS cohort, focusing on Latino children [282]. Using the same cohort, earlier breast development, pubic hair development, and menarche were observed with prenatal methylparaben (MeP) exposure in girls, and only earlier menarche with propylparaben. In boys, only genital development was reported associated with prenatal propylparaben exposure [282]. In addition, n-BuP was negatively associated with pubertal onset, adrenarche, and/or gonadarche (earlier gonadal development) in boys in the three European cohorts, the INMA (Spain), EDEN (France), and MoBa (Norway). In contrast, EtP and PrP were negatively associated with gonadarche in boys [277].

The association between prenatal phthalate exposure and gonadal development has been studied. Swan et al. reported an association between the anogenital distance, the incomplete testicular descent, the incomplete virilization, and the prenatal phthalate monoester metabolites exposure [283]. In the TIDES cohort from the USA, first-trimester urinary DEHP metabolite concentrations were associated with increased odds of genital abnormalities, especially due to hydrocele in newborn males [284]. In contrast, in a Canadian pregnancy cohort study, no strong evidence was observed between maternal urinary phthalates in the first trimester and the length or width of the penis at birth [285]. The association of prenatal exposure to phthalates with pubertal timing in boys and girls was investigated using several cohorts. Prenatal exposure to most phthalates was associated with reduced odds of adrenarche (notably with MEHHP, MEOHP, and MBzP) and reduced odds of puberty in association with DEHP metabolites, MBzP, MBP, and MiBP [278].

In the CHAMACOS longitudinal cohort study, it was found that the cx-MiNP, MCOP, and MCPP were associated with the late onset of pubarche (the appearance of pubic hair) and menarche (the first period) mostly among normal-weight girls, whereas MBzP was associated with later thelarche in girls. In boys, all of the phthalate biomarkers were associated with earlier gonadarche and pubarche, but associations with phthalate metabolites were close to the null or positive [276]. Moreover, prenatal urinary MEP concentrations were associated with an earlier onset of pubic hair development only in girls [282].

In the INMA Spanish cohort study, prenatal exposure to DEHP was associated with a higher risk of puberty onset and gonadarche in boys, and a higher risk of adrenarche in girls, at age 7–10 years. In boys, prenatal exposure to DEP, DnBP, and DEHP was also associated with a higher risk of adrenarche or gonadarche in children with normal weight, and BBzP and DiNCH exposure with lower risk of adrenarche in children with overweight/obesity. In girls, DiBP, DnBP, and DiNCH were associated with a higher risk of gonadarche in children with overweight/obesity [286]. A recent publication from Freire et al. reported that MEHP was associated with delayed gonadarche and adrenarche, respectively, in girls and boys [277].

Interestingly, associations between prenatal exposure to phthalates and peripubertal measures of male reproductive development seem to be dependent on the timing of in utero exposure. In fact, exposure to phthalates during the third trimester was associated with reduced odds of having a Tanner stage higher than one for pubic hair development and higher peripubertal SHBG (sex hormone-binding globulin) levels. Exposure to phthalates during the first- and second-trimester phthalates was not associated with these outcomes. In contrast, only during the first trimester, exposure to DEHP was associated with higher estradiol concentration [287]. The long-term effects on adult reproductive health in male offspring (18–20 years) were also investigated, demonstrating an association between high maternal exposure to some phthalates and an impaired Leydig cell function characterized by lower total and free testosterone/LH ratios [288].

All of these studies suggested that PPPs can affect male and female genital development, puberty, semen quality, and sexual hormones, but the effects can depend on the window of prenatal exposure.

#### 6.3.2. Maternal Exposure to PPPs and Gonadal Effects Using Animal Models

- Male Offspring

Most studies that have assessed the risk of perinatal exposure to BPA on male reproductive function have shown abnormal production of sex hormones in adulthood. In rats, daily exposure to 0.025 mg/kg of bodyweight or 0.250 mg/kg bw of BPA during the second half of gestation (GD10-21) reduced circulating testosterone levels (T) and luteinizing hormone (LH). This exposure also increased the levels of follicle-stimulating hormone (FSH) and estradiol (E2) [289]. Similar effects on blood levels of T and E2 are observed when exposure begins at the start of gestation (0.010 mg/kg bw) [290]. However, the opposite effects were observed for these hormones when measured at weaning [290]. Similarly, perinatal exposure (from GD18 to PND5) to 0.5 or 5 mg/kg bw of BPA results in a higher production of T [291]. In addition, exposure to BPA induces a decrease in AR expression and an increase in ER expression [291,292]. As a result, multiple abnormal histological and architectural damages are seen during testicular development. These include interstitial necrosis, germ cell degeneration, decreased tubular and luminal diameter [289,291,293], acrosome and plasma membrane integrity alteration, decreased mitochondrial activity [291], and increased oxidative stress [289]. Additionally, daily exposure to low levels of BPA (in the range of 0.0012 mg/kg bw and 5 mg/kg bw) significantly reduces sperm motility and count [289,291,292]. These outcomes lead to alterations in the fertility of the first male offspring and their subsequent second and third generations. However, this exposure does not affect the transcriptomic and epigenomic profile at the genome scale in sperm [292,293].

Studies conducted using mice or rabbits as a model have revealed similar morphological and hormonal damages in the testis of offspring exposed to BPA [248,294,295]. The observed effects on steroidogenesis are associated with a dysregulation of the steroidogenic enzyme expression. For example, daily exposure to BPA at 500 mg/kg bw from GD8 to PDN14 in mice decreased the expression of *StAR* (Steroidogenic Acute Regulatory Protein) and *Cyp11a* (Cytochrome P450 Family 11 Subfamily A Member 1) [296]. In rabbits, daily exposure to BPA at 50 mg/kg bw from GD15 to birth decreased the expression of *CYP11A1* and *3β-HSD* [297]. However, these effects are associated with different effects on T levels, which are reduced in adult mice and increased in 3-day-old rabbits. In addition, other transcriptomic studies showed that the expression of *Snrnp 40* (U5 small nuclear ribonucleoprotein subunit) was upregulated in the spliceosome pathway and that the expression of *Hnrnpu* (encoding a DNA- and RNA-binding protein) was downregulated. These expressions suggest that spliceosome blockage may be the cause of abnormal testicular development in male mice exposed to BPA [295]. On the other hand, prenatal BPA exposure upregulated the transcription level at PND21 of testicular *Dnmt1* and inhibited the transcription of testicular *Dnmt3A* and *Dnmt3B*. In addition, the transcriptional level of testicular caspase-7, caspase-9, and bax is increased, and the transcriptional level of bcl-2 is decreased at PND56, leading to apoptosis in the testis [295].

Gestational and postnatal exposure to phthalates also induced adverse effects in male offspring. In rabbits, daily exposure to DBP (400 mg/kg bw) in utero (GD15-29) reduced serum T levels and, consequently, the concentration and quality of ejaculated sperm and the weights of the testes and accessory sex glands. Interestingly, rabbits exposed to DBP developed cryptorchid testes with carcinoma in situ-like cells, malformed foreskin (giving the appearance of feminized external genitalia), hypospadias, hypoplasia and prostate, and agenesis of the bulbourethral gland [298]. In addition, gestational daily exposure (GD10-20) to DEHP equivalent to a human daily intake (0.0024–0.003 mg/kg of bw/day) in mice reduced the anogenital distance, seminal vesicle weight, expression of testicular steroidogenic enzymes (*Star*, *Cyp17a1*, *Hsd17b12*, *Hsd3b1*, and *Hsd3b6*), and sperm count. However, this exposure did not affect either the testicular morphology or fertility performances [299]. It was previously assumed that the effect of DEHP on T production and steroidogenic enzyme expression was strain-dependent in rats [300]. However, Hannas and al. reported very minor differences in the testicular and epidydimal phenotype between the Sprague–Dawley and Wistar rat strains [301].

Over the years, the use of animal models to understand the effects of maternal exposure to PPPs during pregnancy on the offspring phenotype has provided evidence that this exposure caused genital abnormalities in both male and female offspring. These studies were conducted by exposing animals to single compounds. However, on a daily basis, humans are exposed to dozens or even hundreds of chemical combinations through inhalation, skin contact, and ingestion. Consequently, the number of studies using mixtures of toxic substances is increasing. For example, an epidemiological study including 194 pregnant women (European EDC-MixRisk project (http://edcmixrisk.ki.se, accessed on 12 September 2024) reported a correlation between the levels of a mixture of phthalates (33% MBP, 16% MBzP, 21% MEHP, and 30% MiNP) in urine collected during the first trimester and the anogenital distance from their newborn boys. Repouskou et al. conducted an experimental study exposing mice throughout gestation to 0, 0.26, 2.6, and 13 mg/kg bw of this phthalate mixture [302]. As expected, they observed adverse effects on the male offspring reproductive system. These effects included a shorter anogenital distance, abnormal testicular development with thinner and disorganized seminiferous tubules, atypical germ cells, and low sperm production. At higher doses of this mixture, these effects were associated with increased circulating T and E2, and the increased expression of steroidogenic enzymes. Additionally, Hannas et al. tested a mixture of nine phthalates (DEHP, di-iso-heptyl phthalate, di-iso-butyl phthalate (DiBP), di-butyl phthalate (DBP), benzyl-butyl phthalate (BBP), di-cyclohexyl phthalate (DCHP), di-heptyl phthalate, di-hexyl phthalate (DnHP), and di-pentyl phthalate). They demonstrated that an administration of 650 mg/kg/day of this mixture from GD14 to GD18 reduced fetal T production in a dose-dependent manner [301]. Additionally, pregnant rats exposed to a combination of only two phthalates (DBP and DEHP; 500 mg/kg bw) within the same time window reduced fetal T production and decreased the expression of the steroidogenic enzymes *Star* and *Cyp11a* [303]. In this study, exposed male offspring were more likely to develop external and internal reproductive malformations, such as a shorter anogenital distance, hypospadias, external feminization (a higher number of nipples), testicular and epididymal malformations, and agenesis of the seminal vesicle and vas deferent. Interestingly, the mixture of different active molecules also interfered with normal sexual differentiation. Maternal and postnatal exposure to a combination of phthalates with genistein, polychlorinated biphenyls, or BPA also resulted in the low production of T, impaired seminiferous tubule development, and the reduction in testicular weight, sperm count, and sperm viability [304,305,306].

- Female Offspring

As for the male offspring, the harmful effects on female reproductive functions are induced by gestational or perinatal exposure to BPA, phthalates, or a mixture of pollutants. However, a limited number of studies focusing on female reproductive health have been published in the past five years.

In mice, gestational exposure to BPA (2.5, 5, 10, 20, and 40 mg/kg bw/day from GD0.5 to GD17.5) advanced puberty, induced atrophy of the ovary at adulthood, and female offspring were more likely to abort [307]. At higher doses, BPA (50, 500, and 2500 mg/kg bw) caused ovarian damage by increasing vacuole formation and decreasing the number of corpus granules [290,296]. In rats, prolonged exposure to BPA through drinking water (1 μg/mL or 10 μg/mL BPA from GD6 to PND21) caused similar damage to female sexual differentiation, such as advanced puberty and endometrial malformation [296,308]. Multiple hypotheses of the BPA action mechanism have been suggested. BPA has been shown to exert its effect by binding to the ER and mimicking the weak effect of estrogen. Interestingly, serum levels of E2, and ovarian ERa and ERb, were reduced in female offspring exposed in utero upon reaching adulthood. On the other hand, a decrease in the ovarian expression of Dnmt1, Dnmt3A, Dnmt3B, and Bcl2, and an increase in the relative expression of caspase-9, caspase-7, and bax, were seen in female offspring exposed to BPA [290,296,307]. Moreover, the activation of inflammation and abnormal autophagy via the TLR4/NF-κB and mTOR signaling pathways have been reported in the ovary and in the uterine tissue of exposed female offspring [308]. Altogether, these observations suggest that the exposure to BPA leads to the dysregulation of estrogen levels, altered DNA methylation, and the activation of inflammation and apoptosis in the ovaries of females of the next generation.

In females, the effects of maternal exposure to DEHP have negative effects on oocyte growth, meiotic maturation, and ovarian function [309]. Specifically, in first-generation ovarian primordial germ cells (GD12.5) and first- and second-generation oocytes (PND21), maternal exposure to DEHP (0.040 mg/kg bw/day, from GD0 to birth) decreases the methylation of CpG sites in the maternal imprinted gene *Igf2r* (insulin-like growth factor 2 receptor) and in the paternal imprinted gene *Peg3 (paternally expressed gene 3)* [310]. Additionally, Pocar et al. showed that a longer exposure to a higher level of DEHP (0.05 or 5 mg/kg bw/day, from GD0 to PND21) decreased the expression of *Cyp19a1* and Cyp17a1, and blocked the process of meiosis II in most oocytes after superovulation in adult female offspring [311]. More recently, Repouskou et al. reported a cumulative effect of a mixture of phthalates. Pregnant mice exposed to a mixture of four phthalate monoesters (33% MBP, 16% MBzP, 21% MEHP, and 30% MiNP at 0, 0.26, 2.6, and 13 mg/kg bw/day from GD0 to birth) reduced the number of preantral follicles (primary and secondary), increased follicular atresia, and reduced the Cyp19a1 expression in the ovaries of female offspring [302].

Altogether these studies conducted in animal models demonstrated that PPPs can affect gonadal development, puberty, steroidogenic enzymes, sexual hormones, semen, and oocyte quality.

## 7. Conclusions

Despite the implementation of regulations aimed at banning certain PPPs or reducing their concentrations in consumer products, the general population, including pregnant women, continues to be exposed daily to these pollutants. The objective of this review was to compile the current knowledge on the effects of prenatal exposure to these molecules on fetoplacental development and offspring health in the context of the developmental origins of health and disease concept (DOHaD).

Due to a short half-life, these molecules are rapidly eliminated in the urine in the form of one or more metabolites. Maternal urine sampling appears relevant for biomonitoring the exposure to these molecules. Worth noting, while spot urine samples are of interest in large populations for describing exposure, their use in etiologic studies is limited. Given the reported high intra-individual variability in urinary concentrations of some phenols and phthalates, these snapshot assessments imperfectly reflect the average exposure over a long period, such as pregnancy. This leads to measurement errors and estimated effects biased toward zero. As shown in a simulation study, for a compound with a high intra-individual variability, such as BPA (intraclass correlation coefficients of around 0.2), the bias in effect estimates can be as high as 80%. The collection of repeated urine samples in the period of interest is, therefore, necessary to properly assess the exposure to PPPs and their effects on human health [312]. Unfortunately, this approach is not always applied to cohorts due to logistical constraints. Additionally, preconception maternal exposure levels are rarely known, as expectant mothers are recruited into cohorts once their pregnancies have been established.

During gestation, PPPs from the maternal bloodstream are able to cross the placental barrier in a native form or as metabolites and reach the fetal circulation, thereby contributing to direct exposure to the fetus. In humans, depending on the compound, positive or negative associations have been observed with placental weight or newborn weight and size, with, in some cases, sex-specific effects. Some studies have also found associations with DNA methylation, but generally without exploring the link between these methylation patterns and gene expression or any biological significance.

In vitro and in vivo studies were used to investigate the individual effects of PPPs on placental function. In vitro, depending on their concentration, according to a non-monotonic dose–response relationship, i.e., a U-shaped curve, phenols may or may not affect the proliferation of placental trophoblast cells and their hormone production. On the other hand, with monotonic dose–response curves, i.e., progressive effects depending on their concentrations, phthalates show effects on the lipid content, hCG secretion, and cell fusion through PPARγ. The in vitro approach makes it possible to test several concentrations of PPPs, alone or in a mixture, and to decipher their mechanism of action, but it does not take into account the sub-chronic effects or the complexity of the different cell types that make up the placenta. It is, therefore, essential to complement these studies with in vivo models.

The use of different animal models made it possible to evaluate the effects of sub-chronic exposure, to test several concentrations, and to target different periods of gestation. BPA appears to affect the placental structure, the expression of hormone receptors, and the genes involved in DNA methylation, as well as the DNA methylation levels, while TCS affects the expression of nutrient transporters and hormones. Phthalate-related alterations have been reported in placental morphology, hormone production, vascularization, histopathology, and gene/protein expression [131]. It should be noted, however, that the effects of pollutants on placental function are never evaluated in a cocktail, and that the exposure time only partially covers the preconception and/or gestation periods. Furthermore, sex-specific effects are rarely taken into account in these studies.

In a DOHaD context, it is also important to take into account the effects of prenatal exposure on the postnatal phenotype to assess population risks and, if necessary, adapt the PPP regulations. In human cohorts, depending on the children’s sex, positive or negative associations have been established between prenatal exposure to PPPs and children’s BMI, blood pressure, gonadal function, or age at puberty. However, the phenotyping of offspring is sometimes limited in terms of exploratory physiological tests for obvious ethical reasons, and, given the intergenerational duration, postnatal monitoring is currently limited to puberty. It is, therefore, essential to establish the postnatal phenotype of the offspring reaching adulthood, and even transgenerationally, by generating animal models exposed to PPPs at different doses and exposure durations so to complement the data from human cohorts.

Depending on the level of in utero exposure, and on the age and sex of the offspring, bisphenols and phthalates have been shown to affect bodyweight, carbohydrate homeostasis, insulin sensitivity, and thyroid hormones in offspring. These pollutants also have cardiovascular effects. Bisphenols can lead to ventricular hypertrophy, while phthalates affect blood pressure, with the phenotypes depending on the sex. As PPPs are endocrine disruptors, these molecules disrupt gonad development, the age of puberty, sex hormone concentrations, and the quality of sperm and oocytes.

Future challenges will be to define a pollutant mix based on data from human cohorts, including fetoplacental biometric data and postnatal phenotyping data representative of the maternal exposome. The next challenge will be to study the effects of maternal exposure by the ingestion of this mixture of PPPs in an animal model mimicking human maternal exposure. In particular, this challenge needs to explore the effects on the development and fetoplacental growth, as well as on the postnatal phenotype, especially the cardiometabolic status and gonadal function, whilst taking into account the sex of the offspring. The question of the animal model is crucial. It is important to choose a model whose placenta is close to that of the human placenta.

Currently, rodent models are the most widely used. However, their placenta is made up of three layers of trophoblastic cells and is therefore more distant from the human placenta than that of lagomorphs [313]. This makes lagomorphs an interesting model to explore. Additionally, the establishment of placental epigenetic signatures seems to be a relevant issue to link to the postnatal phenotype, and thus to predict the health trajectory of the offspring. These data could then be extrapolated to follow-up cohorts to assess the potential phenotypic risks for children exposed in utero to PPPs. By doing so, preventive measures can be put in place to limit the exposure to PPPs, and regulations could be revised, if necessary.

## Data Availability

Not applicable.

## Abbreviations

AR: androgen receptor; AT1R: angiotensin 1 receptor; ATM: ataxia telangiectasia mutated; BAT: brown adipose tissue; BBP: benzyl butyl phthalate; Bcl-2: B cell lymphoma 2; 11beta-HSD2: 11-beta-hydroxysteroid dehydrogenase 2; BMI: body mass index; BMPP: bis (4-methyl-2-pentyl) phthalate; n-BuP: n-butylparaben; BP-1: benzophenone-1; BP-3: benzophenone-3; BP-8: benzophenone-8; BPA: bisphenol A; BPAF: bisphenol AF; BPAP: bisphenol AP; BPB: bisphenol B; BPC: bisphenol C; BPE: bisphenol E; BPF: bisphenol F; BPFL: bisphenol FL; BPM: bisphenol M; BPP: bisphenol P; BPS: bisphenol S; BPZ: bisphenol Z; bw: bodyweight; BzP: benzylparaben; CCL2: C-C motif chemokine ligand 2; CL: blood clearance; CRE: CBP-responsive element; CREB: cAMP-responsive element-binding protein; CRH: corticotrophin-releasing hormone; C_SS_: steady-state plasma concentration; CTR1: SLC31A1 for solute carrier family 31, member 1; cx-MiNP: mono(carboxy-iso-nonyl) phthalate; DBEP: bis (2-n-butoxyethyl) phthalate; DBP: di-n-butyl phthalate; DCHP: dicyclohexyl phthalate; 2,4-DCP: 2,4-dichlorophenol; 2,5-DCP: 5-dichlorophenol; DEEP: bis (2-ethoxyethyl) phthalate; DEHP: di-(2-ethylhexyl) phthalate; DEP: diethyl phthalate; DnHP: di-n-hexyl phthalate; DI: daily PPP intake; DiBP: diisobutyl phthalate; DiDP: di-isodecyl phthalate; DiNCH: di-iso-nonyl-cyclohexane-1,2-dicarboxylate; DiNP: di-isononyl phthalate; DMEP: bis(2-methoxyethyl) phthalate; DMP: di-methyl phthalate; DnBP: di-n-butyl phthalate; DNMT1: DNA (cytosine-5)-methyltransferase 1; DnOP: di-n-octyl phthalate; DNP: dinonyl phthalate; DPP: di-amyl phthalate; E2: estrogen; ECHA: European Chemicals Agency; EDCs: endocrine-disrupting chemicals; EFSA: European Food Safety Authority; EGF: epidermal growth factor; eNOS: endothelial nitric oxide synthase; EtP: ethylparaben; ER: estrogen receptor; ERE: estrogen response element; F: bioavailability; FPR: birthweight-to-placental-weight ratio; FSH: follicle-stimulating hormone; Fue: urinary excretion factors; GCNF: *Germ cell* nuclear factor; GLUT-1: glucose transporter 1; GWs: gestational weeks; hCG: human chorionic gonadotropin; HEPH: hephestin; HIF-1α: hypoxia-inducible factor 1-α; HMW: high-molecular-weight phthalates; HeP: heptylparaben; Hsp70: heat shock protein 70; 5-HT: serotonin; ICR: imprinting control region; IGF-1 or -2: insulin-like growth factor; IGF-2R: insulin-like growth factor-2 receptor; IGFBPs: IGF-binding proteins; IL: interleukin; i-BuPB: iso-butylparaben; i-PrPB: iso-propylparaben; LH: *luteinizing hormone*; LMW: low-molecular-weight phthalates; LXR: liver X receptor; MBP: mono-n-butyl phthalate; MBzP: monobenzyl phthalate; MCIOP: mono(4-methyl-7-carboxyheptyl) phthalate; MCMHP: mono-2-carboxy-methyl hexyl phthalate; MCOP: mono-carboxy-iso-octyl phthalate; MCPP: mono(3-carboxypropyl) phthalate; MECPP: mono(2-ethyl-5-carboxy-pentyl) phthalate; MECPTP: mono(2-ethyl-5-carboxy-pentyl) terephthalate; MEHHP: mono(2-ethyl-5-hydroxyhexyl) phthalate; MEHP: mono(2-ethyl-hexyl) phthalate; MEOHP: mono(2-ethyl-5-oxohexyl) phthalate; MEP: mono-ethyl phthalate; MiBP: mono-iso-butyl phthalate; MiDP: mono-iso-decyl phthalate; MiNP: mono-iso-nonyl phthalate; MMP-9 or 2: matrix metalloproteinase; MMP: monomethyl phthalate; MeP: methylparaben; mTOR: mammalian target of rapamycin; MW: molecular weight; NF-κB: nuclear factor κ-light-chain-enhancer of activated B cells; NIS: sodium iodide symporter; NP: 4-nonylphenol; NRF-2: erythroid 2-related factor 2; OP: 4-tert octylphenol; PAX8: paired box 8; PE: polyethylene; Peg3: paternally expressed gene 3; PET: polyethylene terephthalate; PFR: placental-to-birthweight ratio; PHBA: para-hydroxybenzoic acid; PMCA1: plasma membrane calcium ATPase; PND: postnatal day; PrP: propylparaben; PPAR: peroxisome proliferator-activated receptors; PPPs: combination of phenols, parabens, and phthalates; PR: progesterone receptor; PVA: polyvinyl acetate; PVC: polyvinyl chloride; Rb: retinoblastoma; REACH: registration, evaluation, authorization, and restriction of chemicals; RORγ: retinoid Z receptor; SF-1: steroidogenic factor-1; SNAT-1 or 4: sodium-coupled neutral amino acid transporters; SRD5A2: steroid 5α-reductase 2; SULT1E1: estrogen sulfotransferase 1E1; T: testosterone; T4: thyroxine; TBAARS: thiobarbituric acid-reactive substances; TBBPA: tetrabromobisphenolA; TCS: triclosan; TET1: Tet methylcytosine dioxygenase 1; TGF-β: transforming growth factor β; THB: 2,3,4-trihydroxybenzophenone; TLR4: Toll-like receptor 4; TIMP-3: tissue inhibitor of metalloproteinase 3; TKs: toxicokinetics; TPO: thyroid peroxidase; TSH: thyroid-stimulating hormone; TTF-1: thyroid transcription factor 1; UE: molar urinary excretion of the measured compound; UGT1A1: UDP-glucuronosyltransferase 1A1; UV_norm:_ daily excreted urinary volume; Vss: steady-state volume of distribution; WAT: white adipose tissue; WC: waist circumference.

## References

[1] Alharbi H.F., Algonaiman R., Alduwayghiri R., Aljutaily T., Algheshairy R.M., Almutairi A.S., Alharbi R.M., Alfurayh L.A., Alshahwan A.A., Alsadun A.F. (2022). Exposure to Bisphenol A Substitutes, Bisphenol S and Bisphenol F, and Its Association with Developing Obesity and Diabetes Mellitus: A Narrative Review. Int. J. Environ. Res. Public Health.

[2] Rolland M., Lyon-Caen S., Sakhi A.K., Pin I., Sabaredzovic A., Thomsen C., Slama R., Philippat C. (2020). Exposure to phenols during pregnancy and the first year of life in a new type of couple-child cohort relying on repeated urine biospecimens. Environ. Int..

[3] Ďurovcová I., Kyzek S., Fabová J., Makuková J., Gálová E., Ševčovičová A. (2022). Genotoxic potential of bisphenol A: A review. Environ. Pollut..

[4] Oliviero F., Marmugi A., Viguié C., Gayrard V., Picard-Hagen N., Mselli-Lakhal L. (2022). Are BPA Substitutes as Obesogenic as BPA?. Int. J. Mol. Sci..

[5] Dhillon G.S., Kaur S., Pulicharla R., Brar S.K., Cledón M., Verma M., Surampalli R.Y. (2015). Triclosan: Current status, occurrence, environmental risks and bioaccumulation potential. Int. J. Environ. Res. Public Health.

[6] Moss T., Howes D., Williams F.M. (2000). Percutaneous penetration and dermal metabolism of triclosan (2,4, 4′-trichloro-2′-hydroxydiphenyl ether). Food Chem. Toxicol..

[7] Weatherly L.M., Gosse J.A. (2017). Triclosan exposure, transformation, and human health effects. J. Toxicol. Environ. Health Part B Crit. Rev..

[8] Milanović M., Đurić L., Milošević N., Milić N. (2023). Comprehensive insight into triclosan-from widespread occurrence to health outcomes. Environ. Sci. Pollut. Res. Int..

[9] Wang C.F., Tian Y. (2015). Reproductive endocrine-disrupting effects of triclosan: Population exposure, present evidence and potential mechanisms. Environ. Pollut..

[10] Ruszkiewicz J.A., Li S., Rodriguez M.B., Aschner M. (2017). Is Triclosan a neurotoxic agent?. J. Toxicol. Environ. Health Part B Crit. Rev..

[11] Olaniyan L.W., Mkwetshana N., Okoh A.I. (2016). Triclosan in water, implications for human and environmental health. SpringerPlus.

[12] Witorsch R.J. (2014). Critical analysis of endocrine disruptive activity of triclosan and its relevance to human exposure through the use of personal care products. Crit. Rev. Toxicol..

[13] Juncker J.-C. (2016). Commission Impementing Decision Not Approving Triclosan as an Existing Active Substance for Use in Biocidal Products for Product-Type 1.

[14] Nowak K., Ratajczak-Wrona W., Górska M., Jabłońska E. (2018). Parabens and their effects on the endocrine system. Mol. Cell. Endocrinol..

[15] Hager E., Chen J., Zhao L. (2022). Minireview: Parabens Exposure and Breast Cancer. Int. J. Environ. Res. Public Health.

[16] Giuliani A., Zuccarini M., Cichelli A., Khan H., Reale M. (2020). Critical Review on the Presence of Phthalates in Food and Evidence of Their Biological Impact. Int. J. Environ. Res. Public Health.

[17] Wormuth M., Scheringer M., Vollenweider M., Hungerbühler K. (2006). What are the sources of exposure to eight frequently used phthalic acid esters in Europeans?. Risk Anal. Off. Publ. Soc. Risk Anal..

[18] Wang Y., Zhu H., Kannan K. (2019). A Review of Biomonitoring of Phthalate Exposures. Toxics.

[19] Philippat C., Rolland M., Lyon-Caen S., Pin I., Sakhi A.K., Sabaredzovic A., Thomsen C., Slama R. (2021). Pre- and early post-natal exposure to phthalates and DINCH in a new type of mother-child cohort relying on within-subject pools of repeated urine samples. Environ. Pollut..

[20] EFSA (2011). Report on the development of a Food Classification and Description System for exposure assessment and guidance on its implementation and use. EFSA J..

[21] EFSA (2006). Commission directive 2006/141/EC of 22 December 2006 on infant formulae and follow-on formulae and amending Directive 1999/21/EC. EFSA J..

[22] Genuis S.J., Beesoon S., Lobo R.A., Birkholz D. (2012). Human elimination of phthalate compounds: Blood, urine, and sweat (BUS) study. Sci. World J..

[23] Barker D.J., Gluckman P.D., Godfrey K.M., Harding J.E., Owens J.A., Robinson J.S. (1993). Fetal nutrition and cardiovascular disease in adult life. Lancet.

[24] Dekant W., Völkel W. (2008). Human exposure to bisphenol A by biomonitoring: Methods, results and assessment of environmental exposures. Toxicol. Appl. Pharmacol..

[25] Thayer K.A., Doerge D.R., Hunt D., Schurman S.H., Twaddle N.C., Churchwell M.I., Garantziotis S., Kissling G.E., Easterling M.R., Bucher J.R. (2015). Pharmacokinetics of bisphenol A in humans following a single oral administration. Environ. Int..

[26] Khmiri I., Côté J., Mantha M., Khemiri R., Lacroix M., Gely C., Toutain P.L., Picard-Hagen N., Gayrard V., Bouchard M. (2020). Toxicokinetics of bisphenol-S and its glucuronide in plasma and urine following oral and dermal exposure in volunteers for the interpretation of biomonitoring data. Environ. Int..

[27] Oh J., Choi J.W., Ahn Y.A., Kim S. (2018). Pharmacokinetics of bisphenol S in humans after single oral administration. Environ. Int..

[28] Sandborgh-Englund G., Adolfsson-Erici M., Odham G., Ekstrand J. (2006). Pharmacokinetics of triclosan following oral ingestion in humans. J. Toxicol. Environ. Health Part A.

[29] Kadry A.M., Okereke C.S., Abdel-Rahman M.S., Friedman M.A., Davis R.A. (1995). Pharmacokinetics of benzophenone-3 after oral exposure in male rats. J. Appl. Toxicol. JAT.

[30] Jeon H.K., Sarma S.N., Kim Y.J., Ryu J.C. (2008). Toxicokinetics and metabolisms of benzophenone-type UV filters in rats. Toxicology.

[31] Okereke C.S., Kadry A.M., Abdel-Rahman M.S., Davis R.A., Friedman M.A. (1993). Metabolism of benzophenone-3 in rats. Drug Metab. Dispos. Biol. Fate Chem..

[32] Abbas S., Greige-Gerges H., Karam N., Piet M.H., Netter P., Magdalou J. (2010). Metabolism of parabens (4-hydroxybenzoic acid esters) by hepatic esterases and UDP-glucuronosyltransferases in man. Drug Metab. Pharmacokinet..

[33] Wittassek M., Koch H.M., Angerer J., Brüning T. (2011). Assessing exposure to phthalates—The human biomonitoring approach. Mol. Nutr. Food Res..

[34] Frederiksen H., Jensen T.K., Jørgensen N., Kyhl H.B., Husby S., Skakkebæk N.E., Main K.M., Juul A., Andersson A.M. (2014). Human urinary excretion of non-persistent environmental chemicals: An overview of Danish data collected between 2006 and 2012. Reproduction.

[35] Philippat C., Calafat A.M. (2021). Comparison of strategies to efficiently combine repeated urine samples in biomarker-based studies. Environ. Res..

[36] Philippat C., Heude B., Botton J., Alfaidy N., Calafat A.M., Slama R. (2019). Prenatal Exposure to Select Phthalates and Phenols and Associations with Fetal and Placental Weight among Male Births in the EDEN Cohort (France). Environ. Health Perspect..

[37] Philippat C., Wolff M.S., Calafat A.M., Ye X., Bausell R., Meadows M., Stone J., Slama R., Engel S.M. (2013). Prenatal exposure to environmental phenols: Concentrations in amniotic fluid and variability in urinary concentrations during pregnancy. Environ. Health Perspect..

[38] Tefre de Renzy-Martin K., Frederiksen H., Christensen J.S., Boye Kyhl H., Andersson A.M., Husby S., Barington T., Main K.M., Jensen T.K. (2014). Current exposure of 200 pregnant Danish women to phthalates, parabens and phenols. Reproduction.

[39] Guilbert A., Rolland M., Pin I., Thomsen C., Sakhi A.K., Sabaredzovic A., Slama R., Guichardet K., Philippat C. (2021). Associations between a mixture of phenols and phthalates and child behaviour in a French mother-child cohort with repeated assessment of exposure. Environ. Int..

[40] Koch H.M., Bolt H.M., Angerer J. (2004). Di(2-ethylhexyl)phthalate (DEHP) metabolites in human urine and serum after a single oral dose of deuterium-labelled DEHP. Arch. Toxicol..

[41] Terry A.L., Cogswell M.E., Wang C.Y., Chen T.C., Loria C.M., Wright J.D., Zhang X., Lacher D.A., Merritt R.K., Bowman B.A. (2016). Feasibility of collecting 24-h urine to monitor sodium intake in the National Health and Nutrition Examination Survey. Am. J. Clin. Nutr..

[42] Moos R.K., Angerer J., Dierkes G., Brüning T., Koch H.M. (2016). Metabolism and elimination of methyl, iso- and n-butyl paraben in human urine after single oral dosage. Arch. Toxicol..

[43] Shin M.Y., Shin C., Choi J.W., Lee J., Lee S., Kim S. (2019). Pharmacokinetic profile of propyl paraben in humans after oral administration. Environ. Int..

[44] Koch H.M., Christensen K.L., Harth V., Lorber M., Brüning T. (2012). Di-n-butyl phthalate (DnBP) and diisobutyl phthalate (DiBP) metabolism in a human volunteer after single oral doses. Arch. Toxicol..

[45] Anderson W.A., Castle L., Scotter M.J., Massey R.C., Springall C. (2001). A biomarker approach to measuring human dietary exposure to certain phthalate diesters. Food Addit. Contam..

[46] Koch H.M., Angerer J. (2007). Di-iso-nonylphthalate (DINP) metabolites in human urine after a single oral dose of deuterium-labelled DINP. Int. J. Hyg. Environ. Health.

[47] Aylward L.L., Hays S.M., Gagné M., Krishnan K. (2009). Derivation of Biomonitoring Equivalents for di(2-ethylhexyl)phthalate (CAS No. 117-81-7). Regul. Toxicol. Pharmacol. RTP.

[48] Koch H.M., Calafat A.M. (2009). Human body burdens of chemicals used in plastic manufacture. Philos. Trans. R. Soc. Lond. Ser. B Biol. Sci..

[49] Gentry P.R., Clewell H.J., Clewell R., Campbell J., Van Landingham C., Shipp A.M. (2011). Challenges in the application of quantitative approaches in risk assessment: A case study with di-(2-ethylhexyl)phthalate. Crit. Rev. Toxicol..

[50] Rurak D.W., Wright M.R., Axelson J.E. (1991). Drug disposition and effects in the fetus. J. Dev. Physiol..

[51] Toutain P.L., Ferran A., Bousquet-Mélou A. (2010). Species differences in pharmacokinetics and pharmacodynamics. Handb. Exp. Pharmacol..

[52] Gayrard V., Moreau J., Picard-Hagen N., Helies V., Marchand P., Antignac J.P., Toutain P.L., Leandri R. (2021). Use of Mixture Dosing and Nonlinear Mixed Effect Modeling of Eight Environmental Contaminants in Rabbits to Improve Extrapolation Value of Toxicokinetic Data. Environ. Health Perspect..

[53] Schneider H., Albrecht C., Ahmed M.S., Broekhuizen M., Aengenheister L., Buerki-Thurnherr T., Danser A.H.J., Gil S., Hansson S.R., Greupink R. (2022). Ex vivo dual perfusion of an isolated human placenta cotyledon: Towards protocol standardization and improved inter-centre comparability. Placenta.

[54] Radke E.G., Braun J.M., Nachman R.M., Cooper G.S. (2020). Phthalate exposure and neurodevelopment: A systematic review and meta-analysis of human epidemiological evidence. Environ. Int..

[55] Corbel T., Gayrard V., Viguié C., Puel S., Lacroix M.Z., Toutain P.L., Picard-Hagen N. (2013). Bisphenol A disposition in the sheep maternal-placental-fetal unit: Mechanisms determining fetal internal exposure. Biol. Reprod..

[56] Morrison J.L., Berry M.J., Botting K.J., Darby J.R.T., Frasch M.G., Gatford K.L., Giussani D.A., Gray C.L., Harding R., Herrera E.A. (2018). Improving pregnancy outcomes in humans through studies in sheep. Am. J. Physiol. Regul. Integr. Comp. Physiol..

[57] Barry J.S., Anthony R.V. (2008). The pregnant sheep as a model for human pregnancy. Theriogenology.

[58] Gély C.A., Lacroix M.Z., Morin M., Vayssière C., Gayrard V., Picard-Hagen N. (2021). Comparison of the materno-fetal transfer of fifteen structurally related bisphenol analogues using an ex vivo human placental perfusion model. Chemosphere.

[59] Corbel T., Gayrard V., Puel S., Lacroix M.Z., Berrebi A., Gil S., Viguié C., Toutain P.L., Picard-Hagen N. (2014). Bidirectional placental transfer of Bisphenol A and its main metabolite, Bisphenol A-Glucuronide, in the isolated perfused human placenta. Reprod. Toxicol..

[60] Grandin F.C., Lacroix M.Z., Gayrard V., Viguié C., Mila H., de Place A., Vayssière C., Morin M., Corbett J., Gayrard C. (2019). Is bisphenol S a safer alternative to bisphenol A in terms of potential fetal exposure ? Placental transfer across the perfused human placenta. Chemosphere.

[61] Gély C.A., Picard-Hagen N., Chassan M., Garrigues J.C., Gayrard V., Lacroix M.Z. (2023). Contribution of Reliable Chromatographic Data in QSAR for Modelling Bisphenol Transport across the Human Placenta Barrier. Molecules.

[62] Gauderat G., Picard-Hagen N., Toutain P.L., Corbel T., Viguié C., Puel S., Lacroix M.Z., Mindeguia P., Bousquet-Melou A., Gayrard V. (2016). Bisphenol A glucuronide deconjugation is a determining factor of fetal exposure to bisphenol A. Environ. Int..

[63] Grandin F.C., Lacroix M.Z., Gayrard V., Gauderat G., Mila H., Toutain P.L., Picard-Hagen N. (2018). Bisphenol S instead of Bisphenol A: Toxicokinetic investigations in the ovine materno-feto-placental unit. Environ. Int..

[64] Gingrich J., Pu Y., Ehrhardt R., Karthikraj R., Kannan K., Veiga-Lopez A. (2019). Toxicokinetics of bisphenol A, bisphenol S, and bisphenol F in a pregnancy sheep model. Chemosphere.

[65] Andersen M.H.G., Zuri G., Knudsen L.E., Mathiesen L. (2021). Placental transport of parabens studied using an ex-vivo human perfusion model. Placenta.

[66] Fennell T.R., Krol W.L., Sumner S.C., Snyder R.W. (2004). Pharmacokinetics of dibutylphthalate in pregnant rats. Toxicol. Sci. Off. J. Soc. Toxicol..

[67] Saillenfait A.M., Payan J.P., Fabry J.P., Beydon D., Langonne I., Gallissot F., Sabate J.P. (1998). Assessment of the developmental toxicity, metabolism, and placental transfer of Di-n-butyl phthalate administered to pregnant rats. Toxicol. Sci. Off. J. Soc. Toxicol..

[68] Singh A.R., Lawrence W.H., Autian J. (1975). Maternal-fetal transfer of 14C-di-2-ethylhexyl phthalate and 14C-diethyl phthalate in rats. J. Pharm. Sci..

[69] Kremer J.J., Williams C.C., Parkinson H.D., Borghoff S.J. (2005). Pharmacokinetics of monobutylphthalate, the active metabolite of di-n-butylphthalate, in pregnant rats. Toxicol. Lett..

[70] Calafat A.M., Brock J.W., Silva M.J., Gray L.E., Reidy J.A., Barr D.B., Needham L.L. (2006). Urinary and amniotic fluid levels of phthalate monoesters in rats after the oral administration of di(2-ethylhexyl) phthalate and di-n-butyl phthalate. Toxicology.

[71] Mose T., Knudsen L.E., Hedegaard M., Mortensen G.K. (2007). Transplacental transfer of monomethyl phthalate and mono(2-ethylhexyl) phthalate in a human placenta perfusion system. Int. J. Toxicol..

[72] Mose T., Mortensen G.K., Hedegaard M., Knudsen L.E. (2007). Phthalate monoesters in perfusate from a dual placenta perfusion system, the placenta tissue and umbilical cord blood. Reprod. Toxicol..

[73] Ihde E.S., Zamudio S., Loh J.M., Zhu Y., Woytanowski J., Rosen L., Liu M., Buckley B. (2018). Application of a novel mass spectrometric (MS) method to examine exposure to Bisphenol-A and common substitutes in a maternal fetal cohort. Hum. Ecol. Risk Assess. HERA.

[74] Liu J., Li J., Wu Y., Zhao Y., Luo F., Li S., Yang L., Moez E.K., Dinu I., Martin J.W. (2017). Bisphenol A Metabolites and Bisphenol S in Paired Maternal and Cord Serum. Environ. Sci. Technol..

[75] Casas M., Valvi D., Ballesteros-Gomez A., Gascon M., Fernández M.F., Garcia-Esteban R., Iñiguez C., Martínez D., Murcia M., Monfort N. (2016). Exposure to Bisphenol A and Phthalates during Pregnancy and Ultrasound Measures of Fetal Growth in the INMA-Sabadell Cohort. Environ. Health Perspect..

[76] Ferguson K.K., Meeker J.D., Cantonwine D.E., Mukherjee B., Pace G.G., Weller D., McElrath T.F. (2018). Environmental phenol associations with ultrasound and delivery measures of fetal growth. Environ. Int..

[77] Vrijens K., Van Overmeire I., De Cremer K., Neven K.Y., Carollo R.M., Vleminckx C., Van Loco J., Nawrot T.S. (2020). Weight and head circumference at birth in function of placental paraben load in Belgium: An ENVIRONAGE birth cohort study. Environ. Health.

[78] Zhu Y.D., Gao H., Huang K., Zhang Y.W., Cai X.X., Yao H.Y., Mao L.J., Ge X., Zhou S.S., Xu Y.Y. (2018). Prenatal phthalate exposure and placental size and shape at birth: A birth cohort study. Environ. Res..

[79] Gao H., Geng M.L., Huang K., Zhu B.B., Zhang C., Gan H., Tong J., Wu X.L., Hu C.Y., Zhang S.Y. (2022). Relationship of individual and mixed prenatal phthalate exposure with placental structure and efficiency in the prospective Ma’anshan Birth Cohort Study. Sci. Total Environ..

[80] Mustieles V., Mínguez-Alarcón L., Christou G., Ford J.B., Dimitriadis I., Hauser R., Souter I., Messerlian C. (2019). Placental weight in relation to maternal and paternal preconception and prenatal urinary phthalate metabolite concentrations among subfertile couples. Environ. Res..

[81] Huang Y., Li J., Garcia J.M., Lin H., Wang Y., Yan P., Wang L., Tan Y., Luo J., Qiu Z. (2014). Phthalate levels in cord blood are associated with preterm delivery and fetal growth parameters in Chinese women. PLoS ONE.

[82] Jovanovic N., Mustieles V., Althuser M., Lyon-Caen S., Alfaidy N., Thomsen C., Sakhi A.K., Sabaredzovic A., Bayat S., Couturier-Tarrade A. (2024). Associations between synthetic phenols, phthalates, and placental growth/function: A longitudinal cohort with exposure assessment in early pregnancy. Hum. Reprod. Open.

[83] Ouidir M., Jedynak P., Rolland M., Lyon-Caen S., Thomsen C., Sakhi A.K., Sabaredzovic A., Bayat S., Slama R., Philippat C. (2024). Analyzing the impact of phthalate and DINCH exposure on fetal growth in a cohort with repeated urine collection. Environ. Int..

[84] Zhu Y., Wan Y., Zhang B., Zhou A., Huo W., Wu C., Liu H., Jiang Y., Chen Z., Jiang M. (2018). Relationship between maternal phthalate exposure and offspring size at birth. Sci. Total Environ..

[85] LaRocca J., Binder A.M., McElrath T.F., Michels K.B. (2014). The impact of first trimester phthalate and phenol exposure on IGF2/H19 genomic imprinting and birth outcomes. Environ. Res..

[86] Zhao Y., Chen J., Wang X., Song Q., Xu H.H., Zhang Y.H. (2016). Third trimester phthalate exposure is associated with DNA methylation of growth-related genes in human placenta. Sci. Rep..

[87] Zhao Y., Shi H.J., Xie C.M., Chen J., Laue H., Zhang Y.H. (2015). Prenatal phthalate exposure, infant growth, and global DNA methylation of human placenta. Environ. Mol. Mutagen..

[88] Lövkvist C., Dodd I.B., Sneppen K., Haerter J.O. (2016). DNA methylation in human epigenomes depends on local topology of CpG sites. Nucleic Acids Res..

[89] Grindler N.M., Vanderlinden L., Karthikraj R., Kannan K., Teal S., Polotsky A.J., Powell T.L., Yang I.V., Jansson T. (2018). Exposure to Phthalate, an Endocrine Disrupting Chemical, Alters the First Trimester Placental Methylome and Transcriptome in Women. Sci. Rep..

[90] Jedynak P., Maitre L., Guxens M., Gützkow K.B., Julvez J., López-Vicente M., Sunyer J., Casas M., Chatzi L., Gražulevičienė R. (2021). Prenatal exposure to a wide range of environmental chemicals and child behaviour between 3 and 7 years of age—An exposome-based approach in 5 European cohorts. Sci. Total Environ..

[91] Jedynak P., Tost J., Calafat A.M., Bourova-Flin E., Broséus L., Busato F., Forhan A., Heude B., Jakobi M., Schwartz J. (2022). Pregnancy exposure to phthalates and DNA methylation in male placenta—An epigenome-wide association study. Environ. Int..

[92] Svendsen A.J., Gervin K., Lyle R., Christiansen L., Kyvik K., Junker P., Nielsen C., Houen G., Tan Q. (2016). Differentially Methylated DNA Regions in Monozygotic Twin Pairs Discordant for Rheumatoid Arthritis: An Epigenome-Wide Study. Front. Immunol..

[93] Jedynak P., Siroux V., Broséus L., Tost J., Busato F., Gabet S., Thomsen C., Sakhi A.K., Sabaredzovic A., Lyon-Caen S. (2024). Epigenetic footprints: Investigating placental DNA methylation in the context of prenatal exposure to phenols and phthalates. Environ. Int..

[94] Puvvula J., Braun J.M., DeFranco E.A., Ho S.M., Leung Y.K., Huang S., Zhang X., Vuong A.M., Kim S.S., Percy Z. (2024). Gestational exposure to environmental chemicals and epigenetic alterations in the placenta and cord blood mononuclear cells. Epigenetics. Commun..

[95] Ticiani E., Gingrich J., Pu Y., Vettathu M., Davis J., Martin D., Petroff M.G., Veiga-Lopez A. (2021). Bisphenol S and Epidermal Growth Factor Receptor Signaling in Human Placental Cytotrophoblasts. Environ. Health Perspect..

[96] Lapehn S., Houghtaling S., Ahuna K., Kadam L., MacDonald J.W., Bammler T.K., LeWinn K.Z., Myatt L., Sathyanarayana S., Paquette A.G. (2023). Mono(2-ethylhexyl) phthalate induces transcriptomic changes in placental cells based on concentration, fetal sex, and trophoblast cell type. Arch. Toxicol..

[97] Shoaito H., Petit J., Chissey A., Auzeil N., Guibourdenche J., Gil S., Laprévote O., Fournier T., Degrelle S.A. (2019). The Role of Peroxisome Proliferator–Activated Receptor Gamma (PPARγ) in Mono(2-ethylhexyl) Phthalate (MEHP)-Mediated Cytotrophoblast Differentiation. Environ. Health Perspect..

[98] Adibi J.J., Zhao Y., Zhan L.V., Kapidzic M., Larocque N., Koistinen H., Huhtaniemi I.T., Stenman U.H. (2017). An Investigation of the Single and Combined Phthalate Metabolite Effects on Human Chorionic Gonadotropin Expression in Placental Cells. Environ. Health Perspect..

[99] Kliman H.J., Nestler J.E., Sermasi E., Sanger J.M., Strauss J.F. (1986). 3rd. Purification, characterization, and in vitro differentiation of cytotrophoblasts from human term placentae. Endocrinology.

[100] Zou Z., Harris L.K., Forbes K., Heazell A.E.P. (2022). Sex-specific effects of bisphenol A on the signaling pathway of ESRRG in the human placenta. Biol. Reprod..

[101] Alsubaie A.M., Arita Y., Atwater M., Mahfuz A., Peltier M.R. (2021). Enhancement of placental inflammation by Dibutyl Phthalate. J. Reprod. Immunol..

[102] Sullivan M.H. (2004). Endocrine cell lines from the placenta. Mol. Cell Endocrinol..

[103] Olivier E., Wakx A., Fouyet S., Dutot M., Rat P. (2021). JEG-3 placental cells in toxicology studies: A promising tool to reveal pregnancy disorders. Anat. Cell Biol..

[104] Graham C.H., Hawley T.S., Hawley R.G., MacDougall J.R., Kerbel R.S., Khoo N., Lala P.K. (1993). Establishment and characterization of first trimester human trophoblast cells with extended lifespan. Exp. Cell Res..

[105] Gu X., Liu H., Luo W., Wang X., Wang H., Li L. (2022). Di-2-ethylhexyl phthalate-induced miR-155-5p promoted lipid metabolism via inhibiting cAMP/PKA signaling pathway in human trophoblastic HTR-8/Svneo cells. Reprod. Toxicol..

[106] Wice B., Menton D., Geuze H., Schwartz A.L. (1990). Modulators of cyclic AMP metabolism induce syncytiotrophoblast formation in vitro. Exp. Cell Res..

[107] Orendi K., Gauster M., Moser G., Meiri H., Huppertz B. (2010). The choriocarcinoma cell line BeWo: Syncytial fusion and expression of syncytium-specific proteins. Reproduction.

[108] Gerbaud P., Pidoux G. (2015). Review: An overview of molecular events occurring in human trophoblast fusion. Placenta.

[109] Pastuschek J., Nonn O., Gutiérrez-Samudio R.N., Murrieta-Coxca J.M., Müller J., Sanft J., Huppertz B., Markert U.R., Groten T., Morales-Prieto D.M. (2021). Molecular characteristics of established trophoblast-derived cell lines. Placenta.

[110] Drwal E., Rak A., Gregoraszczuk E. (2018). Co-culture of JEG-3, BeWo and syncBeWo cell lines with adrenal H295R cell line: An alternative model for examining endocrine and metabolic properties of the fetoplacental unit. Cytotechnology.

[111] Msheik H., El Hayek S., Bari M.F., Azar J., Abou-Kheir W., Kobeissy F., Vatish M., Daoud G. (2019). Transcriptomic profiling of trophoblast fusion using BeWo and JEG-3 cell lines. Mol. Hum. Reprod..

[112] Ma R., Tang N., Feng L., Wang X., Zhang J., Ren X., Du Y., Ouyang F. (2021). Effects of triclosan exposure on placental extravillous trophoblast motility, relevant IGF2/H19 signaling and DNA methylation-related enzymes of HTR-8/SVneo cell line. Ecotoxicol. Environ. Saf..

[113] Agay-Shay K., Martinez D., Valvi D., Garcia-Esteban R., Basagaña X., Robinson O., Casas M., Sunyer J., Vrijheid M. (2015). Exposure to Endocrine-Disrupting Chemicals during Pregnancy and Weight at 7 Years of Age: A Multi-pollutant Approach. Environ. Health Perspect..

[114] Msheik H., Azar J., El Sabeh M., Abou-Kheir W., Daoud G. (2020). HTR-8/SVneo: A model for epithelial to mesenchymal transition in the human placenta. Placenta.

[115] Mannelli C., Ietta F., Carotenuto C., Romagnoli R., Szostek A.Z., Wasniewski T., Skarzynski D.J., Paulesu L. (2014). Bisphenol A alters β-hCG and MIF release by human placenta: An in vitro study to understand the role of endometrial cells. Mediat. Inflamm..

[116] Mørck T.J., Sorda G., Bechi N., Rasmussen B.S., Nielsen J.B., Ietta F., Rytting E., Mathiesen L., Paulesu L., Knudsen L.E. (2010). Placental transport and in vitro effects of Bisphenol A. Reprod. Toxicol..

[117] Rajakumar C., Guan H., Langlois D., Cernea M., Yang K. (2015). Bisphenol A disrupts gene expression in human placental trophoblast cells. Reprod. Toxicol..

[118] Benachour N., Aris A. (2009). Toxic effects of low doses of Bisphenol-A on human placental cells. Toxicol. Appl. Pharmacol..

[119] Wang X.K., Agarwal M., Parobchak N., Rosen A., Vetrano A.M., Srinivasan A., Wang B., Rosen T. (2016). Mono-(2-Ethylhexyl) Phthalate Promotes Pro-Labor Gene Expression in the Human Placenta. PLoS ONE.

[120] de Aguiar Greca S.C., Kyrou I., Pink R., Randeva H., Grammatopoulos D., Silva E., Karteris E. (2020). Involvement of the Endocrine-Disrupting Chemical Bisphenol A (BPA) in Human Placentation. J. Clin. Med..

[121] Ponniah M., Billett E.E., De Girolamo L.A. (2015). Bisphenol A increases BeWo trophoblast survival in stress-induced paradigms through regulation of oxidative stress and apoptosis. Chem. Res. Toxicol..

[122] Cao Y., Chen S., Lu J., Zhang M., Shi L., Qin J., Lv J., Li D., Ma L., Zhang Y. (2023). BPA induces placental trophoblast proliferation inhibition and fetal growth restriction by inhibiting the expression of SRB1. Environ. Sci. Pollut. Res. Int..

[123] Narciso L., Ietta F., Romagnoli R., Paulesu L., Mantovani A., Tait S. (2019). Effects of Bisphenol A on endogenous retroviral envelopes expression and trophoblast fusion in BeWo cells. Reprod. Toxicol..

[124] Wang Z.Y., Lu J., Zhang Y.Z., Zhang M., Liu T., Qu X.L. (2015). Effect of Bisphenol A on invasion ability of human trophoblastic cell line BeWo. Int. J. Clin. Exp. Pathol..

[125] Lu Y., Yang R., Yin N., Faiola F. (2021). In vivo and in vitro transcriptomics meta-analyses reveal that BPA may affect TGF-beta signaling regardless of the toxicology system employed. Environ. Pollut..

[126] Kim M.J., Kim C.H., An M.J., Lee J.H., Shin G.S., Song M., Kim J.W. (2020). Ethylparaben induces apoptotic cell death in human placenta BeWo cells via the Caspase-3 pathway. Anim. Cells Syst..

[127] Petit J., Wakx A., Gil S., Fournier T., Auzeil N., Rat P., Laprévote O. (2018). Lipidome-wide disturbances of human placental JEG-3 cells by the presence of MEHP. Biochimie.

[128] Yang C., Lim W., Bazer F.W., Song G. (2018). Butyl paraben promotes apoptosis in human trophoblast cells through increased oxidative stress-induced endoplasmic reticulum stress. Environ. Toxicol..

[129] Wang C., Niu Y., Xu L., Song L., Yin L., Zheng X., Chu J., Ma T. (2023). Effects of phthalates on human chorionic trophoblast cells and mouse embryonic development. Reprod. Toxicol..

[130] Du Z.P., Feng S., Li Y.L., Li R., Lv J., Ren W.Q., Feng Q.W., Liu P., Wang Q.N. (2020). Di-(2-ethylhexyl) phthalate inhibits expression and internalization of transthyretin in human placental trophoblastic cells. Toxicol. Appl. Pharmacol..

[131] Seymore T.N., Rivera-Núñez Z., Stapleton P.A., Adibi J.J., Barrett E.S. (2022). Phthalate Exposures and Placental Health in Animal Models and Humans: A Systematic Review. Toxicol. Sci. Off. J. Soc. Toxicol..

[132] Bhurke A., Davila J., Flaws J.A., Bagchi M.K., Bagchi I.C. (2023). Exposure to di-isononyl phthalate during early pregnancy disrupts decidual angiogenesis and placental development in mice. Reprod. Toxicol..

[133] Barberio L., Paulesu L., Canesi L., Grasselli E., Mandalà M. (2021). Bisphenol a Interferes with Uterine Artery Features and Impairs Rat Feto-Placental Growth. Int. J. Mol. Sci..

[134] Song W., Puttabyatappa M., Zeng L., Vazquez D., Pennathur S., Padmanabhan V. (2020). Developmental programming: Prenatal bisphenol A treatment disrupts mediators of placental function in sheep. Chemosphere.

[135] Zhang H., Zheng Y., Liu X., Zha X., Elsabagh M., Ma Y., Jiang H., Wang H., Wang M. (2023). Autophagy attenuates placental apoptosis, oxidative stress and fetal growth restriction in pregnant ewes. Environ. Int..

[136] Müller J.E., Meyer N., Santamaria C.G., Schumacher A., Luque E.H., Zenclussen M.L., Rodriguez H.A., Zenclussen A.C. (2018). Bisphenol A exposure during early pregnancy impairs uterine spiral artery remodeling and provokes intrauterine growth restriction in mice. Sci. Rep..

[137] Tachibana T., Wakimoto Y., Nakamuta N., Phichitraslip T., Wakitani S., Kusakabe K., Hondo E., Kiso Y. (2007). Effects of bisphenol A (BPA) on placentation and survival of the neonates in mice. J. Reprod. Dev..

[138] Tait S., Tassinari R., Maranghi F., Mantovani A. (2015). Bisphenol A affects placental layers morphology and angiogenesis during early pregnancy phase in mice. J. Appl. Toxicol. JAT.

[139] Lan X., Fu L.J., Zhang J., Liu X.Q., Zhang H.J., Zhang X., Ma M.F., Chen X.M., He J.L., Li L.B. (2017). Bisphenol A exposure promotes HTR-8/SVneo cell migration and impairs mouse placentation involving upregulation of integrin-β1 and MMP-9 and stimulation of MAPK and PI3K signaling pathways. Oncotarget.

[140] Ye Y., Tang Y., Xiong Y., Feng L., Li X. (2019). Bisphenol A exposure alters placentation and causes preeclampsia-like features in pregnant mice involved in reprogramming of DNA methylation of WNT2. FASEB J..

[141] Imanishi S., Manabe N., Nishizawa H., Morita M., Sugimoto M., Iwahori M., Miyamoto H. (2003). Effects of oral exposure of bisphenol A on mRNA expression of nuclear receptors in murine placentae assessed by DNA microarray. J. Reprod. Dev..

[142] Kang E.R., Iqbal K., Tran D.A., Rivas G.E., Singh P., Pfeifer G.P., Szabó P.E. (2011). Effects of endocrine disruptors on imprinted gene expression in the mouse embryo. Epigenetics.

[143] Susiarjo M., Sasson I., Mesaros C., Bartolomei M.S. (2013). Bisphenol a exposure disrupts genomic imprinting in the mouse. PLoS Genet..

[144] Mao J., Kinkade J.A., Bivens N.J., Rosenfeld C.S. (2021). miRNA changes in the mouse placenta due to bisphenol A exposure. Epigenomics.

[145] Lee J.H., Ahn C., Kang H.Y., Hong E.J., Hyun S.H., Choi K.C., Jeung E.B. (2016). Effects of Octylphenol and Bisphenol A on the Metal Cation Transporter Channels of Mouse Placentas. Int. J. Environ. Res. Public Health.

[146] Hong E.J., Choi K.C., Jeung E.B. (2003). Maternal-fetal transfer of endocrine disruptors in the induction of Calbindin-D9k mRNA and protein during pregnancy in rat model. Mol. Cell. Endocrinol..

[147] Hong E.J., Choi K.C., Jeung E.B. (2005). Maternal exposure to bisphenol a during late pregnancy resulted in an increase of Calbindin-D9k mRNA and protein in maternal and postnatal rat uteri. J. Reprod. Dev..

[148] Benincasa L., Mandalà M., Paulesu L., Barberio L., Ietta F. (2020). Prenatal Nutrition Containing Bisphenol A Affects Placenta Glucose Transfer: Evidence in Rats and Human Trophoblast. Nutrients.

[149] Tan W., Huang H., Wang Y., Wong T.Y., Wang C.C., Leung L.K. (2013). Bisphenol A differentially activates protein kinase C isoforms in murine placental tissue. Toxicol. Appl. Pharmacol..

[150] Gingrich J., Pu Y., Roberts J., Karthikraj R., Kannan K., Ehrhardt R., Veiga-Lopez A. (2018). Gestational bisphenol S impairs placental endocrine function and the fusogenic trophoblast signaling pathway. Arch. Toxicol..

[151] Mao J., Jain A., Denslow N.D., Nouri M.Z., Chen S., Wang T., Zhu N., Koh J., Sarma S.J., Sumner B.W. (2020). Bisphenol A and bisphenol S disruptions of the mouse placenta and potential effects on the placenta-brain axis. Proc. Natl. Acad. Sci. USA.

[152] Crawford B.R., Decatanzaro D. (2012). Disruption of blastocyst implantation by triclosan in mice: Impacts of repeated and acute doses and combination with bisphenol-A. Reprod. Toxicol..

[153] Jackson E.N., Rowland-Faux L., James M.O., Wood C.E. (2018). Administration of low dose triclosan to pregnant ewes results in placental uptake and reduced estradiol sulfotransferase activity in fetal liver and placenta. Toxicol. Lett..

[154] Feng Y., Zhang P., Zhang Z., Shi J., Jiao Z., Shao B. (2016). Endocrine Disrupting Effects of Triclosan on the Placenta in Pregnant Rats. PLoS ONE.

[155] Cao X., Hua X., Wang X., Chen L. (2017). Exposure of pregnant mice to triclosan impairs placental development and nutrient transport. Sci. Rep..

[156] Wang X., Chen X., Feng X., Chang F., Chen M., Xia Y., Chen L. (2015). Triclosan causes spontaneous abortion accompanied by decline of estrogen sulfotransferase activity in humans and mice. Sci. Rep..

[157] Frederiksen H., Taxvig C., Hass U., Vinggaard A.M., Nellemann C. (2008). Higher levels of ethyl paraben and butyl paraben in rat amniotic fluid than in maternal plasma after subcutaneous administration. Toxicol. Sci. Off. J. Soc. Toxicol..

[158] Dagher J.B., Hahn-Townsend C.K., Kaimal A., Mansi M.A., Henriquez J.E., Tran D.G., Laurent C.R., Bacak C.J., Buechter H.E., Cambric C. (2021). Independent and combined effects of Bisphenol A and Diethylhexyl Phthalate on gestational outcomes and offspring development in Sprague-Dawley rats. Chemosphere.

[159] Reed J.M., Spinelli P., Falcone S., He M., Goeke C.M., Susiarjo M. (2022). Evaluating the Effects of BPA and TBBPA Exposure on Pregnancy Loss and Maternal-Fetal Immune Cells in Mice. Environ. Health Perspect..

[160] Hua X., Xiong J.W., Zhang Y.J., Cao X.Y., Sun P., Wu J., Chen L. (2019). Exposure of pregnant mice to triclosan causes hyperphagic obesity of offspring via the hypermethylation of proopiomelanocortin promoter. Arch. Toxicol..

[161] Li J., Quan X., Zhang Y., Yu T., Lei S., Huang Z., Wang Q., Song W., Yang X., Xu P. (2021). PPARγ Regulates Triclosan Induced Placental Dysfunction. Cells.

[162] Bitencourt G., Fortunato E.D., Panis C., Amorim E.M.P., de Arruda Amorim J.P. (2019). Maternal exposure to triclosan causes fetal development restriction, deregulation of the oestrous cycle, and alters uterine tissue in rat offspring. Environ. Toxicol..

[163] Shen R., Zhao L.L., Yu Z., Zhang C., Chen Y.H., Wang H., Zhang Z.H., Xu D.X. (2017). Maternal di-(2-ethylhexyl) phthalate exposure during pregnancy causes fetal growth restriction in a stage-specific but gender-independent manner. Reprod. Toxicol..

[164] Zong T., Lai L., Hu J., Guo M., Li M., Zhang L., Zhong C., Yang B., Wu L., Zhang D. (2015). Maternal exposure to di-(2-ethylhexyl) phthalate disrupts placental growth and development in pregnant mice. J. Hazard. Mater..

[165] Xu W., Wu H., Shang L. (2021). Gene expression in rat placenta after exposure to di(2-ethylhexyl) phthalate. Hum. Exp. Toxicol..

[166] Xu Y., Agrawal S., Cook T.J., Knipp G.T. (2008). Maternal di-(2-ethylhexyl)-phthalate exposure influences essential fatty acid homeostasis in rat placenta. Placenta.

[167] Ahbab M.A., Güven C., Koçkaya E.A., Barlas N. (2017). Comparative developmental toxicity evaluation of di- n-hexyl phthalate and dicyclohexyl phthalate in rats. Toxicol. Ind. Health.

[168] Daston G.P. (2004). Developmental toxicity evaluation of butylparaben in Sprague-Dawley rats. Birth Defects Res. Part B Dev. Reprod. Toxicol..

[169] Shaw J., deCatanzaro D. (2009). Estrogenicity of parabens revisited: Impact of parabens on early pregnancy and an uterotrophic assay in mice. Reprod. Toxicol..

[170] Boberg J., Metzdorff S., Wortziger R., Axelstad M., Brokken L., Vinggaard A.M., Dalgaard M., Nellemann C. (2008). Impact of diisobutyl phthalate and other PPAR agonists on steroidogenesis and plasma insulin and leptin levels in fetal rats. Toxicology.

[171] Wang D., Li W., Yang C., Chen X., Liu X., He J., Tong C., Peng C., Ding Y., Geng Y. (2021). Exposure to ethylparaben and propylparaben interfere with embryo implantation by compromising endometrial decidualization in early pregnant mice. J. Appl. Toxicol. JAT.

[172] Berger K., Hyland C., Ames J.L., Mora A.M., Huen K., Eskenazi B., Holland N., Harley K.G. (2021). Prenatal Exposure to Mixtures of Phthalates, Parabens, and Other Phenols and Obesity in Five-Year-Olds in the CHAMACOS Cohort. Int. J. Environ. Res. Public Health.

[173] Vrijheid M., Fossati S., Maitre L., Márquez S., Roumeliotaki T., Agier L., Andrusaityte S., Cadiou S., Casas M., de Castro M. (2020). Early-Life Environmental Exposures and Childhood Obesity: An Exposome-Wide Approach. Environ. Health Perspect..

[174] Güil-Oumrait N., Cano-Sancho G., Montazeri P., Stratakis N., Warembourg C., Lopez-Espinosa M.J., Vioque J., Santa-Marina L., Jimeno-Romero A., Ventura R. (2022). Prenatal exposure to mixtures of phthalates and phenols and body mass index and blood pressure in Spanish preadolescents. Environ. Int..

[175] Ouidir M., Cissé A.H., Botton J., Lyon-Caen S., Thomsen C., Sakhi A.K., Sabaredzovic A., Bayat S., Slama R., Heude B. (2024). Fetal and Infancy Exposure to Phenols, Parabens, and Phthalates and Anthropometric Measurements up to 36 Months, in the Longitudinal SEPAGES Cohort. Environ. Health Perspect..

[176] Ouyang F., Zhang G.H., Du K., Shen L., Ma R., Wang X., Wang X., Zhang J. (2020). Maternal prenatal urinary bisphenol A level and child cardio-metabolic risk factors: A prospective cohort study. Environ. Pollut..

[177] Yang T.C., Peterson K.E., Meeker J.D., Sánchez B.N., Zhang Z., Cantoral A., Solano M., Tellez-Rojo M.M. (2017). Bisphenol A and phthalates in utero and in childhood: Association with child BMI z-score and adiposity. Environ. Res..

[178] Philippat C., Botton J., Calafat A.M., Ye X., Charles M.A., Slama R. (2014). Prenatal exposure to phenols and growth in boys. Epidemiology.

[179] Vafeiadi M., Roumeliotaki T., Myridakis A., Chalkiadaki G., Fthenou E., Dermitzaki E., Karachaliou M., Sarri K., Vassilaki M., Stephanou E.G. (2016). Association of early life exposure to bisphenol A with obesity and cardiometabolic traits in childhood. Environ. Res..

[180] Harley K.G., Aguilar Schall R., Chevrier J., Tyler K., Aguirre H., Bradman A., Holland N.T., Lustig R.H., Calafat A.M., Eskenazi B. (2013). Prenatal and postnatal bisphenol A exposure and body mass index in childhood in the CHAMACOS cohort. Environ. Health Perspect..

[181] Braun J.M., Li N., Arbuckle T.E., Dodds L., Massarelli I., Fraser W.D., Lanphear B.P., Muckle G. (2019). Association between gestational urinary bisphenol a concentrations and adiposity in young children: The MIREC study. Environ. Res..

[182] Guo J., Zhang J., Wu C., Xiao H., Lv S., Lu D., Qi X., Feng C., Liang W., Chang X. (2020). Urinary bisphenol A concentrations and adiposity measures at age 7 years in a prospective birth cohort. Chemosphere.

[183] Lee Y.M., Hong Y.C., Ha M., Kim Y., Park H., Kim H.S., Ha E.H. (2018). Prenatal Bisphenol-A exposure affects fetal length growth by maternal glutathione transferase polymorphisms, and neonatal exposure affects child volume growth by sex: From multiregional prospective birth cohort MOCEH study. Sci. Total Environ..

[184] Wu C., Li J., Xia W., Li Y., Zhang B., Zhou A., Hu J., Li C., Zhao H., Jiang M. (2018). The association of repeated measurements of prenatal exposure to triclosan with fetal and early-childhood growth. Environ. Int..

[185] Højsager F.D., Kyhl H.B., Frederiksen H., Juul A., Andersson A.M., Andersen M.S., Grøntved A., Jensen T.K. (2021). Prenatal Exposure to Butyl Paraben Is Associated With Fat Percentage in 7-Year-Old Boys. J. Clin. Endocrinol. Metab..

[186] Guo J., Wu C., Lu D., Jiang S., Liang W., Chang X., Xu H., Wang G., Zhou Z. (2017). Urinary paraben concentrations and their associations with anthropometric measures of children aged 3 years. Environ. Pollut..

[187] Buckley J.P., Engel S.M., Braun J.M., Whyatt R.M., Daniels J.L., Mendez M.A., Richardson D.B., Xu Y., Calafat A.M., Wolff M.S. (2016). Prenatal Phthalate Exposures and Body Mass Index Among 4- to 7-Year-old Children: A Pooled Analysis. Epidemiology.

[188] Lee D.W., Lim H.M., Lee J.Y., Min K.B., Shin C.H., Lee Y.A., Hong Y.C. (2022). Prenatal exposure to phthalate and decreased body mass index of children: A systematic review and meta-analysis. Sci. Rep..

[189] Li J., Qian X., Zhou Y., Li Y., Xu S., Xia W., Cai Z. (2021). Trimester-specific and sex-specific effects of prenatal exposure to di(2-ethylhexyl) phthalate on fetal growth, birth size, and early-childhood growth: A longitudinal prospective cohort study. Sci. Total Environ..

[190] Berman Y.E., Doherty D.A., Main K.M., Frederiksen H., Keelan J.A., Newnham J.P., Hart R.J. (2021). The influence of prenatal exposure to phthalates on subsequent male growth and body composition in adolescence. Environ. Res..

[191] Valvi D., Casas M., Romaguera D., Monfort N., Ventura R., Martinez D., Sunyer J., Vrijheid M. (2015). Prenatal Phthalate Exposure and Childhood Growth and Blood Pressure: Evidence from the Spanish INMA-Sabadell Birth Cohort Study. Environ. Health Perspect..

[192] Ferguson K.K., Bommarito P.A., Arogbokun O., Rosen E.M., Keil A.P., Zhao S., Barrett E.S., Nguyen R.H.N., Bush N.R., Trasande L. (2022). Prenatal Phthalate Exposure and Child Weight and Adiposity from in Utero to 6 Years of Age. Environ. Health Perspect..

[193] Harley K.G., Berger K., Rauch S., Kogut K., Claus Henn B., Calafat A.M., Huen K., Eskenazi B., Holland N. (2017). Association of prenatal urinary phthalate metabolite concentrations and childhood BMI and obesity. Pediatr. Res..

[194] Botton J., Philippat C., Calafat A.M., Carles S., Charles M.A., Slama R., The Eden Mother-Child Cohort Study G. (2016). Phthalate pregnancy exposure and male offspring growth from the intra-uterine period to five years of age. Environ. Res..

[195] Bowman A., Peterson K.E., Dolinoy D.C., Meeker J.D., Sánchez B.N., Mercado-Garcia A., Téllez-Rojo M.M., Goodrich J.M. (2019). Phthalate Exposures, DNA Methylation and Adiposity in Mexican Children Through Adolescence. Front. Public Health.

[196] Shoaff J., Papandonatos G.D., Calafat A.M., Ye X., Chen A., Lanphear B.P., Yolton K., Braun J.M. (2017). Early-Life Phthalate Exposure and Adiposity at 8 Years of Age. Environ. Health Perspect..

[197] Heggeseth B.C., Holland N., Eskenazi B., Kogut K., Harley K.G. (2019). Heterogeneity in childhood body mass trajectories in relation to prenatal phthalate exposure. Environ. Res..

[198] Vafeiadi M., Myridakis A., Roumeliotaki T., Margetaki K., Chalkiadaki G., Dermitzaki E., Venihaki M., Sarri K., Vassilaki M., Leventakou V. (2018). Association of Early Life Exposure to Phthalates With Obesity and Cardiometabolic Traits in Childhood: Sex Specific Associations. Front. Public Health.

[199] Kupsco A., Wu H., Calafat A.M., Kioumourtzoglou M.A., Cantoral A., Tamayo-Ortiz M., Pantic I., Pizano-Zárate M.L., Oken E., Braun J.M. (2022). Prenatal maternal phthalate exposures and trajectories of childhood adiposity from four to twelve years. Environ. Res..

[200] Buckley J.P., Engel S.M., Mendez M.A., Richardson D.B., Daniels J.L., Calafat A.M., Wolff M.S., Herring A.H. (2016). Prenatal Phthalate Exposures and Childhood Fat Mass in a New York City Cohort. Environ. Health Perspect..

[201] Gao H., Geng M.L., Gan H., Huang K., Zhang C., Zhu B.B., Sun L., Wu X., Zhu P., Tao F.B. (2022). Prenatal single and combined exposure to phthalates associated with girls’ BMI trajectory in the first six years. Ecotoxicol. Environ. Saf..

[202] Güil-Oumrait N., Stratakis N., Maitre L., Anguita-Ruiz A., Urquiza J., Fabbri L., Basagaña X., Heude B., Haug L.S., Sakhi A.K. (2024). Prenatal Exposure to Chemical Mixtures and Metabolic Syndrome Risk in Children. JAMA Netw. Open.

[203] Kalloo G., Calafat A.M., Chen A., Yolton K., Lanphear B.P., Braun J.M. (2018). Early life Triclosan exposure and child adiposity at 8 Years of age: A prospective cohort study. Environ. Health.

[204] Lee D.W., Lim Y.H., Shin C.H., Lee Y.A., Kim B.N., Kim J.I., Hong Y.C. (2020). Prenatal exposure to di-(2-ethylhexyl) phthalate and decreased skeletal muscle mass in 6-year-old children: A prospective birth cohort study. Environ. Res..

[205] Maresca M.M., Hoepner L.A., Hassoun A., Oberfield S.E., Mooney S.J., Calafat A.M., Ramirez J., Freyer G., Perera F.P., Whyatt R.M. (2016). Prenatal Exposure to Phthalates and Childhood Body Size in an Urban Cohort. Environ. Health Perspect..

[206] Nidens N., Krönke A., Jurkutat A., Schlingmann M., Poulain T., Nüchter M., Kiviranta H., Körner A., Vogel M., Lindh C. (2021). Associations of prenatal exposure to phthalates and one phthalate substitute with anthropometric measures in early life: Results from the German LIFE Child cohort study. Best Pract. Res. Clin. Endocrinol. Metab..

[207] de Freitas T., Zapaterini J.R., Moreira C.M., de Aquino A.M., Alonso-Costa L.G., Bidinotto L.T., Kass L., Flaws J.A., Scarano W.R., Barbisan L.F. (2021). Prenatal exposure to a mixture of different phthalates increases the risk of mammary carcinogenesis in F1 female offspring. Food Chem. Toxicol..

[208] Mustieles V., Rolland M., Pin I., Thomsen C., Sakhi A.K., Sabaredzovic A., Muckle G., Guichardet K., Slama R., Philippat C. (2023). Early-Life Exposure to a Mixture of Phenols and Phthalates in Relation to Child Social Behavior: Applying an Evidence-Based Prioritization to a Cohort with Improved Exposure Assessment. Environ. Health Perspect..

[209] Malaisé Y., Lencina C., Cartier C., Olier M., Ménard S., Guzylack-Piriou L. (2020). Perinatal oral exposure to low doses of bisphenol A, S or F impairs immune functions at intestinal and systemic levels in female offspring mice. Environ. Health A Glob. Access Sci. Source.

[210] da Silva B.S., Pietrobon C.B., Bertasso I.M., Lopes B.P., Carvalho J.C., Peixoto-Silva N., Santos T.R., Claudio-Neto S., Manhães A.C., Oliveira E. (2019). Short and long-term effects of bisphenol S (BPS) exposure during pregnancy and lactation on plasma lipids, hormones, and behavior in rats. Environ. Pollut..

[211] Taylor J.A., Sommerfeld-Sager J.M., Meng C.X., Nagel S.C., Shioda T., Vom Saal F.S. (2018). Reduced body weight at weaning followed by increased post-weaning growth rate interacts with part-per-trillion fetal serum concentrations of bisphenol A (BPA) to impair glucose tolerance in male mice. PLoS ONE.

[212] Boronat-Belda T., Ferrero H., Al-Abdulla R., Quesada I., Gustafsson J.A., Nadal Á., Alonso-Magdalena P. (2020). Bisphenol-A exposure during pregnancy alters pancreatic β-cell division and mass in male mice offspring: A role for ERβ. Food Chem. Toxicol..

[213] Bansal A., Li C., Xin F., Duemler A., Li W., Rashid C., Bartolomei M.S., Simmons R.A. (2019). Transgenerational effects of maternal bisphenol: A exposure on offspring metabolic health. J. Dev. Orig. Health Dis..

[214] Rashid C.S., Bansal A., Mesaros C., Bartolomei M.S., Simmons R.A. (2020). Paternal bisphenol A exposure in mice impairs glucose tolerance in female offspring. Food Chem. Toxicol..

[215] Nguyen H.T., Li L., Eguchi A., Kannan K., Kim E.Y., Iwata H. (2021). Effects on the liver lipidome of rat offspring prenatally exposed to bisphenol A. Sci. Total Environ..

[216] Nguyen H.T., Yamamoto K., Iida M., Agusa T., Ochiai M., Guo J., Karthikraj R., Kannan K., Kim E.Y., Iwata H. (2020). Effects of prenatal bisphenol A exposure on the hepatic transcriptome and proteome in rat offspring. Sci. Total Environ..

[217] Veiga-Lopez A., Moeller J., Sreedharan R., Singer K., Lumeng C., Ye W., Pease A., Padmanabhan V. (2016). Developmental programming: Interaction between prenatal BPA exposure and postnatal adiposity on metabolic variables in female sheep. Am. J. Physiol. Endocrinol. Metab..

[218] Puttabyatappa M., Martin J.D., Andriessen V., Stevenson M., Zeng L., Pennathur S., Padmanabhan V. (2019). Developmental programming: Changes in mediators of insulin sensitivity in prenatal bisphenol A-treated female sheep. Reprod. Toxicol..

[219] Puttabyatappa M., Saadat N., Elangovan V.R., Dou J., Bakulski K., Padmanabhan V. (2022). Developmental programming: Impact of prenatal bisphenol-A exposure on liver and muscle transcriptome of female sheep. Toxicol. Appl. Pharmacol..

[220] Ciarelli J., Thangaraj S.V., Sun H., Domke S., Alkhatib B., Vyas A.K., Gregg B., Sargis R.M., Padmanabhan V. (2024). Developmental programming: An exploratory analysis of pancreatic islet compromise in female sheep resulting from gestational BPA exposure. Mol. Cell. Endocrinol..

[221] García-Arévalo M., Alonso-Magdalena P., Servitja J.M., Boronat-Belda T., Merino B., Villar-Pazos S., Medina-Gómez G., Novials A., Quesada I., Nadal A. (2016). Maternal Exposure to Bisphenol-A During Pregnancy Increases Pancreatic β-Cell Growth During Early Life in Male Mice Offspring. Endocrinology.

[222] Alonso-Magdalena P., Vieira E., Soriano S., Menes L., Burks D., Quesada I., Nadal A. (2010). Bisphenol A exposure during pregnancy disrupts glucose homeostasis in mothers and adult male offspring. Environ. Health Perspect..

[223] Esteban J., Serrano-Maciá M., Sánchez-Pérez I., Alonso-Magdalena P., Pellín M.C., García-Arévalo M., Nadal Á., Barril J. (2019). In utero exposure to bisphenol-A disrupts key elements of retinoid system in male mice offspring. Food Chem. Toxicol..

[224] Luo S., Li Y., Li Y., Zhu Q., Jiang J., Wu C., Shen T. (2016). Gestational and lactational exposure to low-dose bisphenol A increases Th17 cells in mice offspring. Environ. Toxicol. Pharmacol..

[225] O’Brien E., Dolinoy D.C., Mancuso P. (2014). Perinatal bisphenol A exposures increase production of pro-inflammatory mediators in bone marrow-derived mast cells of adult mice. J. Immunotoxicol..

[226] Susiarjo M., Xin F., Bansal A., Stefaniak M., Li C., Simmons R.A., Bartolomei M.S. (2015). Bisphenol a exposure disrupts metabolic health across multiple generations in the mouse. Endocrinology.

[227] van Esterik J.C., Dollé M.E., Lamoree M.H., van Leeuwen S.P., Hamers T., Legler J., van der Ven L.T. (2014). Programming of metabolic effects in C57BL/6JxFVB mice by exposure to bisphenol A during gestation and lactation. Toxicology.

[228] Bodin J., Bølling A.K., Becher R., Kuper F., Løvik M., Nygaard U.C. (2014). Transmaternal bisphenol A exposure accelerates diabetes type 1 development in NOD mice. Toxicol. Sci. Off. J. Soc. Toxicol..

[229] Anderson O.S., Peterson K.E., Sanchez B.N., Zhang Z., Mancuso P., Dolinoy D.C. (2013). Perinatal bisphenol A exposure promotes hyperactivity, lean body composition, and hormonal responses across the murine life course. FASEB J..

[230] Rabaglino M.B., Moreira-Espinoza M.J., Lopez J.P., Garcia N.H., Beltramo D. (2016). Maternal Triclosan consumption alters the appetite regulatory network on Wistar rat offspring and predispose to metabolic syndrome in the adulthood. Endocr. J..

[231] Ma Y., Guo Y., Ye H., Zhang J., Ke Y. (2020). Perinatal Triclosan exposure in the rat induces long-term disturbances in metabolism and gut microbiota in adulthood and old age. Environ. Res..

[232] Weber A.A., Yang X., Mennillo E., Ding J., Watrous J.D., Jain M., Chen S., Karin M., Tukey R.H. (2022). Lactational delivery of Triclosan promotes non-alcoholic fatty liver disease in newborn mice. Nat. Commun..

[233] Vo T.T., Nguyen P.V., Duong H.T., Nguyen T.D., Huynh H.T., Nong H.V. (2015). Potential effect of combined xenoestrogens during gestation stages on mouse offspring. J. Environ. Biol..

[234] Leppert B., Strunz S., Seiwert B., Schlittenbauer L., Schlichting R., Pfeiffer C., Röder S., Bauer M., Borte M., Stangl G.I. (2020). Maternal paraben exposure triggers childhood overweight development. Nat. Commun..

[235] Wassenaar P.N.H., Legler J. (2017). Systematic review and meta-analysis of early life exposure to di(2-ethylhexyl) phthalate and obesity related outcomes in rodents. Chemosphere.

[236] Lee K.I., Chiang C.W., Lin H.C., Zhao J.F., Li C.T., Shyue S.K., Lee T.S. (2016). Maternal exposure to di-(2-ethylhexyl) phthalate exposure deregulates blood pressure, adiposity, cholesterol metabolism and social interaction in mouse offspring. Arch. Toxicol..

[237] Neier K., Montrose L., Chen K., Malloy M.A., Jones T.R., Svoboda L.K., Harris C., Song P.X.K., Pennathur S., Sartor M.A. (2020). Short- and long-term effects of perinatal phthalate exposures on metabolic pathways in the mouse liver. Environ. Epigenetics.

[238] Fan Y., Qin Y., Chen M., Li X., Wang R., Huang Z., Xu Q., Yu M., Zhang Y., Han X. (2020). Prenatal low-dose DEHP exposure induces metabolic adaptation and obesity: Role of hepatic thiamine metabolism. J. Hazard. Mater..

[239] Sanders A.P., Saland J.M., Wright R.O., Satlin L. (2018). Perinatal and childhood exposure to environmental chemicals and blood pressure in children: A review of literature 2007–2017. Pediatr. Res..

[240] An S.J., Lee E.J., Jeong S.H., Hong Y.P., Ahn S., Yang Y.J. (2021). Perinatal exposure to di-(2-ethylhexyl) phthalate induces hepatic lipid accumulation mediated by diacylglycerol acyltransferase 1. Hum. Exp. Toxicol..

[241] Li G., Zhao C.Y., Wu Q., Kang Z., Zhang J.T., Guan S.Y., Jin H.W., Zhang Y.B., Na X.L. (2022). Di(2-ethylhexyl) phthalate disturbs cholesterol metabolism through oxidative stress in rat liver. Environ. Toxicol. Pharmacol..

[242] Yang Y.J., Park M.S., Lee E.J., Hong Y.P. (2018). The Development of Metabolic Derangement in Male Offspring after Perinatal Exposure to Di-(2-Ethylhexyl) Phthalate. Biomed. Environ. Sci. BES.

[243] Venturelli A.C., Meyer K.B., Fischer S.V., Kita D.H., Philipsen R.A., Morais R.N., Martino Andrade A.J. (2019). Effects of in utero and lactational exposure to phthalates on reproductive development and glycemic homeostasis in rats. Toxicology.

[244] Rajesh P., Balasubramanian K. (2015). Gestational exposure to di(2-ethylhexyl) phthalate (DEHP) impairs pancreatic β-cell function in F1 rat offspring. Toxicol. Lett..

[245] Rajagopal G., Bhaskaran R.S., Karundevi B. (2019). Maternal di-(2-ethylhexyl) phthalate exposure alters hepatic insulin signal transduction and glucoregulatory events in rat F(1) male offspring. J. Appl. Toxicol. JAT.

[246] Dong J., Cong Z., You M., Fu Y., Wang Y., Wang Y., Fu H., Wei L., Chen J. (2019). Effects of perinatal di (2-ethylhexyl) phthalate exposure on thyroid function in rat offspring. Environ. Toxicol. Pharmacol..

[247] Zhang Y., Zhang W., Fu X., Zhou F., Yu H., Na X. (2018). Transcriptomics and metabonomics analyses of maternal DEHP exposure on male offspring. Environ. Sci. Pollut. Res. Int..

[248] Ma S., Li R., Gong X., Shi W., Zhong X. (2018). Lycopene reduces in utero bisphenol A exposure-induced mortality, benefits hormones, and development of reproductive organs in offspring mice. Environ. Sci. Pollut. Res. Int..

[249] Shu L., Meng Q., Diamante G., Tsai B., Chen Y.W., Mikhail A., Luk H., Ritz B., Allard P., Yang X. (2019). Prenatal Bisphenol A Exposure in Mice Induces Multitissue Multiomics Disruptions Linking to Cardiometabolic Disorders. Endocrinology.

[250] Yang Q., Mao Y., Wang J., Yu H., Zhang X., Pei X., Duan Z., Xiao C., Ma M. (2022). Gestational bisphenol A exposure impairs hepatic lipid metabolism by altering mTOR/CRTC2/SREBP1 in male rat offspring. Hum. Exp. Toxicol..

[251] MohanKumar S.M., Rajendran T.D., Vyas A.K., Hoang V., Asirvatham-Jeyaraj N., Veiga-Lopez A., Olivier N.B., Padmanabhan V., MohanKumar P.S. (2017). Effects of prenatal bisphenol-A exposure and postnatal overfeeding on cardiovascular function in female sheep. J. Dev. Orig. Health Dis..

[252] Nakashima R., Hayashi Y., Md K., Jia X., Wang D., Naito H., Ito Y., Kamijima M., Gonzalez F.J., Nakajima T. (2013). Exposure to DEHP decreased four fatty acid levels in plasma of prepartum mice. Toxicology.

[253] Svoboda L.K., Wang K., Cavalcante R.G., Neier K., Colacino J.A., Sartor M.A., Dolinoy D.C. (2020). Sex-Specific Programming of Cardiac DNA Methylation by Developmental Phthalate Exposure. Epigenetics Insights.

[254] Li F., Luo T., Rong H., Lu L., Zhang L., Zheng C., Yi D., Peng Y., Lei E., Xiong X. (2022). Maternal rodent exposure to di-(2-ethylhexyl) phthalate decreases muscle mass in the offspring by increasing myostatin. J. Cachexia Sarcopenia Muscle.

[255] Dong X.W., Wu W.D., Yao S.Q., Zhang Y.B., Wang C., Wu H.Y., Na X.L. (2021). Long-term Di-(2-ethylhexyl) phthalate Exposure Disturbs the Lipid Metabolism Profiles and Hepatic Enzymes in Male Rats: A UPLC-MS-based Serum Metabolomics Analysis. Biomed. Environ. Sci. BES.

[256] Martinez-Arguelles D.B., McIntosh M., Rohlicek C.V., Culty M., Zirkin B.R., Papadopoulos V. (2013). Maternal in utero exposure to the endocrine disruptor di-(2-ethylhexyl) phthalate affects the blood pressure of adult male offspring. Toxicol. Appl. Pharmacol..

[257] Martinez-Arguelles D.B., Guichard T., Culty M., Zirkin B.R., Papadopoulos V. (2011). In utero exposure to the antiandrogen di-(2-ethylhexyl) phthalate decreases adrenal aldosterone production in the adult rat. Biol. Reprod..

[258] Parsanathan R., Maria Joseph A., Karundevi B. (2019). Postnatal exposure to di-(2-ethylhexyl)phthalate alters cardiac insulin signaling molecules and GLUT4(Ser488) phosphorylation in male rat offspring. J. Cell. Biochem..

[259] Martinez-Arguelles D.B., Campioli E., Lienhart C., Fan J., Culty M., Zirkin B.R., Papadopoulos V. (2014). In utero exposure to the endocrine disruptor di-(2-ethylhexyl) phthalate induces long-term changes in gene expression in the adult male adrenal gland. Endocrinology.

[260] Wei Z., Song L., Wei J., Chen T., Chen J., Lin Y., Xia W., Xu B., Li X., Chen X. (2012). Maternal exposure to di-(2-ethylhexyl)phthalate alters kidney development through the renin-angiotensin system in offspring. Toxicol. Lett..

[261] Zhu Y.P., Chen L., Wang X.J., Jiang Q.H., Bei X.Y., Sun W.L., Xia S.J., Jiang J.T. (2017). Maternal exposure to di-n-butyl phthalate (DBP) induces renal fibrosis in adult rat offspring. Oncotarget.

[262] Sun W.L., Zhu Y.P., Ni X.S., Jing D.D., Yao Y.T., Ding W., Liu Z.H., Ding G.X., Jiang J.T. (2018). Potential involvement of Fgf10/Fgfr2 and androgen receptor (AR) in renal fibrosis in adult male rat offspring subjected to prenatal exposure to di-n-butyl phthalate (DBP). Toxicol. Lett..

[263] Ye Q., Zhao S., Zhang Y., Su Y.M., Chen M., Zhao J., Jia G.Z., Han B.M., Jiang J.T. (2020). Activation of the RhoA/ROCK pathway contributes to renal fibrosis in offspring rats induced by maternal exposure to di-n-butyl phthalate. Toxicology.

[264] Boberg J., Axelstad M., Svingen T., Mandrup K., Christiansen S., Vinggaard A.M., Hass U. (2016). Multiple Endocrine Disrupting Effects in Rats Perinatally Exposed to Butylparaben. Toxicol. Sci. Off. J. Soc. Toxicol..

[265] Zhao S., Pan L., Chen M., Zhu Y.P., Han B.M., Xia S.J., Jiang J.T. (2020). In utero di-n-butyl phthalate exposure induced abnormal autophagy in renal tubular cells via hedgehog signaling in newborn rats. Chem. Biol. Interact..

[266] Perng W., Watkins D.J., Cantoral A., Mercado-García A., Meeker J.D., Téllez-Rojo M.M., Peterson K.E. (2017). Exposure to phthalates is associated with lipid profile in peripubertal Mexican youth. Environ. Res..

[267] Warembourg C., Maitre L., Tamayo-Uria I., Fossati S., Roumeliotaki T., Aasvang G.M., Andrusaityte S., Casas M., Cequier E., Chatzi L. (2019). Early-Life Environmental Exposures and Blood Pressure in Children. J. Am. Coll. Cardiol..

[268] Philips E.M., Jaddoe V.W.V., Trasande L. (2017). Effects of early exposure to phthalates and bisphenols on cardiometabolic outcomes in pregnancy and childhood. Reprod. Toxicol..

[269] Tain Y.L., Hou C.Y., Chang-Chien G.P., Lin S., Hsu C.N. (2023). Resveratrol Butyrate Ester Supplementation Blunts the Development of Offspring Hypertension in a Maternal Di-2-ethylhexyl Phthalate Exposure Rat Model. Nutrients.

[270] Zhao S., Jiang J.T., Li D., Zhu Y.P., Xia S.J., Han B.M. (2019). Maternal exposure to di-n-butyl phthalate promotes Snail1-mediated epithelial-mesenchymal transition of renal tubular epithelial cells via upregulation of TGF-β1 during renal fibrosis in rat offspring. Ecotoxicol. Environ. Saf..

[271] Rahmani A., Soleimannejad K., Hafezi Ahmadi M.R., Asadollahi K., Khalighi Z. (2016). Prenatal Exposure to Phthalic Acid Induces Increased Blood Pressure, Oxidative Stress, and Markers of Endothelial Dysfunction in Rat Offspring. Cardiovasc. Toxicol..

[272] Hart R.J. (2020). The Impact of Prenatal Exposure to Bisphenol A on Male Reproductive Function. Front. Endocrinol..

[273] Dean A., Sharpe R.M. (2013). Clinical review: Anogenital distance or digit length ratio as measures of fetal androgen exposure: Relationship to male reproductive development and its disorders. J. Clin. Endocrinol. Metab..

[274] Mammadov E., Uncu M., Dalkan C. (2018). High Prenatal Exposure to Bisphenol A Reduces Anogenital Distance in Healthy Male Newborns. J. Clin. Res. Pediatr. Endocrinol..

[275] Sun X., Li D., Liang H., Miao M., Song X., Wang Z., Zhou Z., Yuan W. (2018). Maternal exposure to bisphenol A and anogenital distance throughout infancy: A longitudinal study from Shanghai, China. Environ. Int..

[276] Berger K., Eskenazi B., Kogut K., Parra K., Lustig R.H., Greenspan L.C., Holland N., Calafat A.M., Ye X., Harley K.G. (2018). Association of Prenatal Urinary Concentrations of Phthalates and Bisphenol A and Pubertal Timing in Boys and Girls. Environ. Health Perspect..

[277] Freire C., Castiello F., Babarro I., Anguita-Ruiz A., Casas M., Vrijheid M., Sarzo B., Beneito A., Kadawathagedara M., Philippat C. (2024). Association of prenatal exposure to phthalates and synthetic phenols with pubertal development in three European cohorts. Int. J. Hyg. Environ. Health.

[278] Ferguson K.K., Peterson K.E., Lee J.M., Mercado-García A., Blank-Goldenberg C., Téllez-Rojo M.M., Meeker J.D. (2014). Prenatal and peripubertal phthalates and bisphenol A in relation to sex hormones and puberty in boys. Reprod. Toxicol..

[279] Hart R.J., Doherty D.A., Keelan J.A., Minaee N.S., Thorstensen E.B., Dickinson J.E., Pennell C.E., Newnham J.P., McLachlan R., Norman R.J. (2018). The impact of antenatal Bisphenol A exposure on male reproductive function at 20–22 years of age. Reprod. Biomed. Online.

[280] Holmboe S.A., Scheutz Henriksen L., Frederiksen H., Andersson A.M., Priskorn L., Jørgensen N., Juul A., Toppari J., Skakkebæk N.E., Main K.M. (2022). Prenatal exposure to phenols and benzophenones in relation to markers of male reproductive function in adulthood. Front. Endocrinol..

[281] Wang C., Chen L., Zhao S., Hu Y., Zhou Y., Gao Y., Wang W., Zhang J., Tian Y. (2018). Impacts of prenatal triclosan exposure on fetal reproductive hormones and its potential mechanism. Environ. Int..

[282] Harley K.G., Berger K.P., Kogut K., Parra K., Lustig R.H., Greenspan L.C., Calafat A.M., Ye X., Eskenazi B. (2019). Association of phthalates, parabens and phenols found in personal care products with pubertal timing in girls and boys. Hum. Reprod..

[283] Swan S.H., Main K.M., Liu F., Stewart S.L., Kruse R.L., Calafat A.M., Mao C.S., Redmon J.B., Ternand C.L., Sullivan S. (2005). Decrease in anogenital distance among male infants with prenatal phthalate exposure. Environ. Health Perspect..

[284] Sathyanarayana S., Grady R., Barrett E.S., Redmon B., Nguyen R.H.N., Barthold J.S., Bush N.R., Swan S.H. (2016). First trimester phthalate exposure and male newborn genital anomalies. Environ. Res..

[285] Romao R.L.P., Dodds L., Ashley-Martin J., Monnier P., Arbuckle T.E. (2020). Prenatal exposure to phthalates and male reproductive system development: Results from a Canadian pregnancy cohort study. Reprod. Toxicol..

[286] Freire C., Castiello F., Lopez-Espinosa M.J., Beneito A., Lertxundi A., Jimeno-Romero A., Vrijheid M., Casas M. (2022). Association of prenatal phthalate exposure with pubertal development in Spanish boys and girls. Environ. Res..

[287] Watkins D.J., Sánchez B.N., Téllez-Rojo M.M., Lee J.M., Mercado-García A., Blank-Goldenberg C., Peterson K.E., Meeker J.D. (2017). Phthalate and bisphenol A exposure during in utero windows of susceptibility in relation to reproductive hormones and pubertal development in girls. Environ. Res..

[288] Henriksen L.S., Frederiksen H., Jørgensen N., Juul A., Skakkebæk N.E., Toppari J., Petersen J.H., Main K.M. (2023). Maternal phthalate exposure during pregnancy and testis function of young adult sons. Sci. Total Environ..

[289] Olukole S.G., Lanipekun D.O., Ola-Davies E.O., Oke B.O. (2019). Maternal exposure to environmentally relevant doses of bisphenol A causes reproductive dysfunction in F1 adult male rats: Protective role of melatonin. Environ. Sci. Pollut. Res. Int..

[290] Silva B.S., Bertasso I.M., Pietrobon C.B., Lopes B.P., Santos T.R., Peixoto-Silva N., Carvalho J.C., Claudio-Neto S., Manhães A.C., Cabral S.S. (2019). Effects of maternal bisphenol A on behavior, sex steroid and thyroid hormones levels in the adult rat offspring. Life Sci..

[291] Campos P., Oliveira I.M., Sena de Souza J., Da Conceição R.R., Giannocco G., Chiamolera M.I., Silva M.R.D., Romano M.A., Romano R.M. (2019). Maternal bisphenol A exposure disrupts spermatogenesis in adult rat offspring. J. Toxicol. Environ. Health Part A.

[292] Salian S., Doshi T., Vanage G. (2009). Perinatal exposure of rats to Bisphenol A affects the fertility of male offspring. Life Sci..

[293] Dere E., Anderson L.M., Huse S.M., Spade D.J., McDonnell-Clark E., Madnick S.J., Hall S.J., Camacho L., Lewis S.M., Vanlandingham M.M. (2018). Effects of continuous bisphenol A exposure from early gestation on 90 day old rat testes function and sperm molecular profiles: A CLARITY-BPA consortium study. Toxicol. Appl. Pharmacol..

[294] Wei Y., Han C., Geng Y., Cui Y., Bao Y., Shi W., Zhong X. (2019). Maternal exposure to bisphenol A during pregnancy interferes testis development of F1 male mice. Environ. Sci. Pollut. Res. Int..

[295] Zhang S., Bao J., Gong X., Shi W., Zhong X. (2019). Hazards of bisphenol A—blocks RNA splicing leading to abnormal testicular development in offspring male mice. Chemosphere.

[296] Ma S., Shi W., Wang X., Song P., Zhong X. (2017). Bisphenol A Exposure during Pregnancy Alters the Mortality and Levels of Reproductive Hormones and Genes in Offspring Mice. BioMed. Res. Int..

[297] Ortega-García A.P., Díaz-Hernández V., Collazo-Saldaña P., Merchant-Larios H. (2021). Bisphenol A alters differentiation of Leydig cells in the rabbit fetal testis. Int. J. Dev. Biol..

[298] Higuchi T.T., Palmer J.S., Gray L.E., Veeramachaneni D.N. (2003). Effects of dibutyl phthalate in male rabbits following in utero, adolescent, or postpubertal exposure. Toxicol. Sci. Off. J. Soc. Toxicol..

[299] Dostalova P., Zatecka E., Ded L., Elzeinova F., Valaskova E., Kubatova A., Korenkova V., Langerova L., Komrskova K., Peknicova J. (2020). Gestational and pubertal exposure to low dose of di-(2-ethylhexyl) phthalate impairs sperm quality in adult mice. Reprod. Toxicol..

[300] Wilson V.S., Howdeshell K.L., Lambright C.S., Furr J., Earl Gray L. (2007). Differential expression of the phthalate syndrome in male Sprague-Dawley and Wistar rats after in utero DEHP exposure. Toxicol. Lett..

[301] Hannas B.R., Lambright C.S., Furr J., Howdeshell K.L., Wilson V.S., Gray L.E. (2011). Dose-response assessment of fetal testosterone production and gene expression levels in rat testes following in utero exposure to diethylhexyl phthalate, diisobutyl phthalate, diisoheptyl phthalate, and diisononyl phthalate. Toxicol. Sci. Off. J. Soc. Toxicol..

[302] Repouskou A., Panagiotidou E., Panagopoulou L., Bisting P.L., Tuck A.R., Sjödin M.O.D., Lindberg J., Bozas E., Rüegg J., Gennings C. (2019). Gestational exposure to an epidemiologically defined mixture of phthalates leads to gonadal dysfunction in mouse offspring of both sexes. Sci. Rep..

[303] Howdeshell K.L., Furr J., Lambright C.R., Rider C.V., Wilson V.S., Gray L.E. (2007). Cumulative effects of dibutyl phthalate and diethylhexyl phthalate on male rat reproductive tract development: Altered fetal steroid hormones and genes. Toxicol. Sci. Off. J. Soc. Toxicol..

[304] Zhang L.D., Deng Q., Wang Z.M., Gao M., Wang L., Chong T., Li H.C. (2013). Disruption of reproductive development in male rat offspring following gestational and lactational exposure to di-(2-ethylhexyl) phthalate and genistein. Biol. Res..

[305] Fiandanese N., Borromeo V., Berrini A., Fischer B., Schaedlich K., Schmidt J.S., Secchi C., Pocar P. (2016). Maternal exposure to a mixture of di(2-ethylhexyl) phthalate (DEHP) and polychlorinated biphenyls (PCBs) causes reproductive dysfunction in adult male mouse offspring. Reprod. Toxicol..

[306] Abdel-Maksoud F.M., Ali F.A.Z., Akingbemi B.T. (2019). Prenatal exposures to bisphenol A and di (2-ethylhexyl) phthalate disrupted seminiferous tubular development in growing male rats. Reprod. Toxicol..

[307] Wei Y., Han C., Li S., Cui Y., Bao Y., Shi W. (2020). Maternal exposure to bisphenol A during pregnancy interferes ovaries development of F1 female mice. Theriogenology.

[308] Meng Y., Yannan Z., Ren L., Qi S., Wei W., Lihong J. (2020). Adverse reproductive function induced by maternal BPA exposure is associated with abnormal autophagy and activating inflamation via mTOR and TLR4/NF-κB signaling pathways in female offspring rats. Reprod. Toxicol..

[309] Hannon P.R., Flaws J.A. (2015). The effects of phthalates on the ovary. Front. Endocrinol..

[310] Li L., Zhang T., Qin X.S., Ge W., Ma H.G., Sun L.L., Hou Z.M., Chen H., Chen P., Qin G.Q. (2014). Exposure to diethylhexyl phthalate (DEHP) results in a heritable modification of imprint genes DNA methylation in mouse oocytes. Mol. Biol. Rep..

[311] Pocar P., Fiandanese N., Secchi C., Berrini A., Fischer B., Schmidt J.S., Schaedlich K., Borromeo V. (2012). Exposure to di(2-ethyl-hexyl) phthalate (DEHP) in utero and during lactation causes long-term pituitary-gonadal axis disruption in male and female mouse offspring. Endocrinology.

[312] Perrier F., Giorgis-Allemand L., Slama R., Philippat C. (2016). Within-subject Pooling of Biological Samples to Reduce Exposure Misclassification in Biomarker-based Studies. Epidemiology.

[313] Malassine A., Frendo J.L., Evain-Brion D. (2003). A comparison of placental development and endocrine functions between the human and mouse model. Hum. Reprod. Update.

